# Outcomes reported in randomized controlled trials of traditional Chinese medicine for chronic pharyngitis: a scoping review

**DOI:** 10.3389/fphar.2026.1958608

**Published:** 2026-10-01

**Authors:** Danni Li, Chenyao Zhang, Tongyang Jia, Xinyao Jin, Fengwen Yang, Wentai Pang, Junhua Zhang

**Affiliations:** 1 Tianjin University of Traditional Chinese Medicine Evidence Based Medicine Center, Tianjin, China; 2 National Key Laboratory of Modern Chinese Medicine Creation, Tianjin, China

**Keywords:** chronic pharyngitis, core outcome set, outcomes, randomized controlled trials, traditional Chinese medicine

## Abstract

**Purpose:**

Evidence synthesis of traditional Chinese medicine (TCM) interventions for chronic pharyngitis is hindered by substantial variation in the outcomes selected and reported across clinical trials. This scoping review aimed to systematically map the outcomes reported in randomized controlled trials (RCTs) and to characterize patterns and gaps in outcome reporting. The findings were intended to provide an empirical basis for the subsequent development of a core outcome set (COS) for chronic pharyngitis.

**Methods:**

A systematic literature search was conducted to identify RCTs evaluating TCM interventions in patients with chronic pharyngitis. For each eligible trial, study characteristics, reported outcomes, and assessment time points were charted using a structured approach. The identified outcomes were classified into predefined outcome domains and summarized using descriptive and narrative synthesis.

**Results:**

A total of 320 eligible RCTs were included, from which 1,204 individual outcomes were identified and classified into seven domains. Considerable variation was observed in outcome selection, with most individual outcomes reported in relatively few trials. Clinical symptoms and signs were the most frequently assessed outcomes, whereas health-related quality of life, economic outcomes, and long-term outcomes were rarely evaluated. Overall, outcome assessment was predominantly focused on short-term treatment effects, while adverse events, patient-reported outcomes, and extended follow-up remained insufficiently represented. Notably, none of the 69 trials reporting adverse reactions clearly specified the time points at which these outcomes were assessed.

**Conclusion:**

Substantial heterogeneity in outcome selection, measurement, and reporting limits the comparability of RCTs evaluating TCM interventions for chronic pharyngitis and complicates evidence synthesis. By systematically characterizing current outcome practices and identifying important reporting gaps, this review provides an empirical foundation for the development of a COS for chronic pharyngitis. Adoption of a COS could promote greater consistency in outcome selection, measurement, and reporting, thereby improving the comparability, transparency, and clinical relevance of future trials.

## 1 Introduction

Chronic inflammation of the pharyngeal mucosa is frequently encountered in clinical practice, yet consensus regarding its diagnostic criteria and therapeutic management remains insufficient, underscoring the need for additional clinical investigation (Tu et al., 2026; Zhang et al., 2023). In the United States, pharyngitis-related complaints have been estimated to result in approximately 7–11 million outpatient consultations each year (Li et al., 2019), contributing to a substantial burden on healthcare services and negatively influencing patients’ health-related quality of life and everyday functioning (Zhang et al., 2023; Salkind and Wright, 2008). Persistent throat irritation and discomfort are among the most characteristic manifestations of the condition (Zhang et al., 2023). Moreover, prolonged inflammation in the pharyngeal region may coexist with or contribute to laryngeal disorders, such as chronic laryngitis and impaired voice function (Brown et al., 2026; Lechien et al., 2017; Jetté, 2016; Yazici et al., 2010).

In view of its recurrent course and considerable clinical burden (Li et al., 2019), chronic pharyngitis has prompted growing interest in traditional Chinese medicine (TCM) interventions as potential therapeutic approaches, leading to an increasing number of clinical studies in this field (Wu et al., 2024; Mu et al., 2026; Li et al., 2017; Ma et al., 2023; Wang et al., 2025). These interventions are intended to alleviate persistent pharyngeal discomfort, attenuate inflammation of the pharyngeal mucosa, relieve local irritation, and improve symptoms associated with excessive voice use or laryngopharyngeal reflux (Li et al., 2024). Relevant trials have been conducted in a variety of healthcare settings, ranging from community and primary care facilities to outpatient clinics and hospital departments. Accordingly, treatment effects have been assessed using diverse outcomes and measurement instruments, including symptom severity, findings on pharyngeal examination, inflammatory biomarkers, recurrence, and health-related quality of life (Shen et al., 2022). This diversity reflects the multidimensional clinical manifestations of chronic pharyngitis but may also contribute to inconsistency in outcome selection and reporting across trials.

Evidence concerning the effectiveness of traditional Chinese medicine (TCM) interventions for chronic pharyngitis has been evaluated in several systematic reviews (Wu et al., 2024; Mu et al., 2026; Li et al., 2017). Nevertheless, integrating and quantitatively combining results from the primary trials remains challenging because studies differ substantially in how outcomes are defined, measured, and reported (Young et al., 2019; Cao et al., 2024; Gargon, 2016). Although these discrepancies may partly reflect variation in therapeutic objectives, intervention approaches, and healthcare contexts, they substantially reduce comparability across studies and limit the reliability of evidence synthesis. Outcome selection in clinical trials therefore requires careful prioritization to ensure that clinically meaningful treatment effects are adequately captured without creating excessive demands on study feasibility, participants, investigators, available resources, or statistical analysis (Saldanha et al., 2020; Matvienko-Sikar et al., 2022; Redulla, 2016; Dwan et al., 2013).

A core outcome set (COS) specifies the minimum group of outcomes considered essential for assessment and reporting in clinical trials addressing a particular health condition or patient population (Williamson et al., 2012). Its use does not prevent investigators from evaluating additional outcomes that are relevant to the objectives and design of an individual study. Establishing a COS can harmonize decisions regarding outcome selection, measurement, and disclosure, while decreasing the risk of selective reporting and reducing avoidable duplication and research waste (Williamson et al., 2017; Chalmers et al., 2014). Consistent use of these outcomes can also strengthen comparisons between studies and increase the interpretability and reliability of evidence generated through systematic reviews and meta-analyses (Kirkham et al., 2017). For systematic reviewers, an available COS may provide a useful reference for determining which outcomes should receive priority during data extraction, synthesis, and presentation (Saldanha et al., 2021). The development of a COS typically involves several successive stages, with an early systematic review commonly undertaken to compile the outcomes reported in previous clinical trials (Williamson et al., 2017).

No previous review, to the best of our knowledge, has comprehensively investigated how outcomes are selected and reported in randomized controlled trials (RCTs) evaluating traditional Chinese medicine (TCM) interventions for chronic pharyngitis. Accordingly, the present review was undertaken to catalogue and categorize the outcomes used in eligible trials, as well as to examine their definitions, timing of assessment, and frequency of reporting. These findings are intended to inform the subsequent development of a core outcome set (COS) for this clinical area.

## 2 materials and methods

### 2.1 Methodological framework

The methodological approach for this scoping review was informed by the JBI guidance described by (Peters et al., 2021), which draws on the original framework developed by Arksey and O’Malley and the subsequent refinements proposed by (Levac et al., 2010). Five principal stages were undertaken: defining the review question, locating potentially relevant evidence, determining eligibility, charting the extracted information, and organizing and presenting the results. Consultation with stakeholders was not included at this stage, as perspectives from patients, healthcare professionals, and other key stakeholders are planned to be obtained during the forthcoming Delphi exercise and broader consensus process for COS development. Preparation of the review report followed the PRISMA Extension for Scoping Reviews (PRISMA-ScR).

### 2.2 Registration

A scoping review was conducted to map the extent and characteristics of the available evidence, catalogue relevant studies, and summarize current approaches to outcome measurement and reporting (Munn et al., 2018). According to the COMET Handbook, reviewing outcomes previously reported in relevant studies is commonly undertaken as an initial step in core outcome set (COS) development (Williamson et al., 2017). The COS development project was prospectively registered with the COMET Initiative (registration number 2601). The review was conducted in accordance with contemporary methodological guidance for scoping reviews (Alexander et al., 2024) and was reported following the PRISMA Extension for Scoping Reviews (PRISMA-ScR) (Tricco et al., 2018). Ethics approval was not required because the review relied exclusively on published randomized controlled trials and publicly available trial registry records and involved no recruitment of human participants or collection of primary individual-level data.

### 2.3 Search strategy

Eight bibliographic databases were systematically searched from inception through 30 August 2026. PubMed, Web of Science, Embase, and the Cochrane Library were used to retrieve English-language literature, whereas Chinese-language studies were identified through China National Knowledge Infrastructure (CNKI), Wanfang Data, the VIP Database, and SinoMed. For each database, the search strategy incorporated both controlled vocabulary and free-text keywords covering three principal concepts: chronic pharyngitis, traditional Chinese medicine, and randomized controlled trials. Database-specific subject headings, such as MeSH terms in PubMed and Emtree terms in Embase, were applied where appropriate, and the search components were linked using Boolean logic. The full strategy developed for PubMed is provided in Supplementary Table SX1 and was subsequently modified to accommodate the indexing conventions and search functions of the remaining databases.

A complementary search of ClinicalTrials.gov, the Chinese Clinical Trial Registry (ChiCTR), and the World Health Organization International Clinical Trials Registry Platform (WHO ICTRP) was also performed to locate relevant trial registrations. This search enabled the identification of outcomes documented in registry records before the corresponding study findings were published. When a registry entry could be linked to a peer-reviewed article, the registered outcomes were compared with those subsequently reported in the publication.

### 2.4 Study selection

Microsoft Excel was used to compile the retrieved records and manage the study-selection process. Two reviewers (Pang and Li) independently screened the titles and abstracts of all records. Any record for which eligibility could not be determined from the available information was retained for full-text assessment. The full texts of potentially eligible reports were subsequently evaluated against the prespecified inclusion and exclusion criteria. Disagreements at either stage were resolved through discussion between the two reviewers; when consensus could not be reached, a third reviewer (Zhang) was consulted to arbitrate.

### 2.5 Inclusion and exclusion criteria

Studies were included if they met the following criteria:

- Only randomized controlled trials were eligible for inclusion.
- Participants diagnosed with chronic pharyngitis.
- Studies evaluating traditional Chinese medicine (TCM) interventions for chronic pharyngitis, restricted to orally administered or other pharmacological therapies.
- TCM interventions administered either as monotherapy or in combination with conventional treatment, provided that the therapeutic contribution of the TCM component could be clearly identified.
- Comparators comprising placebo, no treatment, usual care, conventional medicine, another TCM intervention, or other active treatments.
- Trials reporting at least one outcome relevant to chronic pharyngitis, including symptoms or signs, physical or laboratory examinations, TCM syndrome-related outcomes, health-related quality of life, safety outcomes, long-term prognosis, economic outcomes, or other clinically relevant endpoints.
- For multiple reports based on the same or substantially overlapping participant sample, the most recent publication or the report containing the most comprehensive outcome information was retained.
- Availability of the full-text publication in either English or Chinese.

Studies were excluded if they met any of the following criteria:

- Studies that did not employ a randomized controlled trial design.
- Studies that did not specifically investigate chronic pharyngitis, or studies enrolling participants with mixed pharyngeal or upper respiratory tract disorders when data for chronic pharyngitis could not be extracted separately.
- Studies were excluded when pharyngeal symptoms were secondary to an identifiable cause or another specific disorder rather than common chronic pharyngitis. Examples included gastroesophageal or laryngopharyngeal reflux, allergic conditions, postnasal drip, chronic tonsillitis, laryngitis, malignancy, treatment-related pharyngeal injury, and other defined local or systemic diseases.
- Studies evaluating non-pharmacological traditional Chinese medicine interventions, such as acupuncture, moxibustion, cupping, massage, acupoint application, or other forms of acupoint stimulation.
- Studies in which the TCM intervention was combined with other therapies but the TCM component could not be clearly identified or its contribution could not be distinguished between the intervention and comparator groups.
- Studies focusing primarily on disease prevention, rehabilitation, etiological or mechanistic investigation, pharmacological experiments, animal studies, or *in vitro* research rather than the clinical treatment of chronic pharyngitis.
- Studies enrolling participants with serious concomitant throat disorders, acute infectious diseases, or other conditions likely to substantially confound the evaluation of chronic pharyngitis outcomes.
- Studies that did not report relevant outcomes or for which the outcome data were unavailable, incomplete, or could not be reliably extracted.
- Publications for which the full text was not available in either English or Chinese.

### 2.6 Data extraction

A data-charting form was created in Microsoft Excel based on the recommendations of the COMET Handbook (Williamson et al., 2017). Li and Pang pilot-tested the form on four eligible reports and revised it after resolving discrepancies. Both reviewers then independently charted study and publication characteristics, participant eligibility, interventions and comparators, sample sizes, primary and secondary outcomes, outcome definitions, assessment time points, funding, and conflicts of interest. Outcome terms were retained verbatim from the original reports. For registry records without an initially identified publication, the eight databases were searched using the registered title and investigators’ names. Data from all eligible reports associated with the same study were charted, and disagreements were resolved through discussion.

### 2.7 Data standardization

Outcome terms were standardized with reference to established international and Chinese medical terminologies, including SNOMED CT, MeSH, relevant clinical guidelines, the Chinese Traditional Medicine Subject Headings, and the national standard Clinical Terminology of Traditional Chinese Medicine Diagnosis and Treatment. For outcomes specific to traditional Chinese medicine, national and professional standards were prioritized to minimize inconsistencies arising from non-standard expressions. Each term was reviewed according to its wording and intended meaning, with the original clinical concept retained wherever possible. The harmonization procedures involved correcting evident errors, consolidating synonymous or alternative terms, unifying English abbreviations, separating or combining terms when necessary, clarifying ambiguous references, and assigning a common standardized label to outcomes representing the same concept.

Specifically, terms were merged only when they clearly referred to the same underlying clinical concept, based on their wording, intended meaning, context in the original report, and relevant terminology sources. Spelling variants, abbreviations and their full forms, and synonymous or alternative expressions were standardized to a common label, whereas terms representing clinically distinct symptoms, signs, or constructs were retained separately. Composite descriptors were separated when their constituent outcomes could be clearly distinguished; when equivalence remained uncertain, the terms were not merged until consensus was reached.

Two reviewers independently performed the initial standardization and classification of outcome terms according to the predefined rules. Disagreements were resolved through discussion and consensus, with any unresolved discrepancies referred to a third reviewer for adjudication. Inter-rater agreement was assessed using Cohen’s kappa coefficient based on the reviewers’ initial independent classifications before consensus was reached. The reviewers achieved an overall agreement rate of 95.2%, with a Cohen’s κ of 0.81, indicating a high level of agreement.

### 2.8 Data analysis

A narrative synthesis was undertaken to summarize the design characteristics of the included studies, the outcomes reported, and the time points at which outcomes were assessed. The COMET taxonomy was used as the initial framework for organizing the extracted outcomes (Williamson et al., 2017), thereby facilitating a systematic and transparent assessment of outcome-reporting practices and limiting the potential impact of inconsistent or selective reporting on subsequent evidence synthesis (Kirkham et al., 2010). To account for the specific theoretical and clinical characteristics of traditional Chinese medicine, the outcomes were subsequently classified using the seven-domain TCM framework proposed by Professor Zhang Junhua and colleagues (Zhang et al., 2021). During this process, TCM-specific outcomes were first aligned with the 12 outcome categories recommended by COMET and were then grouped according to their functional attributes into the following seven domains: TCM disease and syndrome, symptoms/signs, physical and chemical examinations, quality of life, long-term prognosis, economic evaluation, and safety events (Williamson et al., 2017; Zhang et al., 2021). The cross-mapping of standardized outcome terms between the COMET 12-category framework and the seven-domain TCM outcome classification is presented in Supplementary Table SX2.

During outcome conceptualization, two researchers jointly undertook the initial processing of the extracted indicators. Pang was responsible for preliminary extraction and domain assignment, whereas Li independently reviewed the results and consolidated indicators that represented equivalent or closely related concepts. The proposed domains and their definitions were subsequently examined by a multidisciplinary steering group comprising COS methodologists and senior experts from relevant fields. Revisions were made through iterative consensus discussions until agreement was reached (Kirkham et al., 2017). Descriptive statistics were used to summarize the reporting frequency of individual indicators. Associations and co-reporting patterns among indicators were further explored using phi correlation coefficients and hierarchical clustering heatmaps.

The present review focused on identifying, standardizing, and classifying reported outcomes and their assessment time points, rather than on the outcome measurement instruments used to assess them. Accordingly, measurement instruments were not systematically evaluated, and their measurement properties, such as reliability, validity, and responsiveness, were not formally appraised. The identification and evaluation of appropriate measurement instruments represent a subsequent stage of core outcome set development and were beyond the scope of the present review (Prinsen et al., 2016; Gorst et al., 2020). Although the COSMIN methodology provides established guidance for evaluating patient-reported outcome measures (Mokkink et al., 2018), a broadly endorsed framework applicable to the full range of TCM-specific measures remains lacking, especially for indicators related to TCM syndromes (Li et al., 2022).

## 3 Results

### 3.1 Search results and study selection

The database searches yielded 8,925 records. After 2,540 duplicate entries were removed, 6,385 unique records remained for title and abstract screening. This step eliminated 4,248 studies, prompting a detailed full-text examination of the 2,137 candidate publications.

Of these, 1,817 reports were deemed ineligible. Exclusion criteria primarily comprised inappropriate patient populations (n = 850), disqualified interventions (n = 366), and inadequate study designs (n = 578). Minor reasons included missing/unextractable outcome metrics (n = 10), overlapping data sets (n = 10), and inaccessible full-text sources (n = 3). Specifically, population exclusions were mostly driven by combined respiratory/pharyngeal conditions without distinct subgroup data for pharyngitis. For interventions, studies were screened out primarily due to the use of external rather than oral TCM regimens. In terms of study design, the majority of exclusions were attributed to animal experiments or non-randomized methodologies.

Following full-text assessment, 320 trials fulfilled the eligibility criteria and were included in the review. Figure 1 presents the study-selection pathway using a PRISMA flow diagram.

**FIGURE 1:**
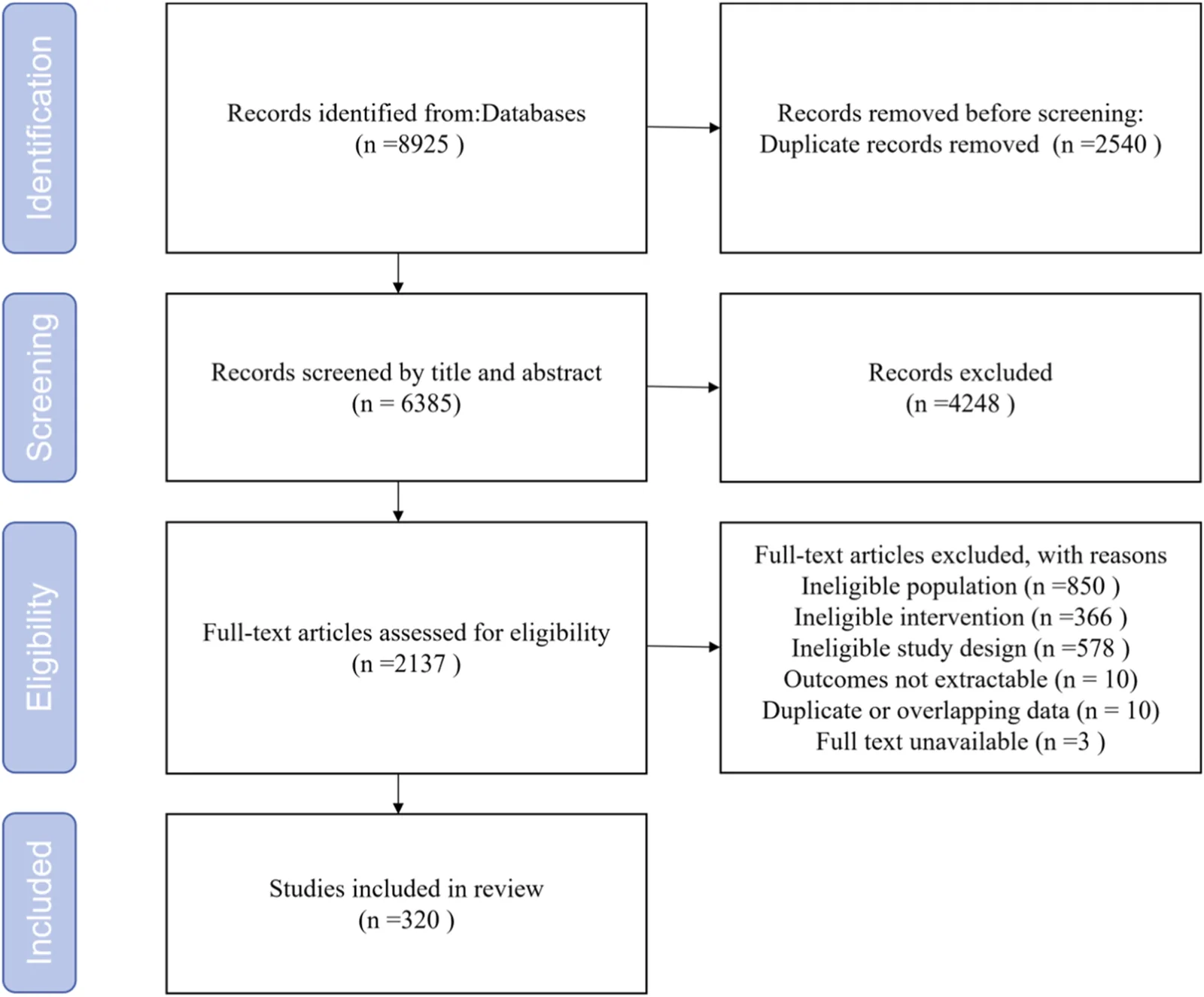
PRISMA diagram of study selection for inclusion into the review.

### 3.2 Study characteristics

The final scoping review comprised 320 studies encompassing 48,536 participants. Sample sizes per study varied extensively from 20 to 1,004, with a calculated median of 106 subjects (IQR, 80–163).

The application frequencies of the top ten TCM formulations are illustrated in Figure 2. Nourishing Yin Clearing Lung Soup ranked highest in reporting frequency (n = 9, 2.8%), followed by Lanqin oral solution and Jinsang Liyan Pills (n = 8, 2.5% each), and Pinellia Houpu Soup (n = 7, 2.2%). An additional six interventions (Maintain Yin Relieving Sore-Throat Decoction, Yiqing Capsule, Yangyin Liyan Decoction, Qianyang Fengsui Dan, Houyanqing Granules, and Andrographolide Dropping Pills) were reported in four papers each (1.2%). The observation that even the most frequently cited intervention appeared in less than 3% of the literature highlights substantial heterogeneity and a lack of standardized treatment approaches across the reviewed studies.

**FIGURE 2:**
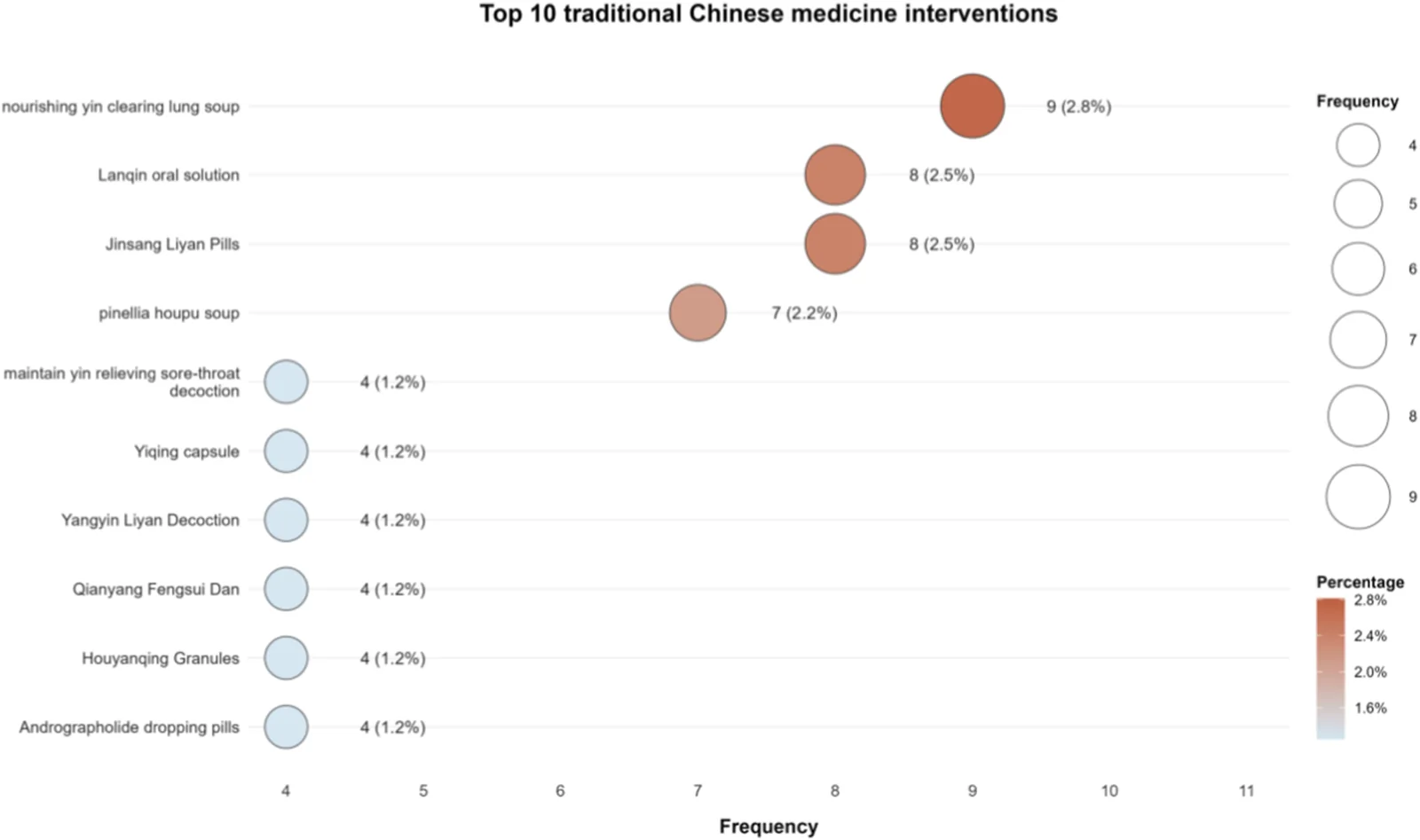
Top 10 most frequently reported traditional Chinese medicine interventions.

To provide a more comprehensive characterization of these interventions, the composition and available preparation-related information for the identified TCM formulations are presented in Supplementary Appendix A1. The medicinal constituents were further standardized taxonomically and pharmacopoeially: vascular botanical drugs, including their accepted scientific names, botanical authorities, families, medicinal parts, and pharmacopoeial drug names where available, are reported in Supplementary Appendix A2.1, whereas fungal, algal, animal-derived, mineral, fermented, purified, and other medicinal constituents are presented in Supplementary Appendix A2.2. In addition, the reporting completeness of the commonly investigated TCM preparations was assessed according to the ConPhyMP recommendations, with the detailed assessment provided in the Supplementary Material.

The therapeutic duration varied considerably across studies, ranging from 5 to 90 days (Figure 3). A 14-day regimen was the most common (n = 97, 30.3%), followed by 30 days (10.9%), 28 days (10.0%), and 21 days (8.8%). Other durations, such as 10, 7, and 20 days, were utilized in 7.2%, 6.9%, and 5.3% of the studies, respectively. Meanwhile, 15-, 60-, and 5-day treatment periods were relatively uncommon (3.1%–3.8%). Aside from six studies (1.9%) that did not specify a timeframe, all other distinct durations each accounted for less than 1.9%. Overall, despite the broad range of intervention timelines, the vast majority of treatment courses clustered within the 7- to 30-day window.

**FIGURE 3:**
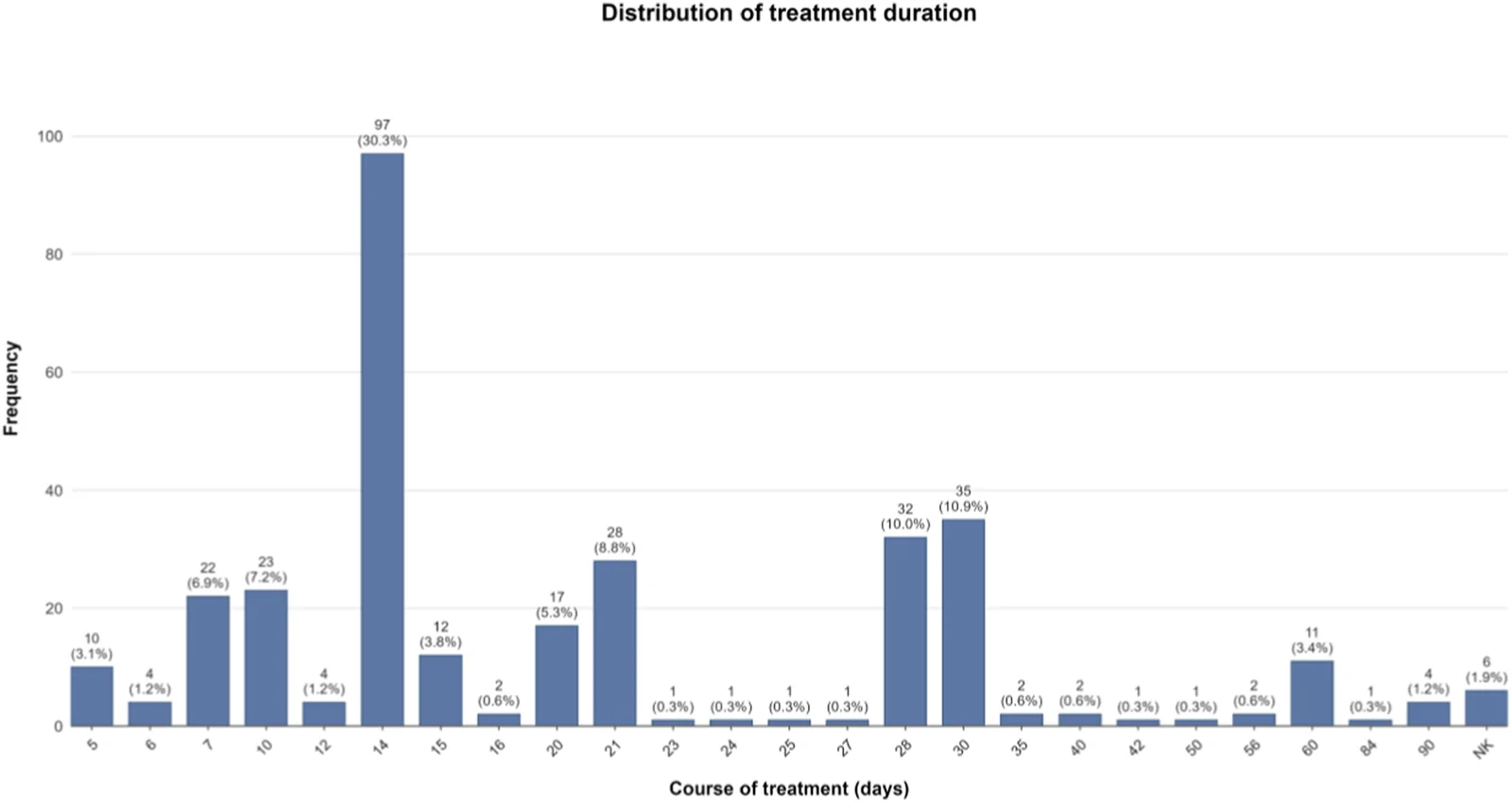
Distribution of treatment duration across the included studies.

### 3.3 Risk-of-bias assessment

Application of the RoB two tool to the 320 included randomized controlled trials indicated that most studies were rated as having “some concerns” overall, while none received an overall high-risk judgement. Concerns arose primarily from the randomization process, outcome measurement, and selection of the reported result. In the randomization domain, only 35.3% of trials were classified as low risk, largely because methods for sequence generation and allocation concealment were inadequately described. By comparison, the domains concerning deviations from intended interventions and missing outcome data were generally well addressed, with almost all trials judged to be at low risk. Measurement of the outcome represented another important source of concern, with only 14.0% of studies receiving a low-risk judgement. This was largely attributable to the frequent use of subjective or patient-reported symptom outcomes and inadequate reporting of blinding of participants or outcome assessors where such blinding was relevant. Concerns regarding selection of the reported result were also common. Only two studies had accessible trial registrations, while protocols or prespecified analysis plans were unavailable for the remaining studies; consequently, the possibility that reported results were selected from among multiple eligible outcome measurements or analyses could not be adequately excluded.

### 3.4 Outcome domains

Using the traditional Chinese medicine classification framework proposed by Zhang et al. (2021), we identified 1,204 outcomes across the 320 included studies and categorized them into seven overarching domains (Table 1). In this review, an individual outcome was defined as each reported instance of an outcome within a single trial; therefore, the total count reflects outcome occurrences across studies rather than unique outcome concepts after semantic deduplication. The trial registry search identified two eligible registration records. For both registered trials, all prespecified outcomes listed in the registration records were also reported in the corresponding publications, and no additional outcomes were identified from the registry records. Consequently, the candidate outcome list remained unchanged at 1,204 outcomes.

**TABLE 1:** Outcome domains.

Outcome domain	Outcome	Frequency
Symptoms/Signs	Clinical efficacy	299
	Pharyngalgia	71
	Foreign body sensation	64
	Dry throat	50
	Dry cough	45
	Itchy throat	45
	Mucosal hyperemia	32
	Posterior pharyngeal wall lymphoid follicular hyperplasia	24
	Pharyngeal symptoms	22
	Burning sensation	21
	Dry and itchy throat	17
	Pharyngeal signs	14
	Secretions	13
	Symptom and sign score	12
	Hoarseness	9
	Sensation of sticky sputum	9
	Expectoration	8
	Mucosal dryness	6
	Mucosal edema	5
	Thickening of the pharyngeal mucosa	5
	Hyperemia of the posterior pharyngeal wall	4
	Hypertrophy of the lateral pharyngeal bands	4
	Morning retching	4
	Aggravation after excessive talking	3
	Dry and sore throat	3
	Main symptoms	3
	Secondary symptoms	3
	Symptoms and signs	3
	Vasodilation of the posterior pharyngeal wall	3
	Burning sensation / Foreign body sensation	2
	Changes in the pharyngeal mucosa	2
	Cough with excessive sputum	2
	Mucosal hyperemia and edema	2
	Pharyngeal redness and swelling	2
	Redness and swelling of the lateral pharyngeal bands	2
	Sore throat aggravated by excessive talking	2
	Sore throat with bleeding	2
	Abnormal taste	1
	Cure rate	1
	Dry cough with sputum	1
	Dry mouth	1
	Dry stool	1
	Dry throat with burning sensation	1
	Dry throat with reduced fluid/moisture	1
	Dysphagia	1
	Halitosis	1
	Hyperemia and hypertrophy of the lateral pharyngeal bands	1
	Itchy throat with dry cough	1
	Lymphoid follicular hyperplasia at the base of the tongue	1
	Pharyngeal mucosal hyperemia and edema with lymphoid follicular hyperplasia	1
	Pseudomembrane shedding	1
	Throat clearing	1
	Time to cure	1
	Tonsillar hypertrophy	1
	Yellow urine	1
Physical and chemical examination	TNF-α	38
	IL-6	29
	CD4+	12
	CRP	12
	IL-2	12
	CD4+/CD8+	11
	CD8+	11
	CD3+	10
	hs-CRP	6
	IL-1β	5
	MMP-9	4
	NEUT	4
	SIgA	4
	VCAM-1	4
	EOS	3
	IgA	3
	EGF	2
	IL-1	2
	LY	2
	ALT	1
	AST	1
	B cells	1
	BUN	1
	Dysbiosis	1
	hepatic and renal function	1
	IgE	1
	IgG	1
	IgM	1
	IL-8	1
	MO	1
	PCT	1
	PGE2	1
	Positive expression rate of PCNA in pharyngeal lesion tissues	1
	Routine blood and urine examination	1
	Scr	1
	WBC	1
Security event	Incidence of adverse reactions	69
	Incidence of side effects	3
	Incidence of complications	2
	Safety assessment	1
	Toxic side effects	1
TCM Syndrome	Efficacy on TCM syndrome	12
	Efficacy on individual TCM syndrome items	6
	Individual TCM syndrome item score	5
	Abnormal tongue manifestation	3
	TCM syndrome score	3
	Abnormal pulse manifestation	2
	Individual TCM symptom score	2
	TCM symptom score	2
Long-term prognosis	Recurrence rate	32
	Prognosis	1
Quality of life	Bodily Pain (SF-36-BP)	2
	General Health (SF-36-GH)	2
	Mental Health (SF-36-MH)	2
	Patient Satisfaction	2
	Physical Functioning (SF-36-PF)	2
	Role-Emotional (SF-36-RE)	2
	Role-Physical (SF-36-RP)	2
	Self-Rating Anxiety Scale (SAS)	2
	Self-Rating Depression Scale (SDS)	2
	Social Functioning (SF-36-SF)	2
	Vitality (SF-36-VT)	2
	Material Life Status (GQOL-74-MLS)	1
	Medication Adherence	1
	Physical Function (GQOL-74-PF)	1
	Psychological Function (GQOL-74-PsF)	1
	Satisfaction Rate (SR)	1
	SF-36 score	1
	Social Function (GQOL-74-SF)	1
Economic evaluation	Cost-effectiveness ratio (CER)	2
	Drug cost (C)	1
	Effectiveness (E)	1
	Incremental cost-effectiveness ratio (ICER)	1

Outcome reporting was heavily concentrated in the Symptoms/Signs domain, which comprised 835 outcome records and accounted for 69.3% of all extracted outcomes. At least one symptom- or sign-related outcome was identified in every study included in the dataset, underscoring the predominance of clinical symptom assessment in chronic pharyngitis research. Physical and chemical examination ranked second, with 191 records (15.9%), followed by Safety event, with 76 records (6.3%). Considerably smaller proportions were observed for TCM Syndrome (n = 35, 2.9%), long-term prognosis (n = 33, 2.7%), and Quality of life (n = 29, 2.4%). Economic evaluation was the least represented domain, contributing only five records (0.4%) (Table 1). Across the included studies, the average number of distinct outcome domains reported per study was 1.63, with values ranging from one to 5; the median was one and the interquartile range was 1–2. The greatest domain coverage was observed in two studies, each of which included outcomes from five domains.

Figure 4 illustrates the distribution of reported outcomes across the seven predefined domains using a Sankey diagram. Outcome reporting was dominated by the Symptoms/Signs domain (835 records, 69.3%). Within this domain, clinical efficacy was the most frequently reported outcome (n = 299, 25.0%), followed by primary localized manifestations such as pharyngalgia (n = 71, 5.9%) and foreign body sensation (n = 64, 5.3%). Other specific manifestations, including dry throat, cough, and mucosal hyperemia, contributed to a long tail of varied terminologies. The Physical and chemical examination domain ranked second (191 records, 15.9%), predominantly featuring inflammatory biomarkers such as TNF-α (n = 38, 3.2%) and IL-6 (n = 29, 2.4%), alongside immune indicators like CD4^+^ and CRP. The Safety event domain (76 records, 6.3%) was overwhelmingly represented by the incidence of adverse reactions (n = 69, 5.8%), with specific complications rarely detailed. The remaining four domains accounted for a minimal proportion of outcomes. In the TCM Syndrome domain, overall efficacy on TCM Syndrome was the leading metric. Long-term prognosis outcomes (33 records) consisted almost entirely of recurrence rate (n = 32, 2.7%). Quality of life (29 records, 2.4%) occasionally utilized tools like the SF-36, SAS, and SDS. Finally, economic evaluation was the least represented domain (5 records, 0.4%), primarily reporting cost-effectiveness ratios. Overall, outcome reporting in chronic pharyngitis trials was heavily skewed toward short-term clinical efficacy and localized manifestations, leaving quality of life, economic consequences, and long-term prognosis largely underexplored.

**FIGURE 4:**
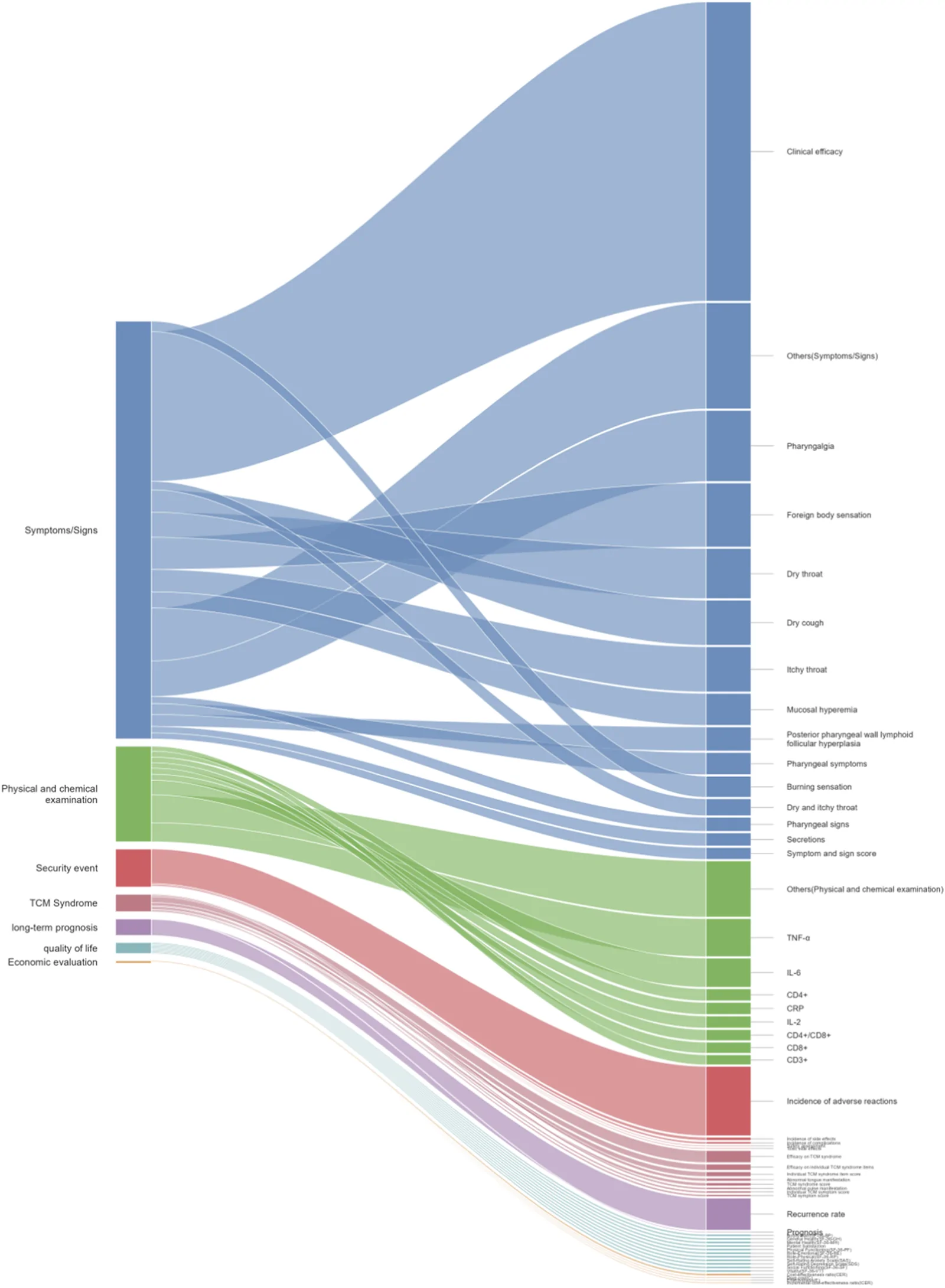
Sankey diagram illustrating the distribution of outcomes across the seven predefined domains.

An analysis of temporal trends demonstrated a gradual diversification in the selection of outcome domains across the analyzed time periods (Figure 5). Although Symptoms/Signs remained the most frequently reported domain—peaking between 2005 and 2009—its dominance noticeably waned from 2010 onward. Conversely, the utilization of Physical and chemical examination surged markedly, especially post-2015, reflecting a growing reliance on objective clinical data. TCM Syndrome assessments constituted a notable proportion prior to 2000 but plateaued as a minor, stable component in subsequent years. Notably, Safety event reporting emerged in the early 2000s and became more prominent during the 2010–2014 period, yet its overall prevalence remained modest. Indicators for long-term prognosis were sparsely documented throughout, and Economic evaluation was rarely observed. The introduction of Quality of life metrics in the 2015–2019 interval, along with their expansion in the most recent period, highlights an emerging paradigm shift toward patient-centered evaluations. Ultimately, the data reveal a transition from a predominantly Symptoms/Signs-driven approach to a more comprehensive evaluation framework that integrates objective laboratory findings, Safety event profiles, and patient wellbeing.

**FIGURE 5:**
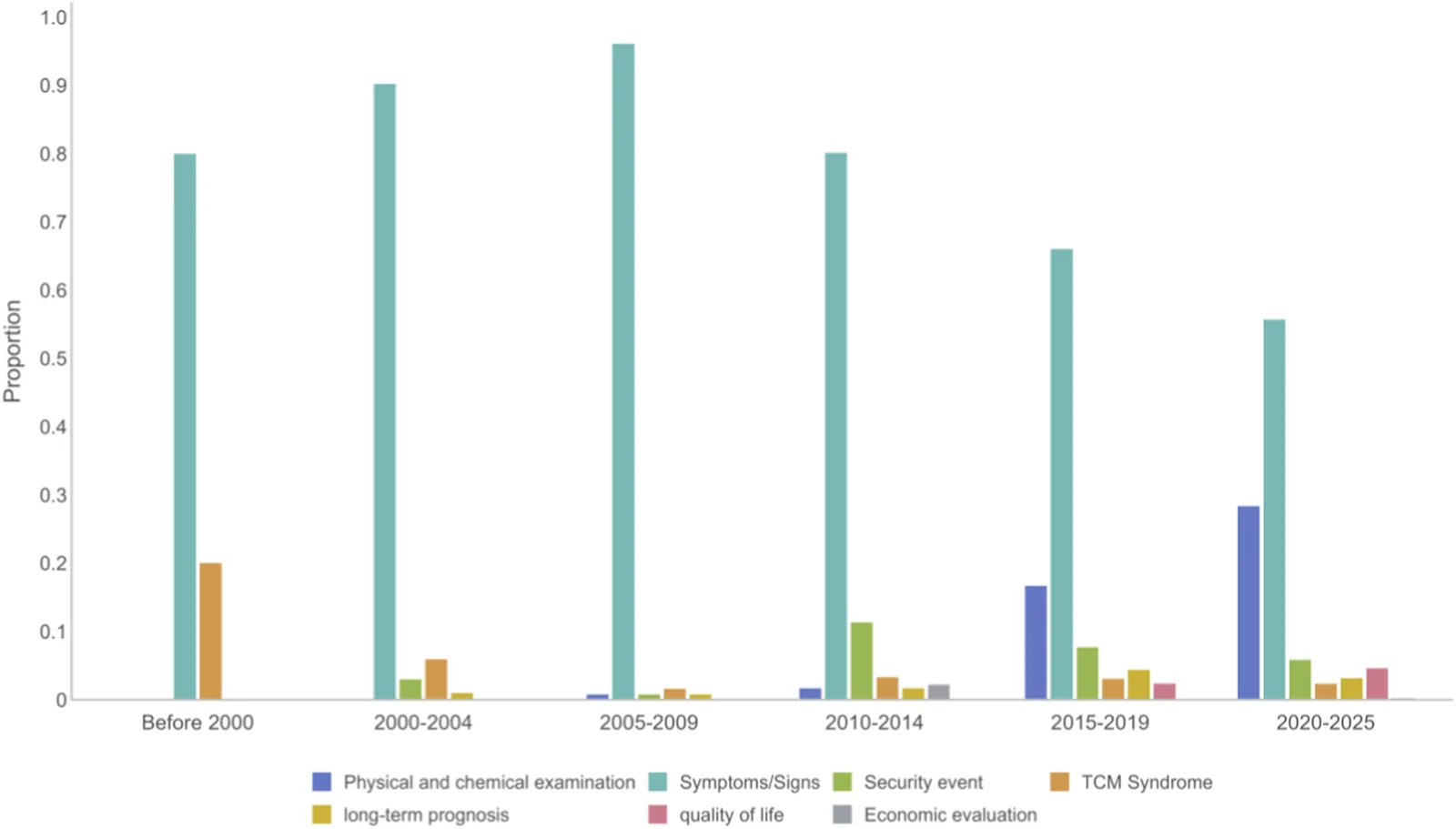
Temporal trends in the distribution of outcome domains across included studies.

### 3.5 Outcome

Among the individual outcomes, clinical efficacy was reported far more frequently than any other measure, accounting for nearly one-quarter of all outcome occurrences (n = 299, 24.8%) (Figure 6), but its operational definition varied considerably across trials. Typical approaches included categorical assessment based on improvement or resolution of symptoms and pharyngeal signs, quantitative classifications based on percentage reductions in symptom/sign scores, criteria incorporating recurrence during follow-up, and composite definitions combining clinical manifestations with objective or laboratory indicators. Numerical thresholds also differed across reference standards. Detailed examples of these operational definitions are provided in Supplementary Table SX3. The remaining frequently reported outcomes were largely symptom- or sign-related, including pharyngalgia, foreign body sensation, dry throat, itchy throat, dry cough, and mucosal hyperemia. However, no single symptom outcome approached the frequency of clinical efficacy, reflecting considerable fragmentation in the selection and terminology of symptom-based assessments. Safety reporting was represented mainly by the incidence of adverse reactions (n = 69, 5.7%), whereas recurrence rate was the only relatively prominent outcome addressing longer-term treatment effects. Objective assessments were less frequently reported and concentrated on a limited range of inflammatory and immune markers, particularly TNF-α and IL-6. TCM-specific evaluation was also uncommon, with efficacy based on TCM syndrome reported only 12 times (1.0%). Overall, the distribution was characterized by a strong reliance on general clinical efficacy, accompanied by dispersed symptom measures and comparatively limited use of standardized objective, prognostic, and TCM-specific outcomes.

**FIGURE 6:**
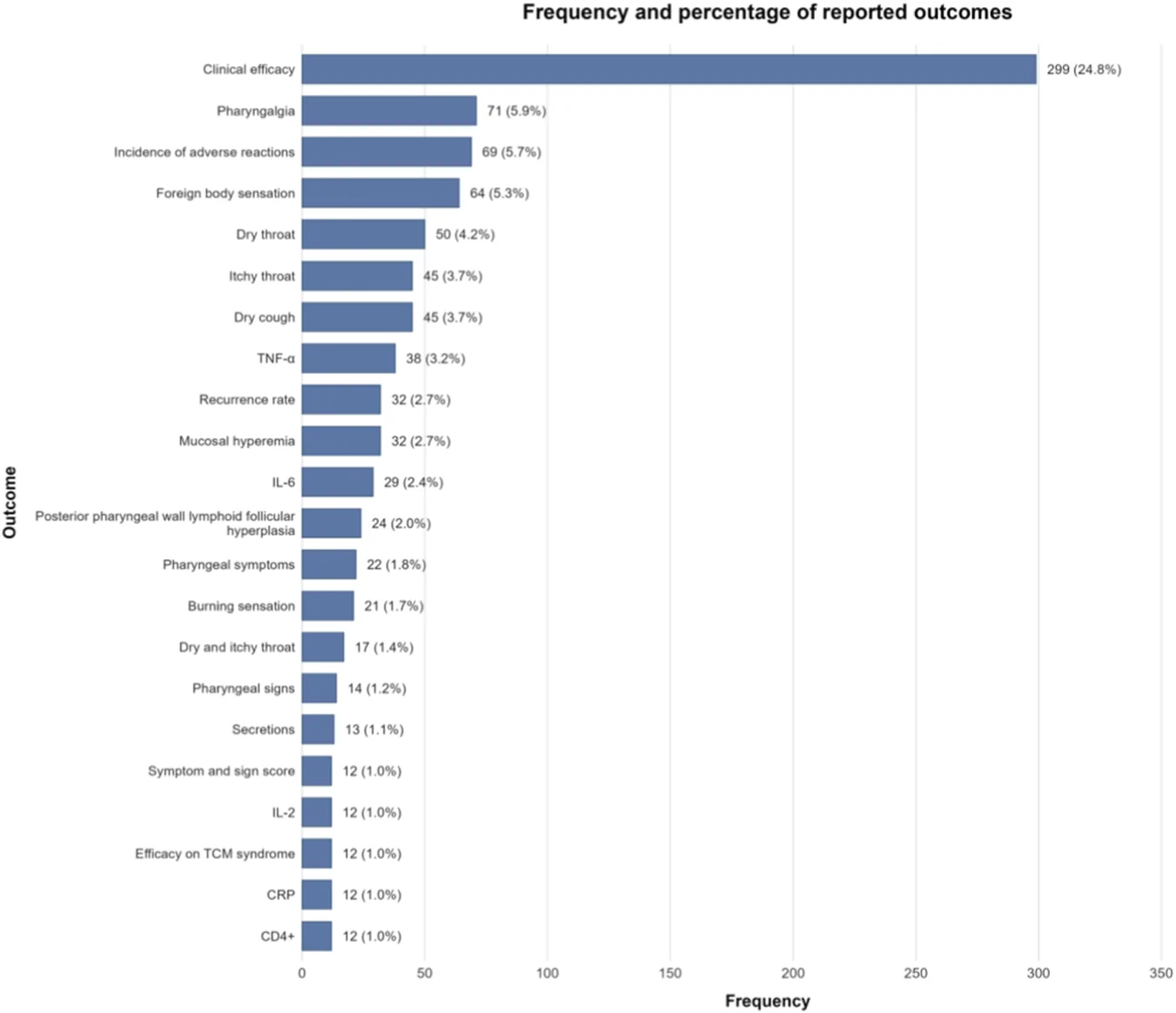
Bar chart of the most frequently reported outcome measures across included studies.

Hierarchical clustering of Phi coefficients (k = 5) revealed distinct patterns of outcome co-occurrence (Figure 7). The first major symptom- and sign-related cluster encompassed throat pain, foreign body sensation, dryness, itching, cough, and local pharyngeal findings, indicating that these manifestations were commonly evaluated together. Within this cluster, particularly strong associations were observed between pharyngalgia and foreign body sensation (φ = 0.78), and between dry throat and both pharyngalgia and foreign body sensation (φ = 0.73 for each). A second major cluster was predominantly comprised of inflammatory and immunological indicators. The strongest relationships occurred among CD3^+^, CD4^+^, CD8^+^, and the CD4+/CD8+ ratio (φ = 0.85–0.96), while IL-6 and TNF-α were also closely associated (φ = 0.76), demonstrating consistent combined assessment of related biomarkers. In contrast, the remaining three smaller clusters highlighted the isolation of specific assessment domains. Clinical efficacy formed an isolated cluster and showed weak or negative associations with several specific symptom measures, suggesting it was often reported as a global treatment endpoint rather than being strictly tied to detailed symptom assessments. Similarly, TCM syndrome efficacy measures and safety-related outcomes (e.g., adverse reactions) were located outside the two principal clusters, generally exhibiting limited associations with other outcomes. Overall, the clustering pattern indicates that outcome selection was primarily organized around symptom-based evaluation and biomarker assessment, whereas global efficacy, safety, and TCM-specific measures were comparatively disconnected from these multidimensional assessment structures.

**FIGURE 7:**
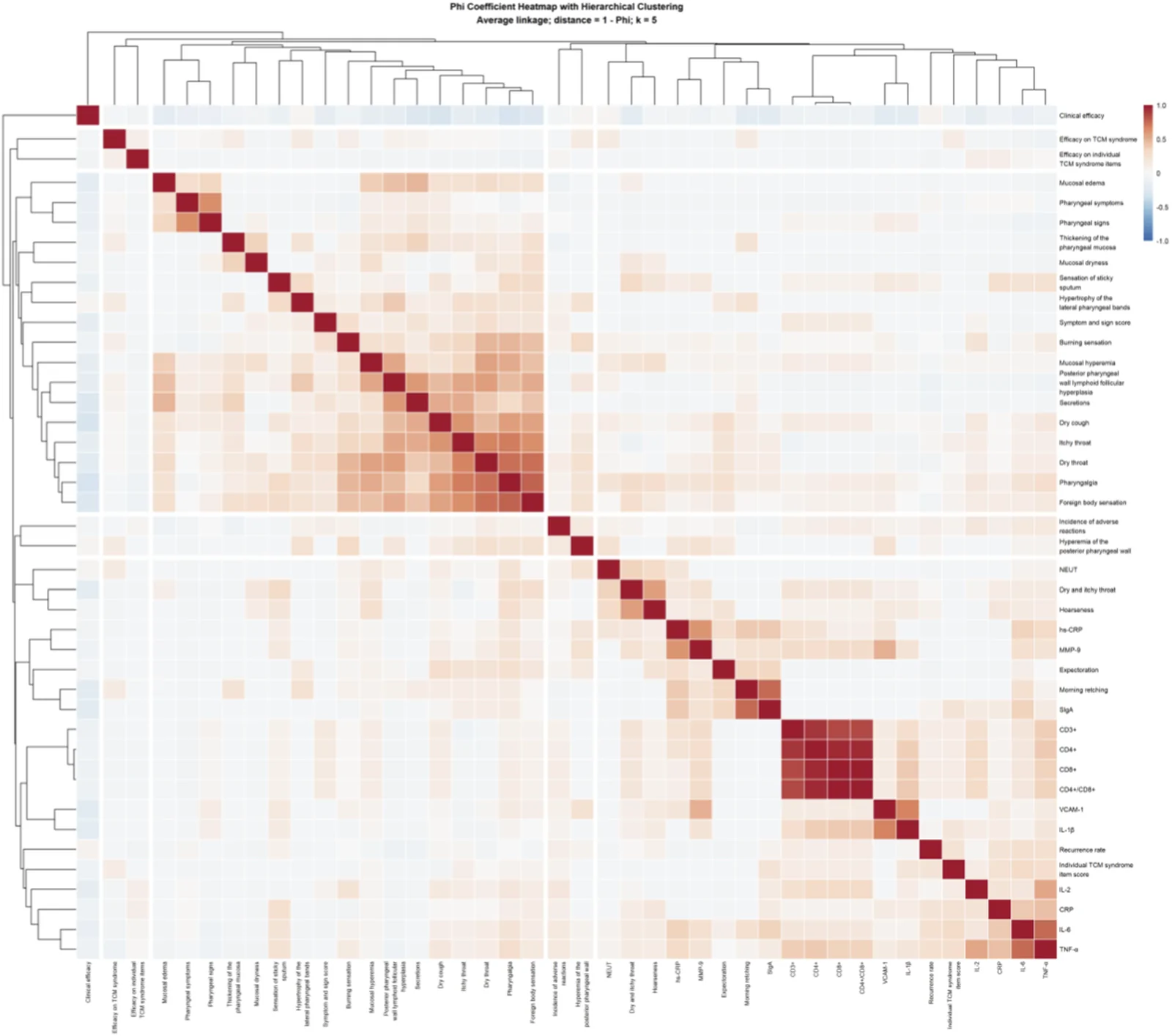
Phi coefficient matrix with hierarchical clustering of outcome measures.

### 3.6 Measurement time point

Measurement schedules for the 20 most frequently reported outcomes varied substantially, although assessments were predominantly concentrated within the first 30 days (Figure 8). Day 14 was the most common or joint-most common assessment point for 16 of the 20 outcomes, including clinical efficacy (87/298, 29.2%), pharyngalgia (25/71, 35.2%), and foreign body sensation (24/63, 38.1%). This concentration was particularly pronounced for laboratory indicators, with 83.3% of IL-2 assessments, 58.3% of CD4^+^ assessments, 50.0% of TNF-α assessments, and 48.3% of IL-6 assessments conducted on day 14. Days 21, 28, and 30 constituted the other principal assessment points. Clinical efficacy exhibited the greatest temporal variability, with specified measurements ranging from day 3 to day 201, indicating limited standardization of assessment schedules across studies. In contrast, recurrence was evaluated at more dispersed and substantially later time points, most frequently on day 194 (8/32, 25.0%) and extending to day 385. Notably, the measurement time point was unspecified in all 69 studies reporting the incidence of adverse reactions, revealing a considerable deficiency in the temporal reporting of safety outcomes.

**FIGURE 8:**
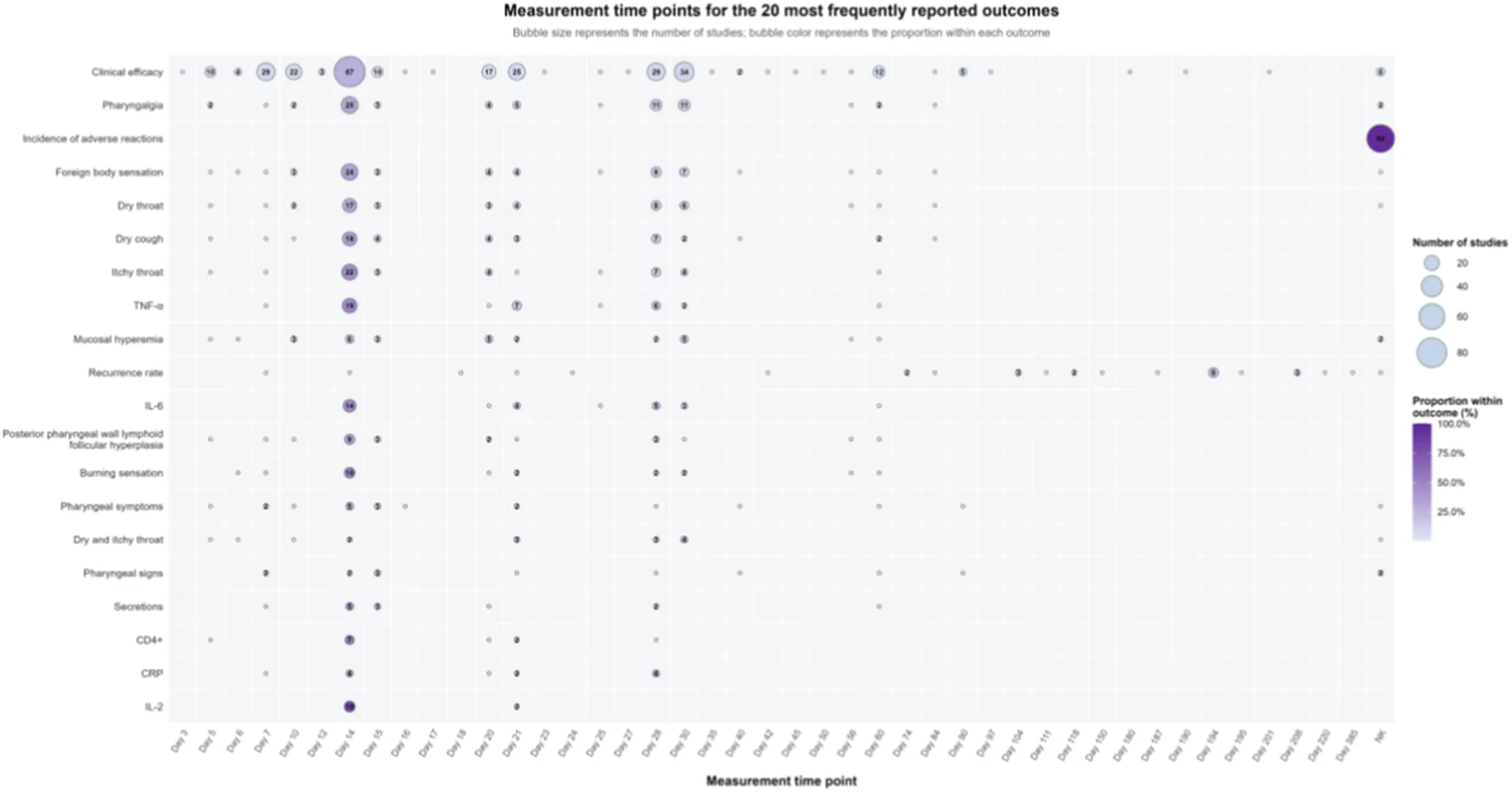
Distribution of measurement time points for the 20 most frequently reported outcomes.

A cross-analysis of treatment duration and outcome assessment timing showed that assessments were predominantly conducted at treatment completion. Among the 313 trials with at least one clearly specified assessment time point, 308 (98.4%) included an assessment at the end of the treatment period, while 270 (86.3%) reported no other clearly specified assessment time point. Only 34 trials (10.9%) included at least one post-treatment assessment. This pattern was particularly evident among trials with a 14-day treatment course: all 93 trials included an assessment at day 14, whereas only 16 (17.2%) included an additional post-treatment assessment. Detailed results of the cross-analysis according to treatment duration are presented in Supplementary Table SX4.

## 4 Discussion

Considerable variation was observed in the outcomes selected and reported across studies evaluating traditional Chinese medicine interventions for chronic pharyngitis. No trial assessed all seven predefined outcome domains, although it may be neither practical nor necessary for a single study to encompass the full range of domains identified in this review. The maximum number of domains evaluated within an individual study was five, and this level of coverage occurred in only two studies. Almost all studies assessed at least one Symptoms/Signs outcome, with this domain appearing in 318 studies (99.4%). By comparison, each of the remaining domains was reported in fewer than 25% of the included studies. A similarly uneven pattern was evident for individual outcomes. Clinical efficacy was evaluated in 298 studies (93.1%) and was the only specific outcome reported by more than one-third of the studies. Other relatively common outcomes, such as pharyngalgia, adverse reaction incidence, and foreign body sensation, were each included in less than one-quarter of the evidence base. These findings demonstrate a pronounced lack of consistency in outcome selection and support the need to establish a standardized set of clinically relevant outcomes for chronic pharyngitis trials, which would enhance comparability between studies and strengthen subsequent evidence synthesis.

The predominance of the Symptoms/Signs domain was not unexpected, given that chronic pharyngitis is chiefly characterized by persistent throat-related complaints, including pharyngalgia, dryness, itching, cough, and foreign body sensation. The frequent use of clinical efficacy as an outcome may reflect its convenience, low cost, and compatibility with routine clinical assessment, as it commonly integrates changes in several pharyngeal symptoms and signs into an overall response category. Nevertheless, such composite assessments may conceal substantial variation in the symptoms included, scoring criteria, response thresholds, and evaluation methods applied across studies. More recent research has adopted broader and more structured approaches to outcome assessment. Wu et al., for example, evaluated pharyngeal symptoms and signs separately and also considered illness perception and treatment adherence in a randomized trial of botanical lozenges for chronic pharyngitis (Wu et al., 2024). Similarly, a multicentre trial involving patients with persistent throat symptoms incorporated a validated symptom index, health-related quality-of-life measures, and assessments of throat appearance (O'Hara et al., 2021). A recent comprehensive evaluation of Jinsang Liyan Pills/Capsules further considered effectiveness, safety, economy, suitability, accessibility, and characteristics of traditional Chinese medicine (Zhou et al., 2023). These developments indicate that future chronic pharyngitis trials should move beyond broadly defined clinical response categories and adopt clearly specified, validated, and patient-relevant outcomes. This approach is also consistent with the CONSORT-Outcomes 2022 recommendations, which emphasize transparent reporting of outcome definitions, measurement methods, aggregation procedures, and assessment time points (Butcher et al., 2022).

The collection and reporting of patient-reported symptom burden, functional impact, and health-related quality of life reflect the growing recognition that the evaluation of interventions for chronic pharyngitis should extend beyond clinician-observed pharyngeal signs and overall response rates (Tu et al., 2026). Recent research has identified patient-centered outcomes as an underexplored area in chronic pharyngitis and has highlighted the need for more standardized and clinically meaningful outcome frameworks (Tu et al., 2026). Evidence from the broader sore-throat literature further indicates that persistent symptoms and their adverse effects on daily activities and sleep are important reasons for patients to seek treatment (van der Velden et al., 2020). These findings support the incorporation of validated patient-reported symptom and quality-of-life measures alongside objective clinical examinations in future chronic pharyngitis trials, thereby enabling a more comprehensive assessment of whether treatment benefits are meaningful to patients.

By contrast, health-related quality-of-life outcomes were reported in only a small proportion of studies (n = 10, 3.1%). This is despite emerging evidence that the burden of chronic pharyngitis may extend beyond local pharyngeal symptoms. Anxiety has been identified in approximately one-fifth of adults with chronic pharyngitis, with a longer disease duration associated with greater anxiety severity, suggesting that the psychological consequences of persistent symptoms warrant greater consideration (Zha et al., 2023). Recent trial evidence has also indicated that illness perception and treatment adherence may improve alongside pharyngeal symptoms and signs, highlighting the potential importance of patients’ understanding of and perceived control over their condition (Wu et al., 2024). As revealed by our clustering analysis, the included studies predominantly emphasize symptom relief and biological changes, which may explain the limited assessment of these other outcomes. However, reliance on these dimensions alone may fail to establish whether an intervention produces meaningful improvements in patients’ daily functioning, emotional wellbeing, or ability to manage their condition.

Our results indicate that sleep-related outcomes warrant greater consideration in future research on chronic pharyngitis. Persistent throat pain, dryness, itching, foreign body sensation, and cough may interfere with sleep initiation or continuity, while insufficient or poor-quality sleep may increase pain sensitivity and contribute to emotional distress. Longitudinal evidence supports a potentially bidirectional relationship between pain and sleep disturbance (Yi et al., 2024), and recent multicenter research in patients with otolaryngological disorders has demonstrated close interactions among sleep quality, anxiety, and depression (Qi et al., 2025). Nevertheless, sleep was not evaluated as a distinct outcome in the included studies, and direct evidence concerning its role in chronic pharyngitis remains limited. Further research is therefore needed to determine whether sleep disturbance represents a consequence of persistent pharyngeal symptoms, a factor that amplifies symptom burden, or both. Sleep, psychological wellbeing, illness perception, adherence, and quality of life should not be evaluated in isolation from clinical symptoms and objective findings. Rather, these outcomes should be incorporated into a multidimensional assessment framework that connects biological changes and symptom improvement with benefits that are directly meaningful to patients. Such integration may also reduce the dependence on broadly defined clinical efficacy outcomes, which can obscure which specific aspects of health have improved.

TCM syndrome-related outcomes were reported in a relatively small proportion of studies (n = 27, 8.4%). This may partly reflect the difficulty of translating syndrome differentiation, which may change over the course of treatment, into standardized outcomes that can be consistently defined, measured, and compared across trials. However, difficulties in standardization are unlikely to be the only explanation. Some trials may have evaluated fixed TCM interventions without explicitly incorporating syndrome differentiation into treatment selection, whereas others may have prioritized general clinical efficacy, symptoms, signs, or laboratory indicators over TCM syndrome outcomes. Thus, the limited reporting of TCM syndrome outcomes may reflect both challenges in standardizing syndrome assessment and differences in trial design and outcome prioritization. Because the use of syndrome-based treatment strategies and the prespecification of TCM syndrome outcomes as primary outcomes were not systematically extracted in this review, the relative contribution of these factors could not be quantified. It should, however, be noted that the reporting of outcomes within this domain was uneven. Efficacy based on TCM syndrome differentiation was assessed in 11 studies (3.4%), whereas overall TCM syndrome scores were reported in only three studies (0.9%). Tongue and pulse manifestations, despite their relevance to syndrome differentiation, were assessed in three (0.9%) and two studies (0.6%), respectively. Methodological recommendations for core outcome sets in integrative medicine have proposed the development of a core TCM syndrome set alongside general efficacy and safety outcomes, with explicit consideration of what should be measured, how it should be measured, and at which time points (Qiu et al., 2021). More recent core outcome set research has similarly incorporated standardized Chinese medicine syndrome assessment as a distinct component of treatment evaluation (Qiu et al., 2026). These developments, together with our findings, suggest that future chronic pharyngitis trials should focus not simply on increasing the number of TCM-specific outcomes, but on selecting a limited set of clearly defined and clinically interpretable syndrome measures.

Safety outcomes were insufficiently represented across the included trials. Notably, none of the 69 studies reporting adverse reactions clearly specified the time points at which these outcomes were assessed. The absence of a defined observation period limits interpretation of safety outcomes, as it remains unclear whether adverse events were monitored during treatment, assessed only at treatment completion, or followed after treatment cessation. This may reduce comparability across trials and hinder the identification of delayed or persistent adverse events. Future trials should therefore prespecify and clearly report appropriate assessment time points or observation windows for safety outcomes.

The cross-analysis further indicated that outcome assessment timing was closely aligned with treatment duration, with most trials evaluating outcomes at treatment completion and relatively few extending assessment into the post-treatment period. This pattern suggests that assessment schedules were largely treatment-driven and therefore primarily captured immediate treatment responses rather than the durability of treatment effects. For a chronic condition such as chronic pharyngitis, limited post-treatment follow-up may hinder the evaluation of sustained symptom improvement and recurrence. Future trials should therefore prespecify clinically meaningful assessment schedules that include both end-of-treatment and appropriate post-treatment follow-up time points, and the standardization of assessment timing should also be considered in the development of a core outcome set.

Long-term prognosis outcomes were also infrequently assessed, appearing in 33 studies (10.3%). Recurrence rate accounted for nearly all reporting within this domain (n = 32, 10.0%), whereas prognosis was assessed more broadly in only one study (0.3%). This pattern may partly reflect the predominantly short treatment and assessment periods of the included trials, as well as the practical tension between maintaining feasible data collection and extending participant follow-up. Nevertheless, an immediate treatment response does not necessarily indicate that the benefit will persist after treatment has ended. Future evaluations should therefore consider sustained response, time to recurrence, the frequency of repeated treatment, and subsequent healthcare use, rather than treating recurrence as a simple binary event.

Economic evaluation was particularly uncommon, being reported in only two studies (0.6%). Both studies calculated a cost-effectiveness ratio, while drug cost and the incremental cost-effectiveness ratio were each assessed in only one study. The limited reporting of these outcomes may be related to the additional expertise, prospective resource-use data, and longer observation periods required for trial-based economic evaluation. Beyond these methodological requirements, broader systemic factors may also contribute to the scarcity of economic evaluations. Many TCM trials are relatively small, short-term efficacy studies and may lack the funding, infrastructure, or prespecified design needed for comprehensive collection of resource-use and cost data. In addition, limited emphasis on economic outcomes in trial protocols, reporting standards, and publication practices may further reduce incentives to incorporate formal economic evaluation alongside clinical effectiveness assessments. A recent systematic review found that economic evaluations were rarely incorporated into randomized implementation trials and emphasized the need to plan costing methods and economic data collection alongside the clinical study from its design stage (Carrandi et al., 2024). This issue is also relevant to the evidence-based integration of traditional medicine into healthcare systems, for which effectiveness alone may be insufficient without information on affordability, resource requirements, and overall value. The WHO Global Traditional Medicine Strategy 2025–2034 similarly identifies the strengthening of evidence and optimization of the health-system and cross-sectoral value of traditional medicine as major strategic priorities (Wong et al., 2025). Although only two eligible trial registrations were identified in our review, future chronic pharyngitis trials should prospectively specify long-term and economic outcomes in their protocols and registry records. Collecting data on recurrence, retreatment, healthcare utilization, direct medical expenditure, patient costs, and incremental cost-effectiveness alongside clinical outcomes would provide a more complete basis for determining whether treatment benefits are both durable and economically worthwhile.

The substantial variation in outcome selection and reporting identified across trials evaluating traditional Chinese medicine (TCM) interventions for chronic pharyngitis represents a major obstacle to assessing treatment effects and integrating evidence. This inconsistency is evident in both the vast array of distinct outcomes and their infrequent reporting across studies. While it is neither feasible nor appropriate for every trial to assess the full range of available outcomes, the absence of an agreed minimum set limits meaningful comparisons, weakens meta-analyses, and wastes research resources. Therefore, the findings of this scoping review provide an empirical basis for developing a core outcome set (COS) for TCM interventions in chronic pharyngitis. Before the Delphi survey, the candidate outcome list will undergo a structured reduction process. Outcome terms identified in this review will first be further consolidated by removing duplicates and merging synonymous expressions that represent the same underlying clinical construct, while clinically distinct outcomes will be retained and organized within the predefined outcome domains. The resulting list will then be reviewed alongside findings from stakeholder interviews to identify additional patient- or clinician-important outcomes and to refine wording and conceptual overlap. A multidisciplinary steering group will subsequently review the candidate list for redundancy, clarity, and comprehensiveness before it is presented to stakeholders for Delphi prioritization. Importantly, outcomes will not be excluded solely because they were infrequently reported in previous trials, as low reporting frequency may itself reflect gaps in existing research rather than limited stakeholder importance.

Establishing this COS would define the essential outcomes required in all relevant trials—without restricting investigators from exploring additional ones—thereby facilitating consistent evidence synthesis. However, identifying what to measure must be paired with determining how to measure it efficiently. Continued methodological innovation, including concise validated instruments and digital assessment approaches, is necessary. Ultimately, combining standardized outcome selection with efficient measurement methods will preserve research comparability while minimizing the burden on participants and investigators.

The present review marks the initial phase in establishing the proposed COS. The identified outcomes serve as a solid groundwork for future consensus-building processes, such as Delphi surveys (Remus et al., 2021; Webbe et al., 2023) and formal consensus meetings structured according to COMET Initiative guidelines (Williamson et al., 2017). Engaging a broad spectrum of stakeholders—such as clinicians, researchers, patients, and policy-makers—helps ensure the final COS encompasses clinical priorities, patient-centered domains like quality of life, and health economic considerations (Dodd et al., 2023). Adopting a standardized, stakeholder-informed COS can align Traditional Chinese Medicine (TCM) research with global reporting criteria, improving transparency and cross-study comparability. Moreover, routinely applying predetermined core outcomes minimizes selective outcome reporting and enhances evidence completeness, ultimately increasing the reliability and practical utility of trial findings (Prinsen et al., 2016). Taken together, these efforts advance rigorous, patient-centered, and policy-relevant evidence synthesis in TCM.

Our findings also underscore the need for more complete and timely updating of trial registry records for studies of traditional Chinese medicine interventions for chronic pharyngitis. Ideally, registry entries should provide sufficiently detailed information on prespecified outcomes, corresponding measurement instruments and assessment time points, trial status, and resulting publications. Comprehensive registration would improve the transparency of outcome selection and facilitate comparisons between prespecified and published outcomes, thereby providing a more reliable basis for identifying outcomes that are commonly evaluated across chronic pharyngitis trials.

Although risk-of-bias assessment is not mandatory for scoping reviews, the RoB two findings provide useful context for COS development. The frequent concerns related to randomization indicate limitations in the methodological rigor of the existing evidence base, while concerns regarding subjective outcome measurement are more directly relevant to outcome standardization. These findings suggest that the frequency with which an outcome has been reported in previous trials should not be interpreted as evidence of its importance or suitability for inclusion in a COS. Rather, candidate outcomes identified in this review should be further prioritized through stakeholder consensus, with particular attention to clear outcome definitions, standardized assessment approaches, and appropriate measurement time points.

A primary strength of this review lies in its strict compliance with established scoping review standards (Tricco et al., 2018) alongside the COMET Initiative recommendations (Williamson et al., 2017). Furthermore, our search strategy systematically covered both electronic academic databases and clinical trial registries, enabling the retrieval of secondary or exploratory outcomes that are frequently omitted from published papers. Finally, by restricting the eligibility criteria strictly to randomized controlled trials (RCTs) on chronic pharyngitis, this study prioritizes evidence derived from high-quality experimental architectures, thereby bolstering internal validity and minimizing potential confounding influences typical of observational designs.

## 5 Limitations

To properly contextualize these observations and support subsequent core outcome set development, several limitations of this review warrant consideration. Primarily, because our search strategy was confined to published reports and specified registry databases, outcomes that were selectively omitted, poorly documented, or published in languages other than English or Chinese may have escaped detection. Additionally, standardizing ambiguous outcome descriptors into umbrella domains inherently required subjective interpretation by the research team, creating potential reviewer bias despite structured consensus procedures. Furthermore, since this study focused solely on identifying and mapping reported outcomes, we did not formally evaluate the measurement properties (e.g., reliability and validity) of the corresponding tools; hence, reporting frequency cannot serve as a reliable proxy for clinical significance.

Lastly, incomplete reporting of TCM preparation characteristics, including the composition of botanical drugs and other medicinal constituents, medicinal parts, dosage, processing or extraction procedures, and quality-control information, limited the comparability of similarly named interventions. Consequently, the extent to which differences in preparation composition and processing may have influenced outcome selection and reporting patterns could not be robustly assessed.

## 6 Conclusions

Substantial variation was identified in the selection and reporting of outcomes across RCTs evaluating TCM interventions for chronic pharyngitis. A total of 1,204 individual outcomes were identified and classified into seven domains, with most outcomes reported in only a small proportion of trials. This marked heterogeneity indicates limited consensus regarding which outcomes should be prioritized and restricts meaningful comparison and synthesis across studies. Important areas, including health-related quality of life, safety, patient-reported outcomes, and long-term outcomes, also remain insufficiently represented. Future trials should prespecify and clearly report assessment time points or observation windows for safety outcomes, and this should be considered an important target for improving outcome reporting standards. These findings provide an empirical foundation for the development of a core outcome set for chronic pharyngitis. A consensus-based COS incorporating outcomes of relevance to both clinical research and patients could promote greater consistency in outcome selection, measurement, and reporting and support the generation of more comparable, transparent, and clinically meaningful evidence(Duan and Franco, 2026).

## Data Availability

The original contributions presented in the study are included in the article/Supplementary Material, further inquiries can be directed to the corresponding authors.
