# Supplementary material for: Outcomes reported in randomized controlled trials of traditional Chinese medicine for chronic pharyngitis: a scoping review
Source: Front Pharmacol. 2026 Oct 1;17:1958608. doi: 10.3389/fphar.2026.1958608 (PMC13669160; doi:10.3389/fphar.2026.1958608)
Supplement: Supplementary file 1 [file DataSheet1.pdf]

ConPhyMP checklist of information for reporting plant material and its initial processing [1,2] (relevant for all studies on medicinal and food plants including extract types A, B, and C)

SECTION/TOPIC	ITEM NO.	CHECKLIST ITEM	YES	NO	NOT APPLICABLE	PAGE NO., IF ANY
Title and abstract	1	A clear and concise title including an informative abstract and balanced summary.	✓	○	○	
Description of the botanical drug and taxonomic authentication	2	Botanical or morphological authentication of the plant material (desirable is a combination with DNA barcoding, e.g., PCR, RFLP, genome sequencing) and the information must be included in a separate section of Material and Methods, if applicable, combined with the information required under item 3:	○	○	✓	
Description of the extract and extraction process	3	A separate section in Material and Methods, covers the relevant information on the material investigated, including the full species name(s), authorites and family; e.g. *Salvia miltorrhiza* Bunge [Lamiaceae; *Salviae miltorrhizae radix et rhizoma*], and on the processing and extraction of the crude drug including the traditional processing of the material used medicinally (fumigation, steaming, roasting, cooking, frying, etc.).	○	○	✓	
Documentation of the legal basis for collection and processing	4	Full compliance with the Nagoya protocol, CITES, and all associated treaties including phytosanitary regulations.	○	○	✓	
Description of product characteristics, in case of a finished (commercial) product	5	Information on the characteristics of the commercial products including batch number and date of production/best by information and regulatory status.	✓	○	○	

Note: Please also include here the following information about your submitted manuscript:

Name of the journal:	Frontiers in Pharmacology
Date of the enquiry:	2026.08.01
Title of the manuscript:	Outcomes Reported in Randomized Controlled Trials of Trac
List of the authors:	Danni Li

[1] Please acknowledge/cite this as follows: Heinrich M, Jalil B, Abdel-Tawab M, Echeverria J, Kulić Ž, McGaw LJ, et al. Best Practice in the chemical characterisation of extracts used in pharmacological and toxicological research—The ConPhyMP—Guidelines. Frontiers in Pharmacology. 2022;13:953205. https://doi.org/10.3389/fphar.2022.953205

[2] We strongly recommend reading this checklist in conjuncton with ConPhyMP 2022 explanaton and elaboraton for important clarifications on all items. If relevant, we also recommend after reading Heinrich et al. (2020) Best practice in research—Overcoming common challenges in phytopharmacological research. Journal of Ethnopharmacology. 2020;246:112230. https://doi.org/10.1016/j.jep.2019.112230

ConPhyMP checklist of information for reporting plant material and its initial processing [1,2] (relevant for all studies on medicinal and food plants including extract types A, B, and C)

SECTION/TOPIC	ITEM NO.	CHECKLIST ITEM	YES	NO	NOT APPLICABLE	PAGE NO., IF ANY
Title and abstract	1	A clear and concise title including an informative abstract and balanced summary.	✔			
Description of the botanical drug and taxonomic authentication	2	Botanical or morphological authentication of the plant material (desirable is a combination with DNA barcoding, e.g., PCR, RFLP, genome sequencing) and the information must be included in a separate section of Material and Methods, if applicable, combined with the information required under item 3:			✔	
Description of the extract and extraction process	3	A separate section in Material and Methods, covers the relevant information on the material investigated, including the full species name(s), authorites and family; e.g. *Salvia miltorrhiza* Bunge [Lamiaceae; *Salviae miltorrhizae radix et rhizoma*], and on the processing and extraction of the crude drug including the traditional processing of the material used medicinally (fumigation, steaming, roasting, cooking, frying, etc.).			✔	
Documentation of the legal basis for collection and processing	4	Full compliance with the Nagoya protocol, CITES, and all associated treaties including phytosanitary regulations.			✔	
Description of product characteristics, in case of a finished (commercial) product	5	Information on the characteristics of the commercial products including batch number and date of production/best by information and regulatory status.	✔			

Note: Please also include here the following information about your submitted manuscript:

Name of the journal:	Frontiers in Pharmacology
Date of the enquiry:	2026.08.01
Title of the manuscript:	Outcomes Reported in Randomized Controlled Trials of Trac
List of the authors:	Danni Li

[1] Please acknowledge/cite this as follows: Heinrich M, Jalil B, Abdel-Tawab M, Echeverria J, Kulić Ž, McGaw LJ, et al. Best Practice in the chemical characterisation of extracts used in pharmacological and toxicological research—The ConPhyMP—Guidelines. Frontiers in Pharmacology. 2022;13:953205. https://doi.org/10.3389/fphar.2022.953205

[2] We strongly recommend reading this checklist in conjuncton with ConPhyMP 2022 explanaton and elaboraton for important clarifications on all items. If relevant, we also recommend after reading Heinrich et al. (2020) Best practice in research—Overcoming common challenges in phytopharmacological research. Journal of Ethnopharmacology. 2020;246:112230. https://doi.org/10.1016/j.jep.2019.112230

ConPhyMP checklist of information for reporting plant material and its initial processing [1,2] (relevant for all studies on medicinal and food plants including extract types A, B, and C)

SECTION/TOPIC	ITEM NO.	CHECKLIST ITEM	YES	NO	NOT APPLICABLE	PAGE NO., IF ANY
Title and abstract	1	A clear and concise title including an informative abstract and balanced summary.	✔			
Description of the botanical drug and taxonomic authentication	2	Botanical or morphological authentication of the plant material (desirable is a combination with DNA barcoding, e.g., PCR, RFLP, genome sequencing) and the information must be included in a separate section of Material and Methods, if applicable, combined with the information required under item 3:			✔	
Description of the extract and extraction process	3	A separate section in Material and Methods, covers the relevant information on the material investigated, including the full species name(s), authorites and family; e.g. *Salvia miltorrhiza* Bunge [Lamiaceae; *Salviae miltorrhizae radix et rhizoma*], and on the processing and extraction of the crude drug including the traditional processing of the material used medicinally (fumigation, steaming, roasting, cooking, frying, etc.).			✔	
Documentation of the legal basis for collection and processing	4	Full compliance with the Nagoya protocol, CITES, and all associated treaties including phytosanitary regulations.			✔	
Description of product characteristics, in case of a finished (commercial) product	5	Information on the characteristics of the commercial products including batch number and date of production/best by information and regulatory status.	✔			

Note: Please also include here the following information about your submitted manuscript:

Name of the journal:	Frontiers in Pharmacology
Date of the enquiry:	2026.08.01
Title of the manuscript:	Outcomes Reported in Randomized Controlled Trials of Tra(
List of the authors:	Danni Li

[1] Please acknowledge/cite this as follows: Heinrich M, Jalil B, Abdel-Tawab M, Echeverria J, Kulić Ž, McGaw LJ, et al. Best Practice in the chemical characterisation of extracts used in pharmacological and toxicological research—The ConPhyMP—Guidelines. Frontiers in Pharmacology. 2022;13:953205. https://doi.org/10.3389/fphar.2022.953205

[2] We strongly recommend reading this checklist in conjuncton with ConPhyMP 2022 explanaton and elaboraton for important clarifications on all items. If relevant, we also recommend after reading Heinrich et al. (2020) Best practice in research—Overcoming common challenges in phytopharmacological research. Journal of Ethnopharmacology. 2020;246:112230. https://doi.org/10.1016/j.jep.2019.112230

ConPhyMP checklist of information for reporting plant material and its initial processing [1,2] (relevant for all studies on medicinal and food plants including extract types A, B, and C)

SECTION/TOPIC	ITEM NO.	CHECKLIST ITEM	YES	NO	NOT APPLICABLE	PAGE NO., IF ANY
Title and abstract	1	A clear and concise title including an informative abstract and balanced summary.	✔			
Description of the botanical drug and taxonomic authentication	2	Botanical or morphological authentication of the plant material (desirable is a combination with DNA barcoding, e.g., PCR, RFLP, genome sequencing) and the information must be included in a separate section of Material and Methods, if applicable, combined with the information required under item 3:			✔	
Description of the extract and extraction process	3	A separate section in Material and Methods, covers the relevant information on the material investigated, including the full species name(s), authorites and family; e.g. *Salvia miltorrhiza* Bunge [Lamiaceae; *Salviae miltorrhizae radix et rhizoma*], and on the processing and extraction of the crude drug including the traditional processing of the material used medicinally (fumigation, steaming, roasting, cooking, frying, etc.).			✔	
Documentation of the legal basis for collection and processing	4	Full compliance with the Nagoya protocol, CITES, and all associated treaties including phytosanitary regulations.			✔	
Description of product characteristics, in case of a finished (commercial) product	5	Information on the characteristics of the commercial products including batch number and date of production/best by information and regulatory status.	✔			

Note: Please also include here the following information about your submitted manuscript:

Name of the journal:	Frontiers in Pharmacology
Date of the enquiry:	2026.08.01
Title of the manuscript:	Outcomes Reported in Randomized Controlled Trials of Trad
List of the authors:	Danni Li

[1] Please acknowledge/cite this as follows: Heinrich M, Jalil B, Abdel-Tawab M, Echeverria J, Kulić Ž, McGaw LJ, et al. Best Practice in the chemical characterisation of extracts used in pharmacological and toxicological research—The ConPhyMP—Guidelines. Frontiers in Pharmacology. 2022;13:953205. https://doi.org/10.3389/fphar.2022.953205

[2] We strongly recommend reading this checklist in conjuncton with ConPhyMP 2022 explanaton and elaboraton for important clarifications on all items. If relevant, we also recommend after reading Heinrich et al. (2020) Best practice in research—Overcoming common challenges in phytopharmacological research. Journal of Ethnopharmacology. 2020;246:112230. https://doi.org/10.1016/j.jep.2019.112230

ConPhyMP checklist of information for reporting plant material and its initial processing [1,2] (relevant for all studies on medicinal and food plants including extract types A, B, and C)

SECTION/TOPIC	ITEM NO.	CHECKLIST ITEM	YES	NO	NOT APPLICABLE	PAGE NO., IF ANY
Title and abstract	1	A clear and concise title including an informative abstract and balanced summary.	✔			
Description of the botanical drug and taxonomic authentication	2	Botanical or morphological authentication of the plant material (desirable is a combination with DNA barcoding, e.g., PCR, RFLP, genome sequencing) and the information must be included in a separate section of Material and Methods, if applicable, combined with the information required under item 3:			✔	
Description of the extract and extraction process	3	A separate section in Material and Methods, covers the relevant information on the material investigated, including the full species name(s), authorites and family; e.g. *Salvia miltorrhiza* Bunge [Lamiaceae; *Salviae miltorrhizae radix et rhizoma*], and on the processing and extraction of the crude drug including the traditional processing of the material used medicinally (fumigation, steaming, roasting, cooking, frying, etc.).			✔	
Documentation of the legal basis for collection and processing	4	Full compliance with the Nagoya protocol, CITES, and all associated treaties including phytosanitary regulations.			✔	
Description of product characteristics, in case of a finished (commercial) product	5	Information on the characteristics of the commercial products including batch number and date of production/best by information and regulatory status.	✔			

Note: Please also include here the following information about your submitted manuscript:

Name of the journal:	Frontiers in Pharmacology
Date of the enquiry:	2026.08.01
Title of the manuscript:	Outcomes Reported in Randomized Controlled Trials of Trad
List of the authors:	Danni Li

[1] Please acknowledge/cite this as follows: Heinrich M, Jalil B, Abdel-Tawab M, Echeverria J, Kulić Ž, McGaw LJ, et al. Best Practice in the chemical characterisation of extracts used in pharmacological and toxicological research—The ConPhyMP—Guidelines. Frontiers in Pharmacology. 2022;13:953205. https://doi.org/10.3389/fphar.2022.953205

[2] We strongly recommend reading this checklist in conjuncton with ConPhyMP 2022 explanaton and elaboraton for important clarifications on all items. If relevant, we also recommend after reading Heinrich et al. (2020) Best practice in research—Overcoming common challenges in phytopharmacological research. Journal of Ethnopharmacology. 2020;246:112230. https://doi.org/10.1016/j.jep.2019.112230

ConPhyMP checklist of information for reporting plant material and its initial processing [1,2] (relevant for all studies on medicinal and food plants including extract types A, B, and C)

SECTION/TOPIC	ITEM NO.	CHECKLIST ITEM	YES	NO	NOT APPLICABLE	PAGE NO., IF ANY
Title and abstract	1	A clear and concise title including an informative abstract and balanced summary.	✔			
Description of the botanical drug and taxonomic authentication	2	Botanical or morphological authentication of the plant material (desirable is a combination with DNA barcoding, e.g., PCR, RFLP, genome sequencing) and the information must be included in a separate section of Material and Methods, if applicable, combined with the information required under item 3:			✔	
Description of the extract and extraction process	3	A separate section in Material and Methods, covers the relevant information on the material investigated, including the full species name(s), authorites and family; e.g. *Salvia miltorrhiza* Bunge [Lamiaceae; *Salviae miltorrhizae radix et rhizoma*], and on the processing and extraction of the crude drug including the traditional processing of the material used medicinally (fumigation, steaming, roasting, cooking, frying, etc.).			✔	
Documentation of the legal basis for collection and processing	4	Full compliance with the Nagoya protocol, CITES, and all associated treaties including phytosanitary regulations.			✔	
Description of product characteristics, in case of a finished (commercial) product	5	Information on the characteristics of the commercial products including batch number and date of production/best by information and regulatory status.	✔			

Note: Please also include here the following information about your submitted manuscript:

Name of the journal:	Frontiers in Pharmacology
Date of the enquiry:	2026.08.01
Title of the manuscript:	Outcomes Reported in Randomized Controlled Trials of Trac
List of the authors:	Danni Li

[1] Please acknowledge/cite this as follows: Heinrich M, Jalil B, Abdel-Tawab M, Echeverria J, Kulić Ž, McGaw LJ, et al. Best Practice in the chemical characterisation of extracts used in pharmacological and toxicological research—The ConPhyMP—Guidelines. Frontiers in Pharmacology. 2022;13:953205. https://doi.org/10.3389/fphar.2022.953205

[2] We strongly recommend reading this checklist in conjuncton with ConPhyMP 2022 explanaton and elaboraton for important clarifications on all items. If relevant, we also recommend after reading Heinrich et al. (2020) Best practice in research—Overcoming common challenges in phytopharmacological research. Journal of Ethnopharmacology. 2020;246:112230. https://doi.org/10.1016/j.jep.2019.112230

ConPhyMP checklist of information for reporting plant material and its initial processing [1,2] (relevant for all studies on medicinal and food plants including extract types A, B, and C)

SECTION/TOPIC	ITEM NO.	CHECKLIST ITEM	YES	NO	NOT APPLICABLE	PAGE NO., IF ANY
Title and abstract	1	A clear and concise title including an informative abstract and balanced summary.	✔			
Description of the botanical drug and taxonomic authentication	2	Botanical or morphological authentication of the plant material (desirable is a combination with DNA barcoding, e.g., PCR, RFLP, genome sequencing) and the information must be included in a separate section of Material and Methods, if applicable, combined with the information required under item 3:			✔	
Description of the extract and extraction process	3	A separate section in Material and Methods, covers the relevant information on the material investigated, including the full species name(s), authorites and family; e.g. *Salvia miltorrhiza* Bunge [Lamiaceae; *Salviae miltorrhizae radix et rhizoma*], and on the processing and extraction of the crude drug including the traditional processing of the material used medicinally (fumigation, steaming, roasting, cooking, frying, etc.).			✔	
Documentation of the legal basis for collection and processing	4	Full compliance with the Nagoya protocol, CITES, and all associated treaties including phytosanitary regulations.			✔	
Description of product characteristics, in case of a finished (commercial) product	5	Information on the characteristics of the commercial products including batch number and date of production/best by information and regulatory status.	✔			

Note: Please also include here the following information about your submitted manuscript:

Name of the journal:	Frontiers in Pharmacology
Date of the enquiry:	2026.08.01
Title of the manuscript:	Outcomes Reported in Randomized Controlled Trials of Trac
List of the authors:	Danni Li

[1] Please acknowledge/cite this as follows: Heinrich M, Jalil B, Abdel-Tawab M, Echeverria J, Kulić Ž, McGaw LJ, et al. Best Practice in the chemical characterisation of extracts used in pharmacological and toxicological research—The ConPhyMP—Guidelines. Frontiers in Pharmacology. 2022;13:953205. https://doi.org/10.3389/fphar.2022.953205

[2] We strongly recommend reading this checklist in conjuncton with ConPhyMP 2022 explanaton and elaboraton for important clarifications on all items. If relevant, we also recommend after reading Heinrich et al. (2020) Best practice in research—Overcoming common challenges in phytopharmacological research. Journal of Ethnopharmacology. 2020;246:112230. https://doi.org/10.1016/j.jep.2019.112230

ConPhyMP checklist of information for reporting plant material and its initial processing [1,2] (relevant for all studies on medicinal and food plants including extract types A, B, and C)

SECTION/TOPIC	ITEM NO.	CHECKLIST ITEM	YES	NO	NOT APPLICABLE	PAGE NO., IF ANY
Title and abstract	1	A clear and concise title including an informative abstract and balanced summary.	✔	☐	☐	
Description of the botanical drug and taxonomic authentication	2	Botanical or morphological authentication of the plant material (desirable is a combination with DNA barcoding, e.g., PCR, RFLP, genome sequencing) and the information must be included in a separate section of Material and Methods, if applicable, combined with the information required under item 3:	☐	☐	✔	
Description of the extract and extraction process	3	A separate section in Material and Methods, covers the relevant information on the material investigated, including the full species name(s), authorites and family; e.g. *Salvia miltorrhiza* Bunge [Lamiaceae; *Salviae miltorrhizae radix et rhizoma*], and on the processing and extraction of the crude drug including the traditional processing of the material used medicinally (fumigation, steaming, roasting, cooking, frying, etc.).	☐	☐	✔	
Documentation of the legal basis for collection and processing	4	Full compliance with the Nagoya protocol, CITES, and all associated treaties including phytosanitary regulations.	☐	☐	✔	
Description of product characteristics, in case of a finished (commercial) product	5	Information on the characteristics of the commercial products including batch number and date of production/best by information and regulatory status.	✔	☐	☐	

Note: Please also include here the following information about your submitted manuscript:

Name of the journal:	Frontiers in Pharmacology
Date of the enquiry:	2026.08.01
Title of the manuscript:	Outcomes Reported in Randomized Controlled Trials of Trac
List of the authors:	Danni Li

[1] Please acknowledge/cite this as follows: Heinrich M, Jalil B, Abdel-Tawab M, Echeverria J, Kulić Ž, McGaw LJ, et al. Best Practice in the chemical characterisation of extracts used in pharmacological and toxicological research—The ConPhyMP—Guidelines. Frontiers in Pharmacology. 2022;13:953205. https://doi.org/10.3389/fphar.2022.953205

[2] We strongly recommend reading this checklist in conjuncton with ConPhyMP 2022 explanaton and elaboraton for important clarifications on all items. If relevant, we also recommend after reading Heinrich et al. (2020) Best practice in research—Overcoming common challenges in phytopharmacological research. Journal of Ethnopharmacology. 2020;246:112230. https://doi.org/10.1016/j.jep.2019.112230

ConPhyMP checklist of information for reporting plant material and its initial processing [1,2] (relevant for all studies on medicinal and food plants including extract types A, B, and C)

SECTION/TOPIC	ITEM NO.	CHECKLIST ITEM	YES	NO	NOT APPLICABLE	PAGE NO., IF ANY
Title and abstract	1	A clear and concise title including an informative abstract and balanced summary.	✔	☐	☐	
Description of the botanical drug and taxonomic authentication	2	Botanical or morphological authentication of the plant material (desirable is a combination with DNA barcoding, e.g., PCR, RFLP, genome sequencing) and the information must be included in a separate section of Material and Methods, if applicable, combined with the information required under item 3:	☐	☐	✔	
Description of the extract and extraction process	3	A separate section in Material and Methods, covers the relevant information on the material investigated, including the full species name(s), authorites and family; e.g. *Salvia miltorrhiza* Bunge [Lamiaceae; *Salviae miltorrhizae radix et rhizoma*], and on the processing and extraction of the crude drug including the traditional processing of the material used medicinally (fumigation, steaming, roasting, cooking, frying, etc.).	☐	☐	✔	
Documentation of the legal basis for collection and processing	4	Full compliance with the Nagoya protocol, CITES, and all associated treaties including phytosanitary regulations.	☐	☐	✔	
Description of product characteristics, in case of a finished (commercial) product	5	Information on the characteristics of the commercial products including batch number and date of production/best by information and regulatory status.	✔	☐	☐	

Note: Please also include here the following information about your submitted manuscript:

Name of the journal:	Frontiers in Pharmacology
Date of the enquiry:	2026.08.01
Title of the manuscript:	Outcomes Reported in Randomized Controlled Trials of Trad
List of the authors:	Danni Li

[1] Please acknowledge/cite this as follows: Heinrich M, Jalil B, Abdel-Tawab M, Echeverria J, Kulić Ž, McGaw LJ, et al. Best Practice in the chemical characterisation of extracts used in pharmacological and toxicological research—The ConPhyMP—Guidelines. Frontiers in Pharmacology. 2022;13:953205. https://doi.org/10.3389/fphar.2022.953205

[2] We strongly recommend reading this checklist in conjuncton with ConPhyMP 2022 explanaton and elaboraton for important clarifications on all items. If relevant, we also recommend after reading Heinrich et al. (2020) Best practice in research—Overcoming common challenges in phytopharmacological research. Journal of Ethnopharmacology. 2020;246:112230. https://doi.org/10.1016/j.jep.2019.112230

ConPhyMP checklist of information for reporting plant material and its initial processing [1,2] (relevant for all studies on medicinal and food plants including extract types A, B, and C)

SECTION/TOPIC	ITEM NO.	CHECKLIST ITEM	YES	NO	NOT APPLICABLE	PAGE NO., IF ANY
Title and abstract	1	A clear and concise title including an informative abstract and balanced summary.	✔	☐	☐	
Description of the botanical drug and taxonomic authentication	2	Botanical or morphological authentication of the plant material (desirable is a combination with DNA barcoding, e.g., PCR, RFLP, genome sequencing) and the information must be included in a separate section of Material and Methods, if applicable, combined with the information required under item 3:	☐	☐	✔	
Description of the extract and extraction process	3	A separate section in Material and Methods, covers the relevant information on the material investigated, including the full species name(s), authorites and family; e.g. *Salvia miltorrhiza* Bunge [Lamiaceae; *Salviae miltorrhizae radix et rhizoma*], and on the processing and extraction of the crude drug including the traditional processing of the material used medicinally (fumigation, steaming, roasting, cooking, frying, etc.).	☐	☐	✔	
Documentation of the legal basis for collection and processing	4	Full compliance with the Nagoya protocol, CITES, and all associated treaties including phytosanitary regulations.	☐	☐	✔	
Description of product characteristics, in case of a finished (commercial) product	5	Information on the characteristics of the commercial products including batch number and date of production/best by information and regulatory status.	✔	☐	☐	

Note: Please also include here the following information about your submitted manuscript:

Name of the journal:	Frontiers in Pharmacology
Date of the enquiry:	2026.08.01
Title of the manuscript:	Outcomes Reported in Randomized Controlled Trials of Trad
List of the authors:	Danni Li

[1] Please acknowledge/cite this as follows: Heinrich M, Jalil B, Abdel-Tawab M, Echeverria J, Kulić Ž, McGaw LJ, et al. Best Practice in the chemical characterisation of extracts used in pharmacological and toxicological research—The ConPhyMP—Guidelines. Frontiers in Pharmacology. 2022;13:953205. https://doi.org/10.3389/fphar.2022.953205

[2] We strongly recommend reading this checklist in conjuncton with ConPhyMP 2022 explanaton and elaboraton for important clarifications on all items. If relevant, we also recommend after reading Heinrich et al. (2020) Best practice in research—Overcoming common challenges in phytopharmacological research. Journal of Ethnopharmacology. 2020;246:112230. https://doi.org/10.1016/j.jep.2019.112230

ConPhyMP checklist of items for conducting and reporting analytical methods[1,2] relevant for extract type A (for species or botanical drugs covered in a monograph in one of the national or regional pharmacopoeias)

SECTION/TOPIC	ITEM NO.	CHECKLIST ITEM	YES	NO	NOT APPLICABLE	PAGE NO., IF ANY
Type of extract	1	A – Confirm that the species or botanical drug under investigation is covered in a monograph in one of the national or regional pharmacopoeias.	✓			
Preferred/main methods for extract characterisation/ chemical analysis	2	Compliance with pharmacopoeial standards to be followed:				
		(a) The description of the active ingredients in the botanical drug (if known) or analytical marker compounds as defined.			✓	
		(b) An analysis as defined in the monograph is needed if the extract has not been supplied with a certificate.			✓	
		(c) If the preparation was purchased, the manufacturer and certificate of analysis need to be included.			✓	
		Including either the preferred or alternative approaches for characterisation:				
		(a) Triple chemical fingerprinting methods, each with one or more detection parameters.			✓	
		(b) Quantification of at least two marker compounds (unless this is not feasible, evidence needs to be provided), and justification of the choice of markers (if applicable).			✓	
Alternative methods for extract characterisation/chemical analysis	3	(a) Single chemical fingerprinting method with at least three different detection parameters (i.e., altered detection parameters, like TLC/HPTLC with different staining reagents and/or UV excitation wave-lengths, HPLC-DAD/LCDAD with different wavelengths). The same applies to coupling MS or NMR to chromatographic techniques.			✓	
		(b) Quantification of at least two marker compounds (unless this is not feasible, evidence needs to be provided), and justification of the choice of markers (if applicable).			✓	
Use of reference standards	4	(a) Direct overlay of the chromatogram of the sample with that of an officially specified reference standard (if applicable).<br>(b) Chromatographic fingerprinting: Direct overlay of the chromato-gram of the sample with that of official reference standards of the powdered plant material or the dry extract from the plant material.			✓	
Comparison of different extracts/ samples of the same plants	5	(a) Direct comparison of the chromatographic/spectroscopic system and/or scoring system for "similarity" to be followed.			✓	

Note: Please also include here the following information about your submitted manuscript:

Name of the journal:	Frontiers in Pharmacology
Date of the enquiry:	2026.08.01
Title of the manuscript:	Outcomes Reported in Randomized Controlled Trials of Tra
List of the authors:	Danni Li

[1] Please acknowledge/cite this as follows: Heinrich M, Jalil B, Abdel-Tawab M, Echeverria J, Kulić Ž, McGaw LJ, et al. Best Practice in the chemical characterisation of extracts used in pharmacological and toxicological research—The ConPhyMP—Guidelines. Frontiers in Pharmacology. 2022;13:953205. https://doi.org/10.3389/fphar.2022.953205

[2] We strongly recommend reading this checklist in conjuncton with ConPhyMP 2022 explanaton and elaboraton for important clarifications on all items. If relevant, we also recommend after reading Heinrich et al. (2020) Best practice in research—Overcoming common challenges in phytopharmacological research. Journal of Ethnopharmacology. 2020;246:112230. https://doi.org/10.1016/j.jep.2019.112230

ConPhyMP checklist of items for conducting and reporting analytical methods[1,2] relevant for extract type A (for species or botanical drugs covered in a monograph in one of the national or regional pharmacopoeias)

SECTION/TOPIC	ITEM NO.	CHECKLIST ITEM	YES	NO	NOT APPLICABLE	PAGE NO., IF ANY
Type of extract	1	A – Confirm that the species or botanical drug under investigation is covered in a monograph in one of the national or regional pharmacopoeias.	✔			
Preferred/main methods for extract characterisation/ chemical analysis	2	Compliance with pharmacopoeial standards to be followed:				
		(a) The description of the active ingredients in the botanical drug (if known) or analytical marker compounds as defined.			✔	
		(b) An analysis as defined in the monograph is needed if the extract has not been supplied with a certificate.			✔	
		(c) If the preparation was purchased, the manufacturer and certificate of analysis need to be included.			✔	
		Including either the preferred or alternative approaches for characterisation:				
		(a) Triple chemical fingerprinting methods, each with one or more detection parameters.			✔	
		(b) Quantification of at least two marker compounds (unless this is not feasible, evidence needs to be provided), and justification of the choice of markers (if applicable).			✔	
Alternative methods for extract characterisation/chemical analysis	3	(a) Single chemical fingerprinting method with at least three different detection parameters (i.e., altered detection parameters, like TLC/ HPTLC with different staining reagents and/or UV excitation wave-lengths, HPLC-DAD/LCDAD with different wavelengths). The same applies to coupling MS or NMR to chromatographic techniques.			✔	
		(b) Quantification of at least two marker compounds (unless this is not feasible, evidence needs to be provided), and justification of the choice of markers (if applicable).			✔	
Use of reference standards	4	(a) Direct overlay of the chromatogram of the sample with that of an officially specified reference standard (if applicable). (b) Chromatographic fingerprinting: Direct overlay of the chromato-gram of the sample with that of official reference standards of the powdered plant material or the dry extract from the plant material.			✔	
Comparison of different extracts/ samples of the same plants	5	(a) Direct comparison of the chromatographic/spectroscopic system and/or scoring system for "similarity" to be followed.			✔	

Note: Please also include here the following information about your submitted manuscript:

Name of the journal:	Frontiers in Pharmacology
Date of the enquiry:	2026.08.01
Title of the manuscript:	Outcomes Reported in Randomized Controlled Trials of Tra
List of the authors:	Danni Li

[1] Please acknowledge/cite this as follows: Heinrich M, Jalil B, Abdel-Tawab M, Echeverria J, Kulić Ž, McGaw LJ, et al. Best Practice in the chemical characterisation of extracts used in pharmacological and toxicological research—The ConPhyMP—Guidelines. Frontiers in Pharmacology. 2022;13:953205. https://doi.org/10.3389/fphar.2022.953205

[2] We strongly recommend reading this checklist in conjuncton with ConPhyMP 2022 explanaton and elaboraton for important clarifications on all items. If relevant, we also recommend after reading Heinrich et al. (2020) Best practice in research—Overcoming common challenges in phytopharmacological research. Journal of Ethnopharmacology. 2020;246:112230. https://doi.org/10.1016/j.jep.2019.112230

ConPhyMP checklist of items for conducting and reporting analytical methods[1,2] relevant for extract type A (for species or botanical drugs covered in a monograph in one of the national or regional pharmacopoeias)

SECTION/TOPIC	ITEM NO.	CHECKLIST ITEM	YES	NO	NOT APPLICABLE	PAGE NO., IF ANY
Type of extract	1	A – Confirm that the species or botanical drug under investigation is covered in a monograph in one of the national or regional pharmacopoeias.	✔			
Preferred/main methods for extract characterisation/ chemical analysis	2	Compliance with pharmacopoeial standards to be followed:				
		(a) The description of the active ingredients in the botanical drug (if known) or analytical marker compounds as defined.			✔	
		(b) An analysis as defined in the monograph is needed if the extract has not been supplied with a certificate.			✔	
		(c) If the preparation was purchased, the manufacturer and certificate of analysis need to be included.			✔	
		Including either the preferred or alternative approaches for characterisation:				
		(a) Triple chemical fingerprinting methods, each with one or more detection parameters.			✔	
		(b) Quantification of at least two marker compounds (unless this is not feasible, evidence needs to be provided), and justification of the choice of markers (if applicable).			✔	
Alternative methods for extract characterisation/chemical analysis	3	(a) Single chemical fingerprinting method with at least three different detection parameters (i.e., altered detection parameters, like TLC/HPTLC with different staining reagents and/or UV excitation wavelengths, HPLC-DAD/LCDAD with different wavelengths). The same applies to coupling MS or NMR to chromatographic techniques.			✔	
		(b) Quantification of at least two marker compounds (unless this is not feasible, evidence needs to be provided), and justification of the choice of markers (if applicable).			✔	
Use of reference standards	4	(a) Direct overlay of the chromatogram of the sample with that of an officially specified reference standard (if applicable). (b) Chromatographic fingerprinting: Direct overlay of the chromatogram of the sample with that of official reference standards of the powdered plant material or the dry extract from the plant material.			✔	
Comparison of different extracts/ samples of the same plants	5	(a) Direct comparison of the chromatographic/spectroscopic system and/or scoring system for "similarity" to be followed.			✔	

Note: Please also include here the following information about your submitted manuscript:

Name of the journal: Frontiers in Pharmacology

Date of the enquiry: 2026.08.01

Title of the manuscript: Outcomes Reported in Randomized Controlled Trials of Trad

List of the authors: Danni Li

[1] Please acknowledge/cite this as follows: Heinrich M, Jalil B, Abdel-Tawab M, Echeverria J, Kulić Ž, McGaw LJ, et al. Best Practice in the chemical characterisation of extracts used in pharmacological and toxicological research—The ConPhyMP—Guidelines. Frontiers in Pharmacology. 2022;13:953205. https://doi.org/10.3389/fphar.2022.953205

[2] We strongly recommend reading this checklist in conjuncton with ConPhyMP 2022 explanaton and elaboraton for important clarifications on all items. If relevant, we also recommend after reading Heinrich et al. (2020) Best practice in research—Overcoming common challenges in phytopharmacological research. Journal of Ethnopharmacology. 2020;246:112230. https://doi.org/10.1016/j.jep.2019.112230

ConPhyMP checklist of items for conducting and reporting analytical methods[1,2] relevant for extract type A (for species or botanical drugs covered in a monograph in one of the national or regional pharmacopoeias)

SECTION/TOPIC	ITEM NO.	CHECKLIST ITEM	YES	NO	NOT APPLICABLE	PAGE NO., IF ANY
Type of extract	1	A – Confirm that the species or botanical drug under investigation is covered in a monograph in one of the national or regional pharmacopoeias.	✔			
Preferred/main methods for extract characterisation/ chemical analysis	2	Compliance with pharmacopoeial standards to be followed:				
		(a) The description of the active ingredients in the botanical drug (if known) or analytical marker compounds as defined.			✔	
		(b) An analysis as defined in the monograph is needed if the extract has not been supplied with a certificate.			✔	
		(c) If the preparation was purchased, the manufacturer and certificate of analysis need to be included.			✔	
		Including either the preferred or alternative approaches for characterisation:				
		(a) Triple chemical fingerprinting methods, each with one or more detection parameters.			✔	
		(b) Quantification of at least two marker compounds (unless this is not feasible, evidence needs to be provided), and justification of the choice of markers (if applicable).			✔	
Alternative methods for extract characterisation/chemical analysis	3	(a) Single chemical fingerprinting method with at least three different detection parameters (i.e., altered detection parameters, like TLC/HPTLC with different staining reagents and/or UV excitation wave-lengths, HPLC-DAD/LCDAD with different wavelengths). The same applies to coupling MS or NMR to chromatographic techniques.			✔	
		(b) Quantification of at least two marker compounds (unless this is not feasible, evidence needs to be provided), and justification of the choice of markers (if applicable).			✔	
Use of reference standards	4	(a) Direct overlay of the chromatogram of the sample with that of an officially specified reference standard (if applicable). (b) Chromatographic fingerprinting: Direct overlay of the chromato-gram of the sample with that of official reference standards of the powdered plant material or the dry extract from the plant material.			✔	
Comparison of different extracts/ samples of the same plants	5	(a) Direct comparison of the chromatographic/spectroscopic system and/or scoring system for "similarity" to be followed.			✔	

Note: Please also include here the following information about your submitted manuscript:

Name of the journal:	Frontiers in Pharmacology
Date of the enquiry:	2026.08.01
Title of the manuscript:	Outcomes Reported in Randomized Controlled Trials of Tra
List of the authors:	Danni Li

[1] Please acknowledge/cite this as follows: Heinrich M, Jalil B, Abdel-Tawab M, Echeverria J, Kulić Ž, McGaw LJ, et al. Best Practice in the chemical characterisation of extracts used in pharmacological and toxicological research—The ConPhyMP—Guidelines. Frontiers in Pharmacology. 2022;13:953205. https://doi.org/10.3389/fphar.2022.953205

[2] We strongly recommend reading this checklist in conjuncton with ConPhyMP 2022 explanaton and elaboraton for important clarifications on all items. If relevant, we also recommend after reading Heinrich et al. (2020) Best practice in research—Overcoming common challenges in phytopharmacological research. Journal of Ethnopharmacology. 2020;246:112230. https://doi.org/10.1016/j.jep.2019.112230

ConPhyMP checklist of items for conducting and reporting analytical methods[1,2] relevant for extract type B (for species or botanical drugs widely used or traded without a monograph in a national or international pharmacopoeia)

SECTION/TOPIC	ITEM NO.	CHECKLIST ITEM	YES	NO	NOT APPLICABLE	PAGE NO., IF ANY
Type of extract	1	B – Confirm that the species or botanical drug under investigation is a widely used or traded one without a monograph in a national or international pharmacopoeia.	✓			
Preferred/ main methods for extract characterisation/ chemical analysis	2	(a) Triple chemical fingerprinting methods, each with one or more detection parameters.			✓	
		(b) Quantification of at least two marker compounds (unless this is not feasible, evidence needs to be provided), and justification of the choice of markers (if applicable).			✓	
Alternative methods for extract characterisation/chemical analysis	3	(a) Single chemical fingerprinting method, three different detection parameters should be provided (i.e., altered detection parameters, like TLC/HPTLC with different staining reagents and/or UV excitation wavelengths, HPLC-DAD/LC-DAD with different wavelengths). The same applies to coupling MS or NMR to chromatographic techniques.			✓	
		(b) Quantification of at least two marker compounds (unless this is not feasible, evidence needs to be provided), and justification of the choice of markers (if applicable).			✓	
Use of reference standards	4	(a) Direct overlay of the chromatogram of the sample with that of an officially specified reference standard (if applicable)			✓	
		(b) Chromatographic fingerprinting: Direct overlay of the chromatogram of the sample with that of official reference standards of the powdered plant material or the dry extract from the plant material.				
Comparison of different extracts/ samples of the same plants	5	(a) Direct comparison of the chromatographic/spectroscopic system and/or scoring system for "similarity" to be followed.			✓	

Note: Please also include here the following information about your submitted manuscript:

Name of the journal: Frontiers in Pharmacology

Date of the enquiry: 2026.08.01

Title of the manuscript: Outcomes Reported in Randomized Controlled Trials of Trad

List of the authors: Danni Li

[1] Please acknowledge/cite this as follows: Heinrich M, Jalil B, Abdel-Tawab M, Echeverria J, Kulić Ž, McGaw LJ, et al. Best Practice in the chemical characterisation of extracts used in pharmacological and toxicological research—The ConPhyMP—Guidelines. Frontiers in Pharmacology. 2022;13:953205. https://doi.org/10.3389/fphar.2022.953205

[2] We strongly recommend reading this checklist in conjuncton with ConPhyMP 2022 explanaton and elaboraton for important clarifications on all items. If relevant, we also recommend after reading Heinrich et al. (2020) Best practice in research—Overcoming common challenges in phytopharmacological research. Journal of Ethnopharmacology. 2020;246:112230. https://doi.org/10.1016/j.jep.2019.112230

ConPhyMP checklist of items for conducting and reporting analytical methods[1,2] relevant for extract type B (for species or botanical drugs widely used or traded without a monograph in a national or international pharmacopoeia)

SECTION/TOPIC	ITEM NO.	CHECKLIST ITEM	YES	NO	NOT APPLICABLE	PAGE NO., IF ANY
Type of extract	1	B – Confirm that the species or botanical drug under investigation is a widely used or traded one without a monograph in a national or international pharmacopoeia.	✔	☐	☐	
Preferred/ main methods for extract characterisation/ chemical analysis	2	(a) Triple chemical fingerprinting methods, each with one or more detection parameters.	☐	☐	✔	
		(b) Quantification of at least two marker compounds (unless this is not feasible, evidence needs to be provided), and justification of the choice of markers (if applicable).	☐	☐	✔	
Alternative methods for extract characterisation/chemical analysis	3	(a) Single chemical fingerprinting method, three different detection parameters should be provided (i.e., altered detection parameters, like TLC/HPTLC with different staining reagents and/or UV excitation wavelengths, HPLC-DAD/LC-DAD with different wavelengths). The same applies to coupling MS or NMR to chromatographic techniques.	☐	☐	✔	
		(b) Quantification of at least two marker compounds (unless this is not feasible, evidence needs to be provided), and justification of the choice of markers (if applicable).	☐	☐	✔	
Use of reference standards	4	(a) Direct overlay of the chromatogram of the sample with that of an officially specified reference standard (if applicable)	☐	☐	✔	
		(b) Chromatographic fingerprinting: Direct overlay of the chromatogram of the sample with that of official reference standards of the powdered plant material or the dry extract from the plant material.				
Comparison of different extracts/ samples of the same plants	5	(a) Direct comparison of the chromatographic/spectroscopic system and/or scoring system for "similarity" to be followed.	☐	☐	✔	

Note: Please also include here the following information about your submitted manuscript:

Name of the journal:	Frontiers in Pharmacology
Date of the enquiry:	2026.08.01
Title of the manuscript:	Outcomes Reported in Randomized Controlled Trials of Tra(
List of the authors:	Danni Li

[1] Please acknowledge/cite this as follows: Heinrich M, Jalil B, Abdel-Tawab M, Echeverria J, Kulić Ž, McGaw LJ, et al. Best Practice in the chemical characterisation of extracts used in pharmacological and toxicological research—The ConPhyMP—Guidelines. Frontiers in Pharmacology. 2022;13:953205. https://doi.org/10.3389/fphar.2022.953205

[2] We strongly recommend reading this checklist in conjuncton with ConPhyMP 2022 explanaton and elaboraton for important clarifications on all items. If relevant, we also recommend after reading Heinrich et al. (2020) Best practice in research—Overcoming common challenges in phytopharmacological research. Journal of Ethnopharmacology. 2020;246:112230. https://doi.org/10.1016/j.jep.2019.112230

ConPhyMP checklist of items for conducting and reporting analytical methods[1,2] relevant for extract type B (for species or botanical drugs widely used or traded without a monograph in a national or international pharmacopoeia)

SECTION/TOPIC	ITEM NO.	CHECKLIST ITEM	YES	NO	NOT APPLICABLE	PAGE NO., IF ANY
Type of extract	1	B – Confirm that the species or botanical drug under investigation is a widely used or traded one without a monograph in a national or international pharmacopoeia.	✔			
Preferred/ main methods for extract characterisation/ chemical analysis	2	(a) Triple chemical fingerprinting methods, each with one or more detection parameters.			✔	
		(b) Quantification of at least two marker compounds (unless this is not feasible, evidence needs to be provided), and justification of the choice of markers (if applicable).			✔	
Alternative methods for extract characterisation/chemical analysis	3	(a) Single chemical fingerprinting method, three different detection parameters should be provided (i.e., altered detection parameters, like TLC/HPTLC with different staining reagents and/or UV excitation wavelengths, HPLC-DAD/LC-DAD with different wavelengths). The same applies to coupling MS or NMR to chromatographic techniques.			✔	
		(b) Quantification of at least two marker compounds (unless this is not feasible, evidence needs to be provided), and justification of the choice of markers (if applicable).			✔	
Use of reference standards	4	(a) Direct overlay of the chromatogram of the sample with that of an officially specified reference standard (if applicable)			✔	
		(b) Chromatographic fingerprinting: Direct overlay of the chromatogram of the sample with that of official reference standards of the powdered plant material or the dry extract from the plant material.				
Comparison of different extracts/ samples of the same plants	5	(a) Direct comparison of the chromatographic/spectroscopic system and/or scoring system for "similarity" to be followed.			✔	

Note: Please also include here the following information about your submitted manuscript:

Name of the journal:	Frontiers in Pharmacology
Date of the enquiry:	2026.08.01
Title of the manuscript:	Outcomes Reported in Randomized Controlled Trials of Trac
List of the authors:	Danni Li

[1] Please acknowledge/cite this as follows: Heinrich M, Jalil B, Abdel-Tawab M, Echeverria J, Kulić Ž, McGaw LJ, et al. Best Practice in the chemical characterisation of extracts used in pharmacological and toxicological research—The ConPhyMP—Guidelines. Frontiers in Pharmacology. 2022;13:953205. https://doi.org/10.3389/fphar.2022.953205

[2] We strongly recommend reading this checklist in conjuncton with ConPhyMP 2022 explanaton and elaboraton for important clarifications on all items. If relevant, we also recommend after reading Heinrich et al. (2020) Best practice in research—Overcoming common challenges in phytopharmacological research. Journal of Ethnopharmacology. 2020;246:112230. https://doi.org/10.1016/j.jep.2019.112230

ConPhyMP checklist of items for conducting and reporting analytical methods[1,2] relevant for extract type B (for species or botanical drugs widely used or traded without a monograph in a national or international pharmacopoeia)

SECTION/TOPIC	ITEM NO.	CHECKLIST ITEM	YES	NO	NOT APPLICABLE	PAGE NO., IF ANY
Type of extract	1	B – Confirm that the species or botanical drug under investigation is a widely used or traded one without a monograph in a national or international pharmacopoeia.	✔			
Preferred/ main methods for extract characterisation/ chemical analysis	2	(a) Triple chemical fingerprinting methods, each with one or more detection parameters.			✔	
		(b) Quantification of at least two marker compounds (unless this is not feasible, evidence needs to be provided), and justification of the choice of markers (if applicable).			✔	
Alternative methods for extract characterisation/chemical analysis	3	(a) Single chemical fingerprinting method, three different detection parameters should be provided (i.e., altered detection parameters, like TLC/HPTLC with different staining reagents and/or UV excitation wavelengths, HPLC-DAD/LC-DAD with different wavelengths). The same applies to coupling MS or NMR to chromatographic techniques.			✔	
		(b) Quantification of at least two marker compounds (unless this is not feasible, evidence needs to be provided), and justification of the choice of markers (if applicable).			✔	
Use of reference standards	4	(a) Direct overlay of the chromatogram of the sample with that of an officially specified reference standard (if applicable)			✔	
		(b) Chromatographic fingerprinting: Direct overlay of the chromatogram of the sample with that of official reference standards of the powdered plant material or the dry extract from the plant material.				
Comparison of different extracts/ samples of the same plants	5	(a) Direct comparison of the chromatographic/spectroscopic system and/or scoring system for "similarity" to be followed.			✔	

Note: Please also include here the following information about your submitted manuscript:

Name of the journal:	Frontiers in Pharmacology
Date of the enquiry:	2026.08.01
Title of the manuscript:	Outcomes Reported in Randomized Controlled Trials of Trad
List of the authors:	Danni Li

[1] Please acknowledge/cite this as follows: Heinrich M, Jalil B, Abdel-Tawab M, Echeverria J, Kulić Ž, McGaw LJ, et al. Best Practice in the chemical characterisation of extracts used in pharmacological and toxicological research—The ConPhyMP—Guidelines. Frontiers in Pharmacology. 2022;13:953205. https://doi.org/10.3389/fphar.2022.953205

[2] We strongly recommend reading this checklist in conjuncton with ConPhyMP 2022 explanaton and elaboraton for important clarifications on all items. If relevant, we also recommend after reading Heinrich et al. (2020) Best practice in research—Overcoming common challenges in phytopharmacological research. Journal of Ethnopharmacology. 2020;246:112230. https://doi.org/10.1016/j.jep.2019.112230

ConPhyMP checklist of items for conducting and reporting analytical methods[1,2] relevant for extract type B (for species or botanical drugs widely used or traded without a monograph in a national or international pharmacopoeia)

SECTION/TOPIC	ITEM NO.	CHECKLIST ITEM	YES	NO	NOT APPLICABLE	PAGE NO., IF ANY
Type of extract	1	B – Confirm that the species or botanical drug under investigation is a widely used or traded one without a monograph in a national or international pharmacopoeia.	✔			
Preferred/ main methods for extract characterisation/ chemical analysis	2	(a) Triple chemical fingerprinting methods, each with one or more detection parameters.			✔	
		(b) Quantification of at least two marker compounds (unless this is not feasible, evidence needs to be provided), and justification of the choice of markers (if applicable).			✔	
Alternative methods for extract characterisation/chemical analysis	3	(a) Single chemical fingerprinting method, three different detection parameters should be provided (i.e., altered detection parameters, like TLC/HPTLC with different staining reagents and/or UV excitation wavelengths, HPLC-DAD/LC-DAD with different wavelengths). The same applies to coupling MS or NMR to chromatographic techniques.			✔	
		(b) Quantification of at least two marker compounds (unless this is not feasible, evidence needs to be provided), and justification of the choice of markers (if applicable).			✔	
Use of reference standards	4	(a) Direct overlay of the chromatogram of the sample with that of an officially specified reference standard (if applicable)			✔	
		(b) Chromatographic fingerprinting: Direct overlay of the chromatogram of the sample with that of official reference standards of the powdered plant material or the dry extract from the plant material.				
Comparison of different extracts/ samples of the same plants	5	(a) Direct comparison of the chromatographic/spectroscopic system and/or scoring system for "similarity" to be followed.			✔	

Note: Please also include here the following information about your submitted manuscript:

Name of the journal:	Frontiers in Pharmacology
Date of the enquiry:	2026.08.01
Title of the manuscript:	Outcomes Reported in Randomized Controlled Trials of Trad
List of the authors:	Danni Li

[1] Please acknowledge/cite this as follows: Heinrich M, Jalil B, Abdel-Tawab M, Echeverria J, Kulić Ž, McGaw LJ, et al. Best Practice in the chemical characterisation of extracts used in pharmacological and toxicological research—The ConPhyMP—Guidelines. Frontiers in Pharmacology. 2022;13:953205. https://doi.org/10.3389/fphar.2022.953205

[2] We strongly recommend reading this checklist in conjuncton with ConPhyMP 2022 explanaton and elaboraton for important clarifications on all items. If relevant, we also recommend after reading Heinrich et al. (2020) Best practice in research—Overcoming common challenges in phytopharmacological research. Journal of Ethnopharmacology. 2020;246:112230. https://doi.org/10.1016/j.jep.2019.112230

ConPhyMP checklist of items for conducting and reporting analytical methods[1,2] relevant for extract type B (for species or botanical drugs widely used or traded without a monograph in a national or international pharmacopoeia)

SECTION/TOPIC	ITEM NO.	CHECKLIST ITEM	YES	NO	NOT APPLICABLE	PAGE NO., IF ANY
Type of extract	1	B – Confirm that the species or botanical drug under investigation is a widely used or traded one without a monograph in a national or international pharmacopoeia.	✔			
Preferred/ main methods for extract characterisation/ chemical analysis	2	(a) Triple chemical fingerprinting methods, each with one or more detection parameters.			✔	
		(b) Quantification of at least two marker compounds (unless this is not feasible, evidence needs to be provided), and justification of the choice of markers (if applicable).			✔	
Alternative methods for extract characterisation/chemical analysis	3	(a) Single chemical fingerprinting method, three different detection parameters should be provided (i.e., altered detection parameters, like TLC/HPTLC with different staining reagents and/or UV excitation wavelengths, HPLC-DAD/LC-DAD with different wavelengths). The same applies to coupling MS or NMR to chromatographic techniques.			✔	
		(b) Quantification of at least two marker compounds (unless this is not feasible, evidence needs to be provided), and justification of the choice of markers (if applicable).			✔	
Use of reference standards	4	(a) Direct overlay of the chromatogram of the sample with that of an officially specified reference standard (if applicable)			✔	
		(b) Chromatographic fingerprinting: Direct overlay of the chromatogram of the sample with that of official reference standards of the powdered plant material or the dry extract from the plant material.				
Comparison of different extracts/ samples of the same plants	5	(a) Direct comparison of the chromatographic/spectroscopic system and/or scoring system for "similarity" to be followed.			✔	

Note: Please also include here the following information about your submitted manuscript:

Name of the journal:	Frontiers in Pharmacology
Date of the enquiry:	2026.08.01
Title of the manuscript:	Outcomes Reported in Randomized Controlled Trials of Tra
List of the authors:	Danni Li

[1] Please acknowledge/cite this as follows: Heinrich M, Jalil B, Abdel-Tawab M, Echeverria J, Kulić Ž, McGaw LJ, et al. Best Practice in the chemical characterisation of extracts used in pharmacological and toxicological research—The ConPhyMP—Guidelines. Frontiers in Pharmacology. 2022;13:953205. https://doi.org/10.3389/fphar.2022.953205

[2] We strongly recommend reading this checklist in conjuncton with ConPhyMP 2022 explanaton and elaboraton for important clarifications on all items. If relevant, we also recommend after reading Heinrich et al. (2020) Best practice in research—Overcoming common challenges in phytopharmacological research. Journal of Ethnopharmacology. 2020;246:112230. https://doi.org/10.1016/j.jep.2019.112230

ConPhyMP checklist of items for conducting and reporting analytical methods[1,2] relevant for extract type B (for species or botanical drugs widely used or traded without a monograph in a national or international pharmacopoeia)

SECTION/TOPIC	ITEM NO.	CHECKLIST ITEM	YES	NO	NOT APPLICABLE	PAGE NO., IF ANY
Type of extract	1	B – Confirm that the species or botanical drug under investigation is a widely used or traded one without a monograph in a national or international pharmacopoeia.	✔			
Preferred/ main methods for extract characterisation/ chemical analysis	2	(a) Triple chemical fingerprinting methods, each with one or more detection parameters.			✔	
		(b) Quantification of at least two marker compounds (unless this is not feasible, evidence needs to be provided), and justification of the choice of markers (if applicable).			✔	
Alternative methods for extract characterisation/chemical analysis	3	(a) Single chemical fingerprinting method, three different detection parameters should be provided (i.e., altered detection parameters, like TLC/HPTLC with different staining reagents and/or UV excitation wavelengths, HPLC-DAD/LC-DAD with different wavelengths). The same applies to coupling MS or NMR to chromatographic techniques.			✔	
		(b) Quantification of at least two marker compounds (unless this is not feasible, evidence needs to be provided), and justification of the choice of markers (if applicable).			✔	
Use of reference standards	4	(a) Direct overlay of the chromatogram of the sample with that of an officially specified reference standard (if applicable)			✔	
		(b) Chromatographic fingerprinting: Direct overlay of the chromatogram of the sample with that of official reference standards of the powdered plant material or the dry extract from the plant material.				
Comparison of different extracts/ samples of the same plants	5	(a) Direct comparison of the chromatographic/spectroscopic system and/or scoring system for "similarity" to be followed.			✔	

Note: Please also include here the following information about your submitted manuscript:

Name of the journal: Frontiers in Pharmacology

Date of the enquiry: 2026.08.01

Title of the manuscript: Outcomes Reported in Randomized Controlled Trials of Tra

List of the authors: Danni Li

[1] Please acknowledge/cite this as follows: Heinrich M, Jalil B, Abdel-Tawab M, Echeverria J, Kulić Ž, McGaw LJ, et al. Best Practice in the chemical characterisation of extracts used in pharmacological and toxicological research—The ConPhyMP—Guidelines. Frontiers in Pharmacology. 2022;13:953205. https://doi.org/10.3389/fphar.2022.953205

[2] We strongly recommend reading this checklist in conjuncton with ConPhyMP 2022 explanaton and elaboraton for important clarifications on all items. If relevant, we also recommend after reading Heinrich et al. (2020) Best practice in research—Overcoming common challenges in phytopharmacological research. Journal of Ethnopharmacology. 2020;246:112230. https://doi.org/10.1016/j.jep.2019.112230

ConPhyMP checklist of items for conducting and reporting analytical methods[1,2] relevant for extract type B (for species or botanical drugs widely used or traded without a monograph in a national or international pharmacopoeia)

SECTION/TOPIC	ITEM NO.	CHECKLIST ITEM	YES	NO	NOT APPLICABLE	PAGE NO., IF ANY
Type of extract	1	B – Confirm that the species or botanical drug under investigation is a widely used or traded one without a monograph in a national or international pharmacopoeia.	✔			
Preferred/ main methods for extract characterisation/ chemical analysis	2	(a) Triple chemical fingerprinting methods, each with one or more detection parameters.			✔	
		(b) Quantification of at least two marker compounds (unless this is not feasible, evidence needs to be provided), and justification of the choice of markers (if applicable).			✔	
Alternative methods for extract characterisation/chemical analysis	3	(a) Single chemical fingerprinting method, three different detection parameters should be provided (i.e., altered detection parameters, like TLC/HPTLC with different staining reagents and/or UV excitation wavelengths, HPLC-DAD/LC-DAD with different wavelengths). The same applies to coupling MS or NMR to chromatographic techniques.			✔	
		(b) Quantification of at least two marker compounds (unless this is not feasible, evidence needs to be provided), and justification of the choice of markers (if applicable).			✔	
Use of reference standards	4	(a) Direct overlay of the chromatogram of the sample with that of an officially specified reference standard (if applicable)			✔	
		(b) Chromatographic fingerprinting: Direct overlay of the chromatogram of the sample with that of official reference standards of the powdered plant material or the dry extract from the plant material.				
Comparison of different extracts/ samples of the same plants	5	(a) Direct comparison of the chromatographic/spectroscopic system and/or scoring system for "similarity" to be followed.			✔	

Note: Please also include here the following information about your submitted manuscript:

Name of the journal:	Frontiers in Pharmacology
Date of the enquiry:	2026.08.01
Title of the manuscript:	Outcomes Reported in Randomized Controlled Trials of Trad
List of the authors:	Danni Li

[1] Please acknowledge/cite this as follows: Heinrich M, Jalil B, Abdel-Tawab M, Echeverria J, Kulić Ž, McGaw LJ, et al. Best Practice in the chemical characterisation of extracts used in pharmacological and toxicological research—The ConPhyMP—Guidelines. Frontiers in Pharmacology. 2022;13:953205. https://doi.org/10.3389/fphar.2022.953205

[2] We strongly recommend reading this checklist in conjuncton with ConPhyMP 2022 explanaton and elaboraton for important clarifications on all items. If relevant, we also recommend after reading Heinrich et al. (2020) Best practice in research—Overcoming common challenges in phytopharmacological research. Journal of Ethnopharmacology. 2020;246:112230. https://doi.org/10.1016/j.jep.2019.112230

ConPhyMP checklist of items for conducting and reporting analytical methods[1,2] relevant for extract type B (for species or botanical drugs widely used or traded without a monograph in a national or international pharmacopoeia)

SECTION/TOPIC	ITEM NO.	CHECKLIST ITEM	YES	NO	NOT APPLICABLE	PAGE NO., IF ANY
Type of extract	1	B – Confirm that the species or botanical drug under investigation is a widely used or traded one without a monograph in a national or international pharmacopoeia.	✔			
Preferred/ main methods for extract characterisation/ chemical analysis	2	(a) Triple chemical fingerprinting methods, each with one or more detection parameters.			✔	
		(b) Quantification of at least two marker compounds (unless this is not feasible, evidence needs to be provided), and justification of the choice of markers (if applicable).			✔	
Alternative methods for extract characterisation/chemical analysis	3	(a) Single chemical fingerprinting method, three different detection parameters should be provided (i.e., altered detection parameters, like TLC/HPTLC with different staining reagents and/or UV excitation wavelengths, HPLC-DAD/LC-DAD with different wavelengths). The same applies to coupling MS or NMR to chromatographic techniques.			✔	
		(b) Quantification of at least two marker compounds (unless this is not feasible, evidence needs to be provided), and justification of the choice of markers (if applicable).			✔	
Use of reference standards	4	(a) Direct overlay of the chromatogram of the sample with that of an officially specified reference standard (if applicable)			✔	
		(b) Chromatographic fingerprinting: Direct overlay of the chromatogram of the sample with that of official reference standards of the powdered plant material or the dry extract from the plant material.				
Comparison of different extracts/ samples of the same plants	5	(a) Direct comparison of the chromatographic/spectroscopic system and/or scoring system for "similarity" to be followed.			✔	

Note: Please also include here the following information about your submitted manuscript:

Name of the journal:	Frontiers in Pharmacology
Date of the enquiry:	2026.08.01
Title of the manuscript:	Outcomes Reported in Randomized Controlled Trials of Trac
List of the authors:	Danni Li

[1] Please acknowledge/cite this as follows: Heinrich M, Jalil B, Abdel-Tawab M, Echeverria J, Kulić Ž, McGaw LJ, et al. Best Practice in the chemical characterisation of extracts used in pharmacological and toxicological research—The ConPhyMP—Guidelines. Frontiers in Pharmacology. 2022;13:953205. https://doi.org/10.3389/fphar.2022.953205

[2] We strongly recommend reading this checklist in conjuncton with ConPhyMP 2022 explanaton and elaboraton for important clarifications on all items. If relevant, we also recommend after reading Heinrich et al. (2020) Best practice in research—Overcoming common challenges in phytopharmacological research. Journal of Ethnopharmacology. 2020;246:112230. https://doi.org/10.1016/j.jep.2019.112230

ConPhyMP checklist of items for conducting and reporting analytical methods[1,2] relevant for extract type B (for species or botanical drugs widely used or traded without a monograph in a national or international pharmacopoeia)

SECTION/TOPIC	ITEM NO.	CHECKLIST ITEM	YES	NO	NOT APPLICABLE	PAGE NO., IF ANY
Type of extract	1	B – Confirm that the species or botanical drug under investigation is a widely used or traded one without a monograph in a national or international pharmacopoeia.	✔			
Preferred/ main methods for extract characterisation/ chemical analysis	2	(a) Triple chemical fingerprinting methods, each with one or more detection parameters.			✔	
		(b) Quantification of at least two marker compounds (unless this is not feasible, evidence needs to be provided), and justification of the choice of markers (if applicable).			✔	
Alternative methods for extract characterisation/chemical analysis	3	(a) Single chemical fingerprinting method, three different detection parameters should be provided (i.e., altered detection parameters, like TLC/HPTLC with different staining reagents and/or UV excitation wavelengths, HPLC-DAD/LC-DAD with different wavelengths). The same applies to coupling MS or NMR to chromatographic techniques.			✔	
		(b) Quantification of at least two marker compounds (unless this is not feasible, evidence needs to be provided), and justification of the choice of markers (if applicable).			✔	
Use of reference standards	4	(a) Direct overlay of the chromatogram of the sample with that of an officially specified reference standard (if applicable)			✔	
		(b) Chromatographic fingerprinting: Direct overlay of the chromatogram of the sample with that of official reference standards of the powdered plant material or the dry extract from the plant material.				
Comparison of different extracts/ samples of the same plants	5	(a) Direct comparison of the chromatographic/spectroscopic system and/or scoring system for "similarity" to be followed.			✔	

Note: Please also include here the following information about your submitted manuscript:

Name of the journal:	Frontiers in Pharmacology
Date of the enquiry:	2026.08.01
Title of the manuscript:	Outcomes Reported in Randomized Controlled Trials of Trac
List of the authors:	Danni Li

[1] Please acknowledge/cite this as follows: Heinrich M, Jalil B, Abdel-Tawab M, Echeverria J, Kulić Ž, McGaw LJ, et al. Best Practice in the chemical characterisation of extracts used in pharmacological and toxicological research—The ConPhyMP—Guidelines. Frontiers in Pharmacology. 2022;13:953205. https://doi.org/10.3389/fphar.2022.953205

[2] We strongly recommend reading this checklist in conjuncton with ConPhyMP 2022 explanaton and elaboraton for important clarifications on all items. If relevant, we also recommend after reading Heinrich et al. (2020) Best practice in research—Overcoming common challenges in phytopharmacological research. Journal of Ethnopharmacology. 2020;246:112230. https://doi.org/10.1016/j.jep.2019.112230

ConPhyMP checklist of items for conducting and reporting analytical methods[1,2] relevant for extract type B (for species or botanical drugs widely used or traded without a monograph in a national or international pharmacopoeia)

SECTION/TOPIC	ITEM NO.	CHECKLIST ITEM	YES	NO	NOT APPLICABLE	PAGE NO., IF ANY
Type of extract	1	B – Confirm that the species or botanical drug under investigation is a widely used or traded one without a monograph in a national or international pharmacopoeia.	✔			
Preferred/ main methods for extract characterisation/ chemical analysis	2	(a) Triple chemical fingerprinting methods, each with one or more detection parameters.			✔	
		(b) Quantification of at least two marker compounds (unless this is not feasible, evidence needs to be provided), and justification of the choice of markers (if applicable).			✔	
Alternative methods for extract characterisation/chemical analysis	3	(a) Single chemical fingerprinting method, three different detection parameters should be provided (i.e., altered detection parameters, like TLC/HPTLC with different staining reagents and/or UV excitation wavelengths, HPLC-DAD/LC-DAD with different wavelengths). The same applies to coupling MS or NMR to chromatographic techniques.			✔	
		(b) Quantification of at least two marker compounds (unless this is not feasible, evidence needs to be provided), and justification of the choice of markers (if applicable).			✔	
Use of reference standards	4	(a) Direct overlay of the chromatogram of the sample with that of an officially specified reference standard (if applicable)			✔	
		(b) Chromatographic fingerprinting: Direct overlay of the chromatogram of the sample with that of official reference standards of the powdered plant material or the dry extract from the plant material.				
Comparison of different extracts/ samples of the same plants	5	(a) Direct comparison of the chromatographic/spectroscopic system and/or scoring system for "similarity" to be followed.			✔	

Note: Please also include here the following information about your submitted manuscript:

Name of the journal:	Frontiers in Pharmacology
Date of the enquiry:	2026.08.01
Title of the manuscript:	Outcomes Reported in Randomized Controlled Trials of Trad
List of the authors:	Danni Li

[1] Please acknowledge/cite this as follows: Heinrich M, Jalil B, Abdel-Tawab M, Echeverria J, Kulić Ž, McGaw LJ, et al. Best Practice in the chemical characterisation of extracts used in pharmacological and toxicological research—The ConPhyMP—Guidelines. Frontiers in Pharmacology. 2022;13:953205. https://doi.org/10.3389/fphar.2022.953205

[2] We strongly recommend reading this checklist in conjuncton with ConPhyMP 2022 explanaton and elaboraton for important clarifications on all items. If relevant, we also recommend after reading Heinrich et al. (2020) Best practice in research—Overcoming common challenges in phytopharmacological research. Journal of Ethnopharmacology. 2020;246:112230. https://doi.org/10.1016/j.jep.2019.112230

ConPhyMP checklist of items for conducting and reporting analytical methods[1,2] relevant for extract type B (for species or botanical drugs widely used or traded without a monograph in a national or international pharmacopoeia)

SECTION/TOPIC	ITEM NO.	CHECKLIST ITEM	YES	NO	NOT APPLICABLE	PAGE NO., IF ANY
Type of extract	1	B – Confirm that the species or botanical drug under investigation is a widely used or traded one without a monograph in a national or international pharmacopoeia.	✔			
Preferred/ main methods for extract characterisation/ chemical analysis	2	(a) Triple chemical fingerprinting methods, each with one or more detection parameters.			✔	
		(b) Quantification of at least two marker compounds (unless this is not feasible, evidence needs to be provided), and justification of the choice of markers (if applicable).			✔	
Alternative methods for extract characterisation/chemical analysis	3	(a) Single chemical fingerprinting method, three different detection parameters should be provided (i.e., altered detection parameters, like TLC/HPTLC with different staining reagents and/or UV excitation wavelengths, HPLC-DAD/LC-DAD with different wavelengths). The same applies to coupling MS or NMR to chromatographic techniques.			✔	
		(b) Quantification of at least two marker compounds (unless this is not feasible, evidence needs to be provided), and justification of the choice of markers (if applicable).			✔	
Use of reference standards	4	(a) Direct overlay of the chromatogram of the sample with that of an officially specified reference standard (if applicable)			✔	
		(b) Chromatographic fingerprinting: Direct overlay of the chromatogram of the sample with that of official reference standards of the powdered plant material or the dry extract from the plant material.				
Comparison of different extracts/ samples of the same plants	5	(a) Direct comparison of the chromatographic/spectroscopic system and/or scoring system for "similarity" to be followed.			✔	

Note: Please also include here the following information about your submitted manuscript:

Name of the journal:	Frontiers in Pharmacology
Date of the enquiry:	2026.08.01
Title of the manuscript:	Outcomes Reported in Randomized Controlled Trials of Trac
List of the authors:	Danni Li

[1] Please acknowledge/cite this as follows: Heinrich M, Jalil B, Abdel-Tawab M, Echeverria J, Kulić Ž, McGaw LJ, et al. Best Practice in the chemical characterisation of extracts used in pharmacological and toxicological research—The ConPhyMP—Guidelines. Frontiers in Pharmacology. 2022;13:953205. https://doi.org/10.3389/fphar.2022.953205

[2] We strongly recommend reading this checklist in conjuncton with ConPhyMP 2022 explanaton and elaboraton for important clarifications on all items. If relevant, we also recommend after reading Heinrich et al. (2020) Best practice in research—Overcoming common challenges in phytopharmacological research. Journal of Ethnopharmacology. 2020;246:112230. https://doi.org/10.1016/j.jep.2019.112230

ConPhyMP checklist of items for conducting and reporting analytical methods[1,2] relevant for extract type B (for species or botanical drugs widely used or traded without a monograph in a national or international pharmacopoeia)

SECTION/TOPIC	ITEM NO.	CHECKLIST ITEM	YES	NO	NOT APPLICABLE	PAGE NO., IF ANY
Type of extract	1	B – Confirm that the species or botanical drug under investigation is a widely used or traded one without a monograph in a national or international pharmacopoeia.	✔			
Preferred/ main methods for extract characterisation/ chemical analysis	2	(a) Triple chemical fingerprinting methods, each with one or more detection parameters.			✔	
		(b) Quantification of at least two marker compounds (unless this is not feasible, evidence needs to be provided), and justification of the choice of markers (if applicable).			✔	
Alternative methods for extract characterisation/chemical analysis	3	(a) Single chemical fingerprinting method, three different detection parameters should be provided (i.e., altered detection parameters, like TLC/HPTLC with different staining reagents and/or UV excitation wavelengths, HPLC-DAD/LC-DAD with different wavelengths). The same applies to coupling MS or NMR to chromatographic techniques.			✔	
		(b) Quantification of at least two marker compounds (unless this is not feasible, evidence needs to be provided), and justification of the choice of markers (if applicable).			✔	
Use of reference standards	4	(a) Direct overlay of the chromatogram of the sample with that of an officially specified reference standard (if applicable)			✔	
		(b) Chromatographic fingerprinting: Direct overlay of the chromatogram of the sample with that of official reference standards of the powdered plant material or the dry extract from the plant material.				
Comparison of different extracts/ samples of the same plants	5	(a) Direct comparison of the chromatographic/spectroscopic system and/or scoring system for "similarity" to be followed.			✔	

Note: Please also include here the following information about your submitted manuscript:

Name of the journal:	Frontiers in Pharmacology
Date of the enquiry:	2026.08.01
Title of the manuscript:	Outcomes Reported in Randomized Controlled Trials of Trad
List of the authors:	Danni Li

[1] Please acknowledge/cite this as follows: Heinrich M, Jalil B, Abdel-Tawab M, Echeverria J, Kulić Ž, McGaw LJ, et al. Best Practice in the chemical characterisation of extracts used in pharmacological and toxicological research—The ConPhyMP—Guidelines. Frontiers in Pharmacology. 2022;13:953205. https://doi.org/10.3389/fphar.2022.953205

[2] We strongly recommend reading this checklist in conjuncton with ConPhyMP 2022 explanaton and elaboraton for important clarifications on all items. If relevant, we also recommend after reading Heinrich et al. (2020) Best practice in research—Overcoming common challenges in phytopharmacological research. Journal of Ethnopharmacology. 2020;246:112230. https://doi.org/10.1016/j.jep.2019.112230

ConPhyMP checklist of information for reporting plant material and its initial processing [1,2] (relevant for all studies on medicinal and food plants including extract types A, B, and C)

SECTION/TOPIC	ITEM NO.	CHECKLIST ITEM	YES	NO	NOT APPLICABLE	PAGE NO., IF ANY
Title and abstract	1	A clear and concise title including an informative abstract and balanced summary.	✔			
Description of the botanical drug and taxonomic authentication	2	Botanical or morphological authentication of the plant material (desirable is a combination with DNA barcoding, e.g., PCR, RFLP, genome sequencing) and the information must be included in a separate section of Material and Methods, if applicable, combined with the information required under item 3:			✔	
Description of the extract and extraction process	3	A separate section in Material and Methods, covers the relevant information on the material investigated, including the full species name(s), authorites and family; e.g. *Salvia miltorrhiza* Bunge [Lamiaceae; *Salviae miltorrhizae radix et rhizoma*], and on the processing and extraction of the crude drug including the traditional processing of the material used medicinally (fumigation, steaming, roasting, cooking, frying, etc.).			✔	
Documentation of the legal basis for collection and processing	4	Full compliance with the Nagoya protocol, CITES, and all associated treaties including phytosanitary regulations.			✔	
Description of product characteristics, in case of a finished (commercial) product	5	Information on the characteristics of the commercial products including batch number and date of production/best by information and regulatory status.	✔			

Note: Please also include here the following information about your submitted manuscript:

Name of the journal:	Frontiers in Pharmacology
Date of the enquiry:	2026.08.01
Title of the manuscript:	Outcomes Reported in Randomized Controlled Trials of Tra
List of the authors:	Danni Li

[1] Please acknowledge/cite this as follows: Heinrich M, Jalil B, Abdel-Tawab M, Echeverria J, Kulić Ž, McGaw LJ, et al. Best Practice in the chemical characterisation of extracts used in pharmacological and toxicological research—The ConPhyMP—Guidelines. Frontiers in Pharmacology. 2022;13:953205. https://doi.org/10.3389/fphar.2022.953205

[2] We strongly recommend reading this checklist in conjuncton with ConPhyMP 2022 explanaton and elaboraton for important clarifications on all items. If relevant, we also recommend after reading Heinrich et al. (2020) Best practice in research—Overcoming common challenges in phytopharmacological research. Journal of Ethnopharmacology. 2020;246:112230. https://doi.org/10.1016/j.jep.2019.112230
