# Supplementary material for: Outcomes reported in randomized controlled trials of traditional Chinese medicine for chronic pharyngitis: a scoping review
Source: Front Pharmacol. 2026 Oct 1;17:1958608. doi: 10.3389/fphar.2026.1958608 (PMC13669160; doi:10.3389/fphar.2026.1958608)
Supplement: Supplementary file 3 [file Table1.docx]

**Appendix A1. Composition and characterization of the traditional Chinese medicine preparations identified in the included trials**

| **No.** | **Intervention reported in the included literature** | **Standardized preparation** | **Dosage form/type** | **Standardized constituent drugs** |
| --- | --- | --- | --- | --- |
| 1 | Nourishing Yin Clearing Lung Soup | Yangyin Qingfei Decoction | Classical decoction | Dihuang; Maidong; Xuanshen; Beimu; Mudanpi; Bohe; Baishao; Gancao |
| 2 | Jinsang Liyan Pills | Jinsang Liyan Pills | Proprietary pills | Fuling; Zhishi; Dannanxing; Sharen; Binglang; Liushenqu; Shengjiang; Muhudie; Fabanxia; Qingpi; Juhong; Doukou; Hehuanpi; Zisugeng; Chantui; Houpo |
| 3 | Lanqin oral solution | Lanqin Oral Liquid | Proprietary oral liquid | Banlangen; Huangqin; Zhizi; Huangbo; Pangdahai |
| 4 | Pinellia Houpu Soup | Banxia Houpu Decoction | Classical decoction | Banxia; Houpo; Fuling; Shengjiang; Zisuye |
| 5 | Andrographolide Dropping Pills | Andrographolide Dropping Pills | Purified-compound preparation | Andrographolide |
| 6 | Houyanqing Granules | Houyanqing Granules | Proprietary granules | Tuniuxi; Malancao; Cheqiancao; Tianmingjing |
| 7 | Qianyang Fengsui Dan | Qianyang Fengsui Dan | Classical combined formula | Sharen; Fuzi; Guijia/Guiban; Gancao; Huangbo |
| 8 | Yangyin Liyan Decoction | Yangyin Liyan Decoction | Classical/experienced decoction | Nanshashen; Beishashen; Baihe; Baishao; Tianhuafen; Shegan; Jiegeng; Gancao |
| 9 | Maintain Yin Relieving Sore-Throat Decoction | Ziyin Huoxue Liyan Decoction | Experienced decoction | Maidong; Danshen; Xuanshen; Chuanxiong; Chishao; Niubangzi; Jiegeng; Chantui; Chaihu; Zhiqiao; Gancao |
| 10 | Yiqing Capsule | Yiqing Capsule | Proprietary capsule | Huanglian; Dahuang; Huangqin |
| 11 | Epiglottis Zhuyu Decoction | Huiyan Zhuyu Decoction | Classical decoction | Taoren; Honghua; Gancao; Jiegeng; Dihuang; Danggui; Xuanshen; Chaihu; Zhiqiao; Chishao |
| 12 | Stomatitis Granules | Kouyanqing Granules | Proprietary granules | Tiandong; Maidong; Xuanshen; Shanyinhua; Gancao |
| 13 | Shashen Maidong Decoction | Shashen Maidong Decoction | Classical decoction | Shashen; Yuzhu; Gancao; Sangye; Maidong; Baibiandou; Tianhuafen |
| 14 | Tianshenliyan particles | Tianshen Liyan Granules | Hospital-prepared granules | Nanshashen; Beishashen; Baihe; Baishao; Tianhuafen; Shegan; Jiegeng; Gancao |
| 15 | Atomizing agent for Tianzhu | Tianzhu Atomizing Agent | Herbal nebulization preparation | Tianzhuhuang; Liangmianzhen; Gualoupi; Qiannianjian; Jiangcan; Bohe; Yuxingcao; Muxiang; Tinglizi |
| 16 | Xinhuang Tablets | Xinhuang Tablets | Chinese-Western combination tablet | Zhongjiefeng; Sanqi; artificial Calculus Bovis; porcine bile powder; Xiaofantianhua; nacre powder; concentrated buffalo horn powder; Hongqu; indomethacin |
| 17 | Decoction of Lilii for Strengthening Lung | Baihe Gujin Decoction | Classical decoction | Shudihuang; Dihuang; Danggui; Baishao; Gancao; Jiegeng; Xuanshen; Beimu; Maidong; Baihe |
| 18 | Banlangen Granule | Banlangen Granules | Single-drug granules | Banlangen |
| 19 | Powder of Fritillary Bulb and Snakegourd | Beimu Gualou Powder | Classical powder/decoction | Beimu; Gualou; Tianhuafen; Fuling; Juhong; Jiegeng |
| 20 | Powder of Ginseng, Poria and Atractylodis macrocephalae | Shenling Baizhu Powder | Classical powder | Renshen; Fuling; Baizhu; Shanyao; Baibiandou; Lianzi; Yiyiren; Sharen; Jiegeng; Gancao |
| 21 | Shenmaiyanshu Oral Liquid | Shenmai Yanshu Oral Liquid | Compound oral liquid | Renshen; Maidong; Shanyao; Qingbanxia; Niubangzi; Zisuzi; Baishao; Gancao; Huangqin |
| 22 | Danshen Injection | Danshen Injection | Injectable botanical extract | Danshen |
| 23 | Compound Huangjing Decoction | Compound Huangjing Decoction | Compound decoction | Huangjing; Shashen; Maidong; Chantui; Cijili; Jiangcan; Mudanpi; Chishao; Baizhu; Haizao; Fabanxia; Haigefen; Huangqi |
| 24 | Compound Bamboo Leaf Gypsum Granules | Compound Zhuye Shigao Granules | Compound granules | Danzhuye; Shigao; Maidong; Renshen; Fabanxia; Jingmi; Zhigancao |
| 25 | Bingmei slices of kumquat | Ganjie Bingmei Tablets | Proprietary tablets | Jiegeng; Bohe; Shegan; Chantui; Wumei; Bingpian; Gancao; Qingguo |
| 26 | Jinguo Drink | Jinguoyin | Proprietary oral liquid | Dihuang; Xuanshen; Xiqingguo; Chantui; Maidong; Pangdahai; Nanshashen; Taizishen; Chenpi; peppermint oil |
| 27 | Liyan Oral Liquid | Liyan Oral Liquid | Compound oral liquid | Jinyinhua; Huangqin; Jingjie; Zhizi; Lianqiao; Xuanshen; Jiangcan; Dihuang; Shegan; Jiegeng; Bohe; Chantui; Fangfeng; Gancao |
| 28 | Decoction of Ophiopogonis | Maimendong Decoction | Classical decoction | Maidong; Banxia; Renshen; Gancao; Jingmi; Dazao |
| 29 | Qingyan Huajie Decoction | Qingyan Huajie Decoction | Experienced decoction | Huangqi; Dangshen; Fabanxia; Chaobaizhu; Danshen; Fuling; Shegan; Zisuzi; Houpo; Jiangcan; Zhigancao; Fangfeng |
| 30 | Shiwei Longdanhua Granules | Shiwei Longdanhua Granules | Proprietary Tibetan-medicine granules | Longdanhua; Liexiangdujuan; Chuanbeimu; Gancao; Aizijin; Xiaobopi; Zangmuxiang; Pangxiejia; Jidanshen; Maniaopao |
| 31 | Tiedi Buccal Tablets | Tiedi Tablets | Proprietary tablets | Maidong; Xuanshen; Gualoupi; Hezi; Qingguo; Fenghuangyi; Jiegeng; Zhebeimu; Gancao |
| 32 | Watermelon Frost Lozenges | Xiguashuang Runhou Tablets | Proprietary lozenge/tablet | Xiguashuang; Bingpian; peppermint oil; menthol |
| 33 | Xiaoyao Powder | Xiaoyao Powder | Classical powder/decoction | Chaihu; Danggui; Baishao; Baizhu; Fuling; Gancao; Shengjiang; Bohe |
| 34 | Yanlishu Oral Liquid | Yanshu Oral Liquid | Proprietary oral liquid | Huzhangcao; Wuxiangcao; Xuanshen; Shegan; Niubangzi; Jiegeng; Chenpi; Gancao |
| 35 | Silver Yellow Particles | Yinhuang Granules | Proprietary extract granules | Jinyinhua extract; Huangqin extract |
| 36 | Yuxingcao Injection | Yuxingcao Injection | Injectable botanical preparation | Yuxingcao |
| 37 | Zhi Bai Di Huang | Zhibai Dihuang Pills | Classical/proprietary pills | Zhimu; Huangbo; Shudihuang; Shanzhuyu; Mudanpi; Shanyao; Fuling; Zexie |
