# Supplementary material for: Outcomes reported in randomized controlled trials of traditional Chinese medicine for chronic pharyngitis: a scoping review
Source: Front Pharmacol. 2026 Oct 1;17:1958608. doi: 10.3389/fphar.2026.1958608 (PMC13669160; doi:10.3389/fphar.2026.1958608)
Supplement: Supplementary file 4 [file Table2.docx]

**Appendix A2.1.Taxonomic and pharmacopoeial standardization of medicinal drugs identified in the reviewed TCM preparations**--**Vascular botanical drugs**

| **Medicinal drug** | **Standardized botanical and pharmacopoeial presentation** |
| --- | --- |
| Dihuang / Sheng Dihuang | *Rehmannia glutinosa* (Gaertn.) Libosch. ex DC. [Orobanchaceae; Rehmanniae radix] |
| Shudihuang | *Rehmannia glutinosa* (Gaertn.) Libosch. ex DC. [Orobanchaceae; Rehmanniae radix praeparata] |
| Maidong | *Ophiopogon japonicus* (Thunb.) Ker Gawl. [Asparagaceae; Ophiopogonis radix] |
| Xuanshen | *Scrophularia ningpoensis* Hemsl. [Scrophulariaceae; Scrophulariae radix] |
| Beimu | *Fritillaria cirrhosa* D.Don; *Fritillaria unibracteata* P.K.Hsiao & K.C.Hsia; *Fritillaria przewalskii* Maxim.; *Fritillaria delavayi* Franch.; *Fritillaria taipaiensis* P.Y.Li; *Fritillaria unibracteata* var. *wabuensis* (S.Y.Tang & S.C.Yueh) Z.D.Liu, Shu Wang & S.C.Chen [Liliaceae; Fritillariae cirrhosae bulbus]; and *Fritillaria thunbergii* Miq. [Liliaceae; Fritillariae thunbergii bulbus] |
| Chuanbeimu | *Fritillaria cirrhosa* D.Don; *Fritillaria unibracteata* P.K.Hsiao & K.C.Hsia; *Fritillaria przewalskii* Maxim.; *Fritillaria delavayi* Franch.; *Fritillaria taipaiensis* P.Y.Li; *Fritillaria unibracteata* var. *wabuensis* (S.Y.Tang & S.C.Yueh) Z.D.Liu, Shu Wang & S.C.Chen [Liliaceae; Fritillariae cirrhosae bulbus] |
| Zhebeimu | *Fritillaria thunbergii* Miq. [Liliaceae; Fritillariae thunbergii bulbus] |
| Mudanpi | *Paeonia × suffruticosa* Andrews [Paeoniaceae; Moutan cortex] |
| Bohe | *Mentha canadensis* L. [Lamiaceae; Menthae haplocalycis herba]; pharmacopoeial botanical name traditionally cited as *Mentha haplocalyx* Briq. |
| Baishao / Shengbaishao | *Paeonia lactiflora* Pall. [Paeoniaceae; Paeoniae radix alba] |
| Chishao | *Paeonia lactiflora* Pall.; *Paeonia veitchii* Lynch [Paeoniaceae; Paeoniae radix rubra] |
| Gancao / Zhigancao | *Glycyrrhiza uralensis* Fisch. ex DC.; *Glycyrrhiza inflata* Batalin; *Glycyrrhiza glabra* L. [Fabaceae; Glycyrrhizae radix et rhizoma] |
| Zhishi | *Citrus × aurantium* L. [Rutaceae; Aurantii fructus immaturus] |
| Zhiqiao | *Citrus × aurantium* L. [Rutaceae; Aurantii fructus] |
| Dannanxing | *Arisaema erubescens* (Wall.) Schott; *Arisaema heterophyllum* Blume; *Arisaema amurense* Maxim. [Araceae; Arisaematis rhizoma / Arisaema cum Bile] |
| Sharen | *Wurfbainia villosa* (Lour.) Škorničk. & A.D.Poulsen; *Wurfbainia villosa* var. *xanthioides* (Wall. ex Baker) Škorničk. & A.D.Poulsen; *Wurfbainia longiligularis* (T.L.Wu) Škorničk. & A.D.Poulsen [Zingiberaceae; Amomi fructus] |
| Binglang | *Areca catechu* L. [Arecaceae; Arecae semen] |
| Shengjiang | *Zingiber officinale* Roscoe [Zingiberaceae; Zingiberis rhizoma recens] |
| Muhudie | *Oroxylum indicum* (L.) Kurz [Bignoniaceae; Oroxyli semen] |
| Banxia / Fabanxia / Qingbanxia | *Pinellia ternata* (Thunb.) Makino [Araceae; Pinelliae rhizoma and its processed preparations] |
| Qingpi | *Citrus reticulata* Blanco [Rutaceae; Citri reticulatae pericarpium viride] |
| Juhong | *Citrus reticulata* Blanco [Rutaceae; Citri exocarpium rubrum] |
| Chenpi | *Citrus reticulata* Blanco [Rutaceae; Citri reticulatae pericarpium] |
| Doukou | *Wurfbainia compacta* (Sol. ex Maton) Škorničk. & A.D.Poulsen [Zingiberaceae; Amomi fructus rotundus] |
| Hehuanpi | *Albizia julibrissin* Durazz. [Fabaceae; Albiziae cortex] |
| Zisugeng | *Perilla frutescens* (L.) Britton [Lamiaceae; Perillae caulis] |
| Zisuye | *Perilla frutescens* (L.) Britton [Lamiaceae; Perillae folium] |
| Zisuzi | *Perilla frutescens* (L.) Britton [Lamiaceae; Perillae fructus] |
| Houpo | *Magnolia officinalis* Rehder & E.H.Wilson [Magnoliaceae; Magnoliae officinalis cortex] |
| Banlangen | *Isatis tinctoria* L. subsp. *tinctoria* [Brassicaceae; Isatidis radix]; *Isatis indigotica* Fortune ex Lindl. is treated as a synonym under current POWO taxonomy |
| Huangqin | *Scutellaria baicalensis* Georgi [Lamiaceae; Scutellariae radix] |
| Zhizi | *Gardenia jasminoides* J.Ellis [Rubiaceae; Gardeniae fructus] |
| Huangbo | *Phellodendron chinense* C.K.Schneid. [Rutaceae; Phellodendri chinensis cortex] |
| Pangdahai | *Scaphium affine* (Mast.) Pierre [Malvaceae; Sterculiae lychnophorae semen] |
| Tuniuxi | *Achyranthes aspera* L. [Amaranthaceae; regional medicinal drug] |
| Malancao | *Aster indicus* L. [Asteraceae; regional medicinal drug]; traditional botanical name *Kalimeris indica* |
| Cheqiancao | *Plantago asiatica* L.; *Plantago depressa* Willd. [Plantaginaceae; Plantaginis herba] |
| Tianmingjing | *Cladocarpesium abrotanoides* (L.) T.Deng & Qun Liu [Asteraceae; regional medicinal drug]; traditional botanical name *Carpesium abrotanoides* L. |
| Fuzi | *Aconitum carmichaelii* Debeaux [Ranunculaceae; Aconiti lateralis radix praeparata] |
| Nanshashen | *Adenophora stricta* Miq.; *Adenophora triphylla* (Thunb.) A.DC. [Campanulaceae; Adenophorae radix] |
| Beishashen | *Glehnia littoralis* F.Schmidt ex Miq. [Apiaceae; Glehniae radix] |
| Shashen | *Adenophora stricta* Miq.; *Adenophora triphylla* (Thunb.) A.DC. [Campanulaceae; Adenophorae radix]; *Glehnia littoralis* F.Schmidt ex Miq. [Apiaceae; Glehniae radix] |
| Baihe | *Lilium lancifolium* Thunb.; *Lilium brownii* Lemoinier; *Lilium pumilum* Redouté [Liliaceae; Lilii bulbus] |
| Tianhuafen | *Trichosanthes kirilowii* Maxim.; *Trichosanthes rosthornii* Harms [Cucurbitaceae; Trichosanthis radix] |
| Shegan | *Iris domestica* (L.) Goldblatt & Mabb. [Iridaceae; Belamcandae rhizoma]; traditional botanical name *Belamcanda chinensis* (L.) Redouté |
| Jiegeng | *Platycodon grandiflorus* (Jacq.) A.DC. [Campanulaceae; Platycodonis radix] |
| Danshen | *Salvia miltiorrhiza* Bunge [Lamiaceae; Salviae miltiorrhizae radix et rhizoma] |
| Chuanxiong | *Ligusticum chuanxiong* S.H.Qiu, Y.Q.Zeng, K.Y.Pan, Y.C.Tang & J.M.Xu [Apiaceae; Chuanxiong rhizoma] |
| Niubangzi | *Arctium lappa* L. [Asteraceae; Arctii fructus] |
| Chaihu | *Bupleurum chinense* DC.; *Bupleurum scorzonerifolium* Willd. [Apiaceae; Bupleuri radix] |
| Huanglian | *Coptis chinensis* Franch.; *Coptis deltoidea* C.Y.Cheng & P.K.Hsiao; *Coptis teeta* Wall. [Ranunculaceae; Coptidis rhizoma] |
| Dahuang | *Rheum palmatum* L.; *Rheum tanguticum* Maxim. ex Balf.; *Rheum officinale* Baill. [Polygonaceae; Rhei radix et rhizoma] |
| Taoren | *Prunus persica* (L.) Batsch; *Prunus davidiana* (Carrière) Franch. [Rosaceae; Persicae semen] |
| Honghua | *Carthamus tinctorius* L. [Asteraceae; Carthami flos] |
| Danggui | *Angelica sinensis* (Oliv.) Diels [Apiaceae; Angelicae sinensis radix] |
| Tiandong | *Asparagus cochinchinensis* (Lour.) Merr. [Asparagaceae; Asparagi radix] |
| Shanyinhua | *Lonicera macranthoides* Hand.-Mazz.; *Lonicera hypoglauca* Miq.; *Lonicera confusa* DC.; *Lonicera fulvotomentosa* P.S.Hsu & S.C.Cheng [Caprifoliaceae; Lonicerae flos] |
| Yuzhu | *Polygonatum odoratum* (Mill.) Druce [Asparagaceae; Polygonati odorati rhizoma] |
| Sangye | *Morus alba* L. [Moraceae; Mori folium] |
| Baibiandou | *Lablab purpureus* (L.) Sweet [Fabaceae; Lablab semen album] |
| Liangmianzhen | *Zanthoxylum nitidum* (Roxb.) DC. [Rutaceae; Zanthoxyli radix] |
| Gualou | *Trichosanthes kirilowii* Maxim.; *Trichosanthes rosthornii* Harms [Cucurbitaceae; Trichosanthis fructus] |
| Gualoupi | *Trichosanthes kirilowii* Maxim.; *Trichosanthes rosthornii* Harms [Cucurbitaceae; Trichosanthis pericarpium] |
| Qiannianjian | *Homalomena occulta* (Lour.) Schott [Araceae; Homalomenae rhizoma] |
| Yuxingcao | *Houttuynia cordata* Thunb. [Saururaceae; Houttuyniae herba] |
| Muxiang | *Dolomiaea costus* (Falc.) Kasana & A.K.Pandey [Asteraceae; Aucklandiae radix]; traditional botanical names include *Aucklandia costus* Falc. and *Saussurea costus* (Falc.) Lipsch. |
| Tinglizi | *Descurainia sophia* (L.) Webb ex Prantl; *Lepidium apetalum* Willd. [Brassicaceae; Descurainiae semen / Lepidii semen] |
| Zhongjiefeng | *Sarcandra glabra* (Thunb.) Nakai [Chloranthaceae; Sarcandrae herba] |
| Sanqi | *Panax notoginseng* (Burkill) F.H.Chen [Araliaceae; Notoginseng radix et rhizoma] |
| Xiaofantianhua | *Urena lobata* L. [Malvaceae; regional medicinal drug] |
| Renshen | *Panax ginseng* C.A.Mey. [Araliaceae; Ginseng radix et rhizoma] |
| Baizhu / Chaobaizhu | *Atractylodes macrocephala* Koidz. [Asteraceae; Atractylodis macrocephalae rhizoma] |
| Shanyao | *Dioscorea polystachya* Turcz. [Dioscoreaceae; Dioscoreae rhizoma]; traditional botanical name *Dioscorea opposita* Thunb. |
| Lianzi | *Nelumbo nucifera* Gaertn. [Nelumbonaceae; Nelumbinis semen] |
| Yiyiren | *Coix lacryma-jobi* var. *ma-yuen* (Rom.Caill.) Stapf [Poaceae; Coicis semen] |
| Huangjing | *Polygonatum kingianum* Collett & Hemsl.; *Polygonatum sibiricum* Redouté; *Polygonatum cyrtonema* Hua [Asparagaceae; Polygonati rhizoma] |
| Cijili | *Tribulus terrestris* L. [Zygophyllaceae; Tribuli fructus] |
| Huangqi | *Astragalus mongholicus* Bunge [Fabaceae; Astragali radix]; pharmacopoeial literature commonly also cites *Astragalus membranaceus* and its var. *mongholicus* |
| Danzhuye | *Lophatherum gracile* Brongn. [Poaceae; Lophatheri herba] |
| Jingmi | *Oryza sativa* L. [Poaceae; medicinal grain component] |
| Wumei | *Prunus mume* (Siebold) Siebold & Zucc. [Rosaceae; Mume fructus] |
| Qingguo | *Canarium album* (Lour.) DC. [Burseraceae; Canarii fructus] |
| Xiqingguo | *Terminalia chebula* Retz. [Combretaceae; Chebulae fructus immaturus] |
| Taizishen | *Pseudostellaria heterophylla* (Miq.) Pax [Caryophyllaceae; Pseudostellariae radix] |
| Jinyinhua | *Lonicera japonica* Thunb. [Caprifoliaceae; Lonicerae japonicae flos] |
| Jingjie | *Nepeta tenuifolia* Benth. [Lamiaceae; Schizonepetae herba]; pharmacopoeial botanical name traditionally cited as *Schizonepeta tenuifolia* (Benth.) Briq. |
| Lianqiao | *Forsythia suspensa* (Thunb.) Vahl [Oleaceae; Forsythiae fructus] |
| Fangfeng | *Saposhnikovia divaricata* (Turcz. ex Ledeb.) Schischk. [Apiaceae; Saposhnikoviae radix] |
| Dazao | *Ziziphus jujuba* Mill. [Rhamnaceae; Jujubae fructus] |
| Dangshen | *Codonopsis pilosula* (Franch.) Nannf.; *Codonopsis pilosula* subsp. *tangshen* (Oliv.) D.Y.Hong and recognized pharmacopoeial source taxa [Campanulaceae; Codonopsis radix] |
| Longdanhua | *Gentiana veitchiorum* Hemsl.; *Gentiana straminea* Maxim.; *Gentiana crassicaulis* Duthie ex Burkill [Gentianaceae; Longdanhua, Tibetan medicinal drug] |
| Liexiangdujuan | *Rhododendron anthopogonoides* Maxim. [Ericaceae; Tibetan medicinal drug] |
| Aizijin | *Corydalis hendersonii* Hemsl. [Papaveraceae; Tibetan medicinal drug] |
| Xiaobopi | *Berberis kansuensis* C.K.Schneid.; *Berberis diaphana* Maxim.; *Berberis vernae* C.K.Schneid.; *Berberis dictyophylla* Franch. [Berberidaceae; Berberidis cortex, Tibetan medicinal source set] |
| Zangmuxiang | *Inula racemosa* Hook.f. [Asteraceae; Tibetan medicinal drug] |
| Pangxiejia | *Phlomoides younghushandii* (Mukerjee) Kamelin & Makhm. [Lamiaceae; Tibetan medicinal drug]; traditional botanical name *Phlomis younghushandii* Mukerjee |
| Jidanshen | *Pseudocodon convolvulaceus* (Kurz) D.Y.Hong & H.Sun [Campanulaceae; Tibetan medicinal drug]; traditional botanical name *Codonopsis convolvulacea* Kurz |
| Maniaopao | *Przewalskia tangutica* Maxim. [Solanaceae; Tibetan medicinal drug] |
| Hezi | *Terminalia chebula* Retz. [Combretaceae; Chebulae fructus] |
| Huzhangcao | *Eriocapitella rivularis* (Buch.-Ham. ex DC.) Christenh. & Byng [Ranunculaceae; regional medicinal drug]; traditional botanical name *Anemone rivularis* Buch.-Ham. ex DC. |
| Wuxiangcao | *Anaphalis bulleyana* (Jeffrey) C.C.Chang [Asteraceae; regional medicinal drug] |
| Zhimu | *Anemarrhena asphodeloides* Bunge [Asparagaceae; Anemarrhenae rhizoma] |
| Shanzhuyu | *Cornus officinalis* Siebold & Zucc. [Cornaceae; Corni fructus] |
| Zexie | *Alisma plantago-aquatica* subsp. *orientale* (Sam.) Sam. [Alismataceae; Alismatis rhizoma]; traditional botanical name *Alisma orientale* (Sam.) Juz. |
