# Supplementary material for: Outcomes reported in randomized controlled trials of traditional Chinese medicine for chronic pharyngitis: a scoping review
Source: Front Pharmacol. 2026 Oct 1;17:1958608. doi: 10.3389/fphar.2026.1958608 (PMC13669160; doi:10.3389/fphar.2026.1958608)
Supplement: Supplementary file 5 [file Table3.docx]

**Appendix A2.2. Taxonomic and pharmacopoeial standardization of medicinal drugs identified in the reviewed TCM preparations--Fungal, algal, animal-derived, mineral, fermented, purified, and other medicinal constituents**

| **Medicinal constituent** | **Standardized description** |
| --- | --- |
| Fuling | Medicinal fungus, *Wolfiporia cocos*; pharmacopoeial drug name: Poria. As fungi are not covered by MPNS/POWO, fungal nomenclature is treated separately from vascular-plant taxonomy. |
| Liushenqu | Fermented multicomponent medicinal preparation; not assignable to a single botanical species. |
| Chantui | Animal-derived cicada exuviae; not a botanical drug. |
| Guijia / Guiban | Animal-derived turtle plastron or shell medicinal material; not botanical. |
| Tianzhuhuang | Processed bamboo-associated siliceous medicinal material; the botanical source is preparation-standard dependent and the drug itself is not treated as an intact vascular botanical drug. |
| Jiangcan | Processed silkworm-derived medicinal material associated with fungal infection; not a vascular botanical drug. |
| Artificial Calculus Bovis | Artificial or processed animal-derived medicinal material; not botanical. |
| Porcine bile powder | Animal-derived medicinal material; not botanical. |
| Nacre powder | Animal-derived biogenic mineral material; not botanical. |
| Concentrated buffalo horn powder | Animal-derived medicinal material; not botanical. |
| Hongqu | Fermented fungal product; not a vascular botanical drug. |
| Indomethacin | Synthetic pharmaceutical compound. |
| Andrographolide | Purified diterpenoid compound; not equivalent to administration of the crude botanical drug *Andrographis paniculata*. |
| Haizao | Brown algal medicinal material, generally derived from pharmacopoeially specified *Sargassum* species; algae are outside the taxonomic scope of MPNS/POWO. |
| Haigefen | Shell-derived or mineralized medicinal material; not botanical. |
| Shigao | Mineral drug, gypsum (calcium sulfate dihydrate); not botanical. |
| Bingpian | Borneol-containing processed medicinal material; botanical origin should not be inferred when the preparation does not specify natural versus synthetic source. |
| Peppermint oil | Processed essential oil corresponding to a *Mentha* source under applicable product standards; treated as a processed botanical preparation rather than an intact herbal drug. |
| Menthol | Purified compound that may be natural or synthetic; no botanical source is assigned solely from the compound name. |
| Xiguashuang | Processed composite medicinal material; not treated as a single botanical species. |
| Fenghuangyi | Chicken eggshell membrane; animal-derived medicinal material. |
