# Supplementary material for: Outcomes reported in randomized controlled trials of traditional Chinese medicine for chronic pharyngitis: a scoping review
Source: Front Pharmacol. 2026 Oct 1;17:1958608. doi: 10.3389/fphar.2026.1958608 (PMC13669160; doi:10.3389/fphar.2026.1958608)
Supplement: Supplementary file 6 [file Table4.docx]

\### traditional Chinese medicine

#1 "traditional Chinese medicine" [MeSH]

#2 "Chinese patent medicine" [MeSH]

#3 "traditional Chinese medicine injection" [MeSH]

#4 ("traditional Chinese medicine*" OR "Chinese patent medicine*" OR "traditional Chinese medicine injection*" OR "TCM") [TIAB]

#5 ("particle*" OR "granule*" OR "Keli" OR "capsule*" OR "Jiaonang" OR "oral liquid*" OR "oral liquids*" OR "oral solution*" OR "san" OR "syrup*" OR "sirop*" OR "Pill*" OR "injection*" OR "injectable*" OR "injections*" OR "injectables*" OR "Pian*" OR "wan" OR "heji" OR "mixture" OR "dropping pill" OR "drop pill" OR "tangjiang" OR "dan" OR "aerosol" OR "spray" OR "qiwuji" OR "decoction" OR "Yin") [TIAB]

#6= "#1" OR "#2" OR "#3" OR "#4" OR "#5"

\### Study types

#7 "Randomized Controlled Trial" [Publication Type]

#8 "Randomized Controlled Trials as Topic" [MeSH]

#9 "Clinical Trial" [Publication Type]

#10 ("randomized controlled study" OR "randomized controlled trial" OR "randomized study" OR "randomized trial" OR "randomized placebo-controlled study" OR "randomized placebo-controlled trial" OR "randomized placebo controlled" OR "randomized placebo-controlled" OR "randomized double-blin*" OR "randomized double blin*") [TIAB]

#11= "#7" OR "#8" OR "#9" OR "#10"

\### chronic pharyngitis

#12 "chronic pharyngitis" [MeSH]

#13 ("chronic pharyngitis" OR "Sore Throat*" )[TIAB]

#14= "#12" OR "#13"

#15= "#6" AND "#11" AND "#14"
