# Supplementary material for: Outcomes reported in randomized controlled trials of traditional Chinese medicine for chronic pharyngitis: a scoping review
Source: Front Pharmacol. 2026 Oct 1;17:1958608. doi: 10.3389/fphar.2026.1958608 (PMC13669160; doi:10.3389/fphar.2026.1958608)
Supplement: Supplementary file 7 [file Table5.docx]

**Supplementary Table X2. Cross-mapping of standardized outcome terms between the COMET 12-category framework and the seven-domain TCM outcome classification**

| **Outcome indicator** | **Frequency (n)** | **Seven-domain TCM classification** | **COMET 12-category** | **Mapping rationale / note** |
| --- | --- | --- | --- | --- |
| Abnormal pulse manifestation | 2 | TCM Syndrome | Physiological/pathophysiological | TCM syndrome/disease-state construct; no separate TCM category exists in the 12-category COMET framework, so it maps to Physiological/pathophysiological. |
| Abnormal tongue manifestation | 3 | TCM Syndrome | Physiological/pathophysiological | TCM syndrome/disease-state construct; no separate TCM category exists in the 12-category COMET framework, so it maps to Physiological/pathophysiological. |
| Efficacy on individual TCM syndrome items | 6 | TCM Syndrome | Physiological/pathophysiological | TCM syndrome/disease-state construct; no separate TCM category exists in the 12-category COMET framework, so it maps to Physiological/pathophysiological. |
| Efficacy on TCM syndrome | 12 | TCM Syndrome | Physiological/pathophysiological | TCM syndrome/disease-state construct; no separate TCM category exists in the 12-category COMET framework, so it maps to Physiological/pathophysiological. |
| Individual TCM symptom score | 2 | TCM Syndrome | Physiological/pathophysiological | TCM syndrome/disease-state construct; no separate TCM category exists in the 12-category COMET framework, so it maps to Physiological/pathophysiological. |
| Individual TCM syndrome item score | 5 | TCM Syndrome | Physiological/pathophysiological | TCM syndrome/disease-state construct; no separate TCM category exists in the 12-category COMET framework, so it maps to Physiological/pathophysiological. |
| TCM symptom score | 2 | TCM Syndrome | Physiological/pathophysiological | TCM syndrome/disease-state construct; no separate TCM category exists in the 12-category COMET framework, so it maps to Physiological/pathophysiological. |
| TCM syndrome score | 3 | TCM Syndrome | Physiological/pathophysiological | TCM syndrome/disease-state construct; no separate TCM category exists in the 12-category COMET framework, so it maps to Physiological/pathophysiological. |
| Abnormal taste | 1 | Symptoms/Signs | Physiological/pathophysiological | Clinical symptom/sign or disease manifestation; mapped to Physiological/pathophysiological unless a more specific COMET category (e.g., Pain or Function) applies. |
| Aggravation after excessive talking | 3 | Symptoms/Signs | Physiological/pathophysiological | Clinical symptom/sign or disease manifestation; mapped to Physiological/pathophysiological unless a more specific COMET category (e.g., Pain or Function) applies. |
| Burning sensation | 21 | Symptoms/Signs | Physiological/pathophysiological | Clinical symptom/sign or disease manifestation; mapped to Physiological/pathophysiological unless a more specific COMET category (e.g., Pain or Function) applies. |
| Burning sensation / Foreign body sensation | 2 | Symptoms/Signs | Physiological/pathophysiological | Clinical symptom/sign or disease manifestation; mapped to Physiological/pathophysiological unless a more specific COMET category (e.g., Pain or Function) applies. |
| Changes in the pharyngeal mucosa | 2 | Symptoms/Signs | Physiological/pathophysiological | Clinical symptom/sign or disease manifestation; mapped to Physiological/pathophysiological unless a more specific COMET category (e.g., Pain or Function) applies. |
| Clinical efficacy | 299 | Symptoms/Signs | Physiological/pathophysiological | Global treatment-response/disease-status outcome; assigned to Physiological/pathophysiological. |
| Cough with excessive sputum | 2 | Symptoms/Signs | Physiological/pathophysiological | Clinical symptom/sign or disease manifestation; mapped to Physiological/pathophysiological unless a more specific COMET category (e.g., Pain or Function) applies. |
| Cure rate | 1 | Symptoms/Signs | Physiological/pathophysiological | Global treatment-response/disease-status outcome; assigned to Physiological/pathophysiological. |
| Dry and itchy throat | 17 | Symptoms/Signs | Physiological/pathophysiological | Clinical symptom/sign or disease manifestation; mapped to Physiological/pathophysiological unless a more specific COMET category (e.g., Pain or Function) applies. |
| Dry and sore throat | 3 | Symptoms/Signs | Pain | Pain-related symptom or pain subscale; assigned to the COMET Pain category. |
| Dry cough | 45 | Symptoms/Signs | Physiological/pathophysiological | Clinical symptom/sign or disease manifestation; mapped to Physiological/pathophysiological unless a more specific COMET category (e.g., Pain or Function) applies. |
| Dry cough with sputum | 1 | Symptoms/Signs | Physiological/pathophysiological | Clinical symptom/sign or disease manifestation; mapped to Physiological/pathophysiological unless a more specific COMET category (e.g., Pain or Function) applies. |
| Dry mouth | 1 | Symptoms/Signs | Physiological/pathophysiological | Clinical symptom/sign or disease manifestation; mapped to Physiological/pathophysiological unless a more specific COMET category (e.g., Pain or Function) applies. |
| Dry stool | 1 | Symptoms/Signs | Physiological/pathophysiological | Clinical symptom/sign or disease manifestation; mapped to Physiological/pathophysiological unless a more specific COMET category (e.g., Pain or Function) applies. |
| Dry throat | 50 | Symptoms/Signs | Physiological/pathophysiological | Clinical symptom/sign or disease manifestation; mapped to Physiological/pathophysiological unless a more specific COMET category (e.g., Pain or Function) applies. |
| Dry throat with burning sensation | 1 | Symptoms/Signs | Physiological/pathophysiological | Clinical symptom/sign or disease manifestation; mapped to Physiological/pathophysiological unless a more specific COMET category (e.g., Pain or Function) applies. |
| Dry throat with reduced fluid/moisture | 1 | Symptoms/Signs | Physiological/pathophysiological | Clinical symptom/sign or disease manifestation; mapped to Physiological/pathophysiological unless a more specific COMET category (e.g., Pain or Function) applies. |
| Dysphagia | 1 | Symptoms/Signs | Function/functional status | Represents impairment of swallowing function; assigned to Function/functional status. |
| Expectoration | 8 | Symptoms/Signs | Physiological/pathophysiological | Clinical symptom/sign or disease manifestation; mapped to Physiological/pathophysiological unless a more specific COMET category (e.g., Pain or Function) applies. |
| Foreign body sensation | 64 | Symptoms/Signs | Physiological/pathophysiological | Clinical symptom/sign or disease manifestation; mapped to Physiological/pathophysiological unless a more specific COMET category (e.g., Pain or Function) applies. |
| Halitosis | 1 | Symptoms/Signs | Physiological/pathophysiological | Clinical symptom/sign or disease manifestation; mapped to Physiological/pathophysiological unless a more specific COMET category (e.g., Pain or Function) applies. |
| Hoarseness | 9 | Symptoms/Signs | Physiological/pathophysiological | Clinical symptom/sign or disease manifestation; mapped to Physiological/pathophysiological unless a more specific COMET category (e.g., Pain or Function) applies. |
| Hyperemia and hypertrophy of the lateral pharyngeal bands | 1 | Symptoms/Signs | Physiological/pathophysiological | Clinical symptom/sign or disease manifestation; mapped to Physiological/pathophysiological unless a more specific COMET category (e.g., Pain or Function) applies. |
| Hyperemia of the posterior pharyngeal wall | 4 | Symptoms/Signs | Physiological/pathophysiological | Clinical symptom/sign or disease manifestation; mapped to Physiological/pathophysiological unless a more specific COMET category (e.g., Pain or Function) applies. |
| Hypertrophy of the lateral pharyngeal bands | 4 | Symptoms/Signs | Physiological/pathophysiological | Clinical symptom/sign or disease manifestation; mapped to Physiological/pathophysiological unless a more specific COMET category (e.g., Pain or Function) applies. |
| Itchy throat | 45 | Symptoms/Signs | Physiological/pathophysiological | Clinical symptom/sign or disease manifestation; mapped to Physiological/pathophysiological unless a more specific COMET category (e.g., Pain or Function) applies. |
| Itchy throat with dry cough | 1 | Symptoms/Signs | Physiological/pathophysiological | Clinical symptom/sign or disease manifestation; mapped to Physiological/pathophysiological unless a more specific COMET category (e.g., Pain or Function) applies. |
| Lymphoid follicular hyperplasia at the base of the tongue | 1 | Symptoms/Signs | Physiological/pathophysiological | Clinical symptom/sign or disease manifestation; mapped to Physiological/pathophysiological unless a more specific COMET category (e.g., Pain or Function) applies. |
| Main symptoms | 3 | Symptoms/Signs | Physiological/pathophysiological | Clinical symptom/sign or disease manifestation; mapped to Physiological/pathophysiological unless a more specific COMET category (e.g., Pain or Function) applies. |
| Morning retching | 4 | Symptoms/Signs | Physiological/pathophysiological | Clinical symptom/sign or disease manifestation; mapped to Physiological/pathophysiological unless a more specific COMET category (e.g., Pain or Function) applies. |
| Mucosal dryness | 6 | Symptoms/Signs | Physiological/pathophysiological | Clinical symptom/sign or disease manifestation; mapped to Physiological/pathophysiological unless a more specific COMET category (e.g., Pain or Function) applies. |
| Mucosal edema | 5 | Symptoms/Signs | Physiological/pathophysiological | Clinical symptom/sign or disease manifestation; mapped to Physiological/pathophysiological unless a more specific COMET category (e.g., Pain or Function) applies. |
| Mucosal hyperemia | 32 | Symptoms/Signs | Physiological/pathophysiological | Clinical symptom/sign or disease manifestation; mapped to Physiological/pathophysiological unless a more specific COMET category (e.g., Pain or Function) applies. |
| Mucosal hyperemia and edema | 2 | Symptoms/Signs | Physiological/pathophysiological | Clinical symptom/sign or disease manifestation; mapped to Physiological/pathophysiological unless a more specific COMET category (e.g., Pain or Function) applies. |
| Pharyngalgia | 71 | Symptoms/Signs | Pain | Pain-related symptom or pain subscale; assigned to the COMET Pain category. |
| Pharyngeal mucosal hyperemia and edema with lymphoid follicular hyperplasia | 1 | Symptoms/Signs | Physiological/pathophysiological | Clinical symptom/sign or disease manifestation; mapped to Physiological/pathophysiological unless a more specific COMET category (e.g., Pain or Function) applies. |
| Pharyngeal redness and swelling | 2 | Symptoms/Signs | Physiological/pathophysiological | Clinical symptom/sign or disease manifestation; mapped to Physiological/pathophysiological unless a more specific COMET category (e.g., Pain or Function) applies. |
| Pharyngeal signs | 14 | Symptoms/Signs | Physiological/pathophysiological | Clinical symptom/sign or disease manifestation; mapped to Physiological/pathophysiological unless a more specific COMET category (e.g., Pain or Function) applies. |
| Pharyngeal symptoms | 22 | Symptoms/Signs | Physiological/pathophysiological | Clinical symptom/sign or disease manifestation; mapped to Physiological/pathophysiological unless a more specific COMET category (e.g., Pain or Function) applies. |
| Posterior pharyngeal wall lymphoid follicular hyperplasia | 24 | Symptoms/Signs | Physiological/pathophysiological | Clinical symptom/sign or disease manifestation; mapped to Physiological/pathophysiological unless a more specific COMET category (e.g., Pain or Function) applies. |
| Pseudomembrane shedding | 1 | Symptoms/Signs | Physiological/pathophysiological | Clinical symptom/sign or disease manifestation; mapped to Physiological/pathophysiological unless a more specific COMET category (e.g., Pain or Function) applies. |
| Redness and swelling of the lateral pharyngeal bands | 2 | Symptoms/Signs | Physiological/pathophysiological | Clinical symptom/sign or disease manifestation; mapped to Physiological/pathophysiological unless a more specific COMET category (e.g., Pain or Function) applies. |
| Secondary symptoms | 3 | Symptoms/Signs | Physiological/pathophysiological | Clinical symptom/sign or disease manifestation; mapped to Physiological/pathophysiological unless a more specific COMET category (e.g., Pain or Function) applies. |
| Secretions | 13 | Symptoms/Signs | Physiological/pathophysiological | Clinical symptom/sign or disease manifestation; mapped to Physiological/pathophysiological unless a more specific COMET category (e.g., Pain or Function) applies. |
| Sensation of sticky sputum | 9 | Symptoms/Signs | Physiological/pathophysiological | Clinical symptom/sign or disease manifestation; mapped to Physiological/pathophysiological unless a more specific COMET category (e.g., Pain or Function) applies. |
| Sore throat aggravated by excessive talking | 2 | Symptoms/Signs | Pain | Pain-related symptom or pain subscale; assigned to the COMET Pain category. |
| Sore throat with bleeding | 2 | Symptoms/Signs | Pain | Pain-related symptom or pain subscale; assigned to the COMET Pain category. |
| Symptom and sign score | 12 | Symptoms/Signs | Physiological/pathophysiological | Clinical symptom/sign or disease manifestation; mapped to Physiological/pathophysiological unless a more specific COMET category (e.g., Pain or Function) applies. |
| Symptoms and signs | 3 | Symptoms/Signs | Physiological/pathophysiological | Clinical symptom/sign or disease manifestation; mapped to Physiological/pathophysiological unless a more specific COMET category (e.g., Pain or Function) applies. |
| Thickening of the pharyngeal mucosa | 5 | Symptoms/Signs | Physiological/pathophysiological | Clinical symptom/sign or disease manifestation; mapped to Physiological/pathophysiological unless a more specific COMET category (e.g., Pain or Function) applies. |
| Throat clearing | 1 | Symptoms/Signs | Physiological/pathophysiological | Clinical symptom/sign or disease manifestation; mapped to Physiological/pathophysiological unless a more specific COMET category (e.g., Pain or Function) applies. |
| Time to cure | 1 | Symptoms/Signs | Physiological/pathophysiological | Global treatment-response/disease-status outcome; assigned to Physiological/pathophysiological. |
| Tonsillar hypertrophy | 1 | Symptoms/Signs | Physiological/pathophysiological | Clinical symptom/sign or disease manifestation; mapped to Physiological/pathophysiological unless a more specific COMET category (e.g., Pain or Function) applies. |
| Vasodilation of the posterior pharyngeal wall | 3 | Symptoms/Signs | Physiological/pathophysiological | Clinical symptom/sign or disease manifestation; mapped to Physiological/pathophysiological unless a more specific COMET category (e.g., Pain or Function) applies. |
| Yellow urine | 1 | Symptoms/Signs | Physiological/pathophysiological | Clinical symptom/sign or disease manifestation; mapped to Physiological/pathophysiological unless a more specific COMET category (e.g., Pain or Function) applies. |
| ALT | 1 | Physical and chemical examination | Physiological/pathophysiological | Objective laboratory, biomarker, physiological, or pathological indicator. |
| AST | 1 | Physical and chemical examination | Physiological/pathophysiological | Objective laboratory, biomarker, physiological, or pathological indicator. |
| B cells | 1 | Physical and chemical examination | Physiological/pathophysiological | Objective laboratory, biomarker, physiological, or pathological indicator. |
| BUN | 1 | Physical and chemical examination | Physiological/pathophysiological | Objective laboratory, biomarker, physiological, or pathological indicator. |
| CD3+ | 10 | Physical and chemical examination | Physiological/pathophysiological | Objective laboratory, biomarker, physiological, or pathological indicator. |
| CD4+ | 12 | Physical and chemical examination | Physiological/pathophysiological | Objective laboratory, biomarker, physiological, or pathological indicator. |
| CD4+/CD8+ | 11 | Physical and chemical examination | Physiological/pathophysiological | Objective laboratory, biomarker, physiological, or pathological indicator. |
| CD8+ | 11 | Physical and chemical examination | Physiological/pathophysiological | Objective laboratory, biomarker, physiological, or pathological indicator. |
| CRP | 12 | Physical and chemical examination | Physiological/pathophysiological | Objective laboratory, biomarker, physiological, or pathological indicator. |
| Dysbiosis | 1 | Physical and chemical examination | Physiological/pathophysiological | Objective laboratory, biomarker, physiological, or pathological indicator. |
| EGF | 2 | Physical and chemical examination | Physiological/pathophysiological | Objective laboratory, biomarker, physiological, or pathological indicator. |
| EOS | 3 | Physical and chemical examination | Physiological/pathophysiological | Objective laboratory, biomarker, physiological, or pathological indicator. |
| hepatic and renal function | 1 | Physical and chemical examination | Physiological/pathophysiological | Objective laboratory, biomarker, physiological, or pathological indicator. |
| hs-CRP | 6 | Physical and chemical examination | Physiological/pathophysiological | Objective laboratory, biomarker, physiological, or pathological indicator. |
| IgA | 3 | Physical and chemical examination | Physiological/pathophysiological | Objective laboratory, biomarker, physiological, or pathological indicator. |
| IgE | 1 | Physical and chemical examination | Physiological/pathophysiological | Objective laboratory, biomarker, physiological, or pathological indicator. |
| IgG | 1 | Physical and chemical examination | Physiological/pathophysiological | Objective laboratory, biomarker, physiological, or pathological indicator. |
| IgM | 1 | Physical and chemical examination | Physiological/pathophysiological | Objective laboratory, biomarker, physiological, or pathological indicator. |
| IL-1 | 2 | Physical and chemical examination | Physiological/pathophysiological | Objective laboratory, biomarker, physiological, or pathological indicator. |
| IL-1β | 5 | Physical and chemical examination | Physiological/pathophysiological | Objective laboratory, biomarker, physiological, or pathological indicator. |
| IL-2 | 12 | Physical and chemical examination | Physiological/pathophysiological | Objective laboratory, biomarker, physiological, or pathological indicator. |
| IL-6 | 29 | Physical and chemical examination | Physiological/pathophysiological | Objective laboratory, biomarker, physiological, or pathological indicator. |
| IL-8 | 1 | Physical and chemical examination | Physiological/pathophysiological | Objective laboratory, biomarker, physiological, or pathological indicator. |
| LY | 2 | Physical and chemical examination | Physiological/pathophysiological | Objective laboratory, biomarker, physiological, or pathological indicator. |
| MMP-9 | 4 | Physical and chemical examination | Physiological/pathophysiological | Objective laboratory, biomarker, physiological, or pathological indicator. |
| MO | 1 | Physical and chemical examination | Physiological/pathophysiological | Objective laboratory, biomarker, physiological, or pathological indicator. |
| NEUT | 4 | Physical and chemical examination | Physiological/pathophysiological | Objective laboratory, biomarker, physiological, or pathological indicator. |
| PCT | 1 | Physical and chemical examination | Physiological/pathophysiological | Objective laboratory, biomarker, physiological, or pathological indicator. |
| PGE2 | 1 | Physical and chemical examination | Physiological/pathophysiological | Objective laboratory, biomarker, physiological, or pathological indicator. |
| Positive expression rate of PCNA in pharyngeal lesion tissues | 1 | Physical and chemical examination | Physiological/pathophysiological | Objective laboratory, biomarker, physiological, or pathological indicator. |
| routine blood and urine examination | 1 | Physical and chemical examination | Physiological/pathophysiological | Objective laboratory, biomarker, physiological, or pathological indicator. |
| Scr | 1 | Physical and chemical examination | Physiological/pathophysiological | Objective laboratory, biomarker, physiological, or pathological indicator. |
| SIgA | 4 | Physical and chemical examination | Physiological/pathophysiological | Objective laboratory, biomarker, physiological, or pathological indicator. |
| TNF-α | 38 | Physical and chemical examination | Physiological/pathophysiological | Objective laboratory, biomarker, physiological, or pathological indicator. |
| VCAM-1 | 4 | Physical and chemical examination | Physiological/pathophysiological | Objective laboratory, biomarker, physiological, or pathological indicator. |
| WBC | 1 | Physical and chemical examination | Physiological/pathophysiological | Objective laboratory, biomarker, physiological, or pathological indicator. |
| Bodily Pain(SF-36-BP) | 2 | quality of life | Pain | Pain-related symptom or pain subscale; assigned to the COMET Pain category. |
| General Health(SF-36-GH) | 2 | quality of life | Quality of life | Overall/general quality-of-life or broad health-status construct. |
| Material Life Status(GQOL-74-MLS) | 1 | quality of life | Quality of life | Overall/general quality-of-life or broad health-status construct. |
| Medication Adherence | 1 | quality of life | Compliance with treatment | Direct measure of adherence/compliance with the intervention. |
| Mental Health(SF-36-MH) | 2 | quality of life | Mental health | Psychological or emotional health construct; assigned according to the underlying construct rather than the parent quality-of-life instrument. |
| Patient Satisfaction | 2 | quality of life | Other | Patient satisfaction is distinct from quality of life; the 12-category COMET framework used here has no separate satisfaction category, so it is placed under Other. |
| Physical Function(GQOL-74-PF) | 1 | quality of life | Function/functional status | Functional-status subscale/construct; assigned according to the specific construct rather than the parent quality-of-life instrument. |
| Physical Functioning(SF-36-PF) | 2 | quality of life | Function/functional status | Functional-status subscale/construct; assigned according to the specific construct rather than the parent quality-of-life instrument. |
| Psychological Function(GQOL-74-PsF) | 1 | quality of life | Mental health | Psychological or emotional health construct; assigned according to the underlying construct rather than the parent quality-of-life instrument. |
| Role-Emotional(SF-36-RE) | 2 | quality of life | Psychosocial | Social or role-emotional functioning construct; assigned to Psychosocial. |
| Role-Physical(SF-36-RP) | 2 | quality of life | Function/functional status | Functional-status subscale/construct; assigned according to the specific construct rather than the parent quality-of-life instrument. |
| Satisfaction Rate(SR) | 1 | quality of life | Other | Patient satisfaction is distinct from quality of life; the 12-category COMET framework used here has no separate satisfaction category, so it is placed under Other. |
| Self-Rating Anxiety Scale(SAS) | 2 | quality of life | Mental health | Psychological or emotional health construct; assigned according to the underlying construct rather than the parent quality-of-life instrument. |
| Self-Rating Depression Scale(SDS) | 2 | quality of life | Mental health | Psychological or emotional health construct; assigned according to the underlying construct rather than the parent quality-of-life instrument. |
| SF-36 score | 1 | quality of life | Quality of life | Overall/general quality-of-life or broad health-status construct. |
| Social Function(GQOL-74-SF) | 1 | quality of life | Psychosocial | Social or role-emotional functioning construct; assigned to Psychosocial. |
| Social Functioning(SF-36-SF) | 2 | quality of life | Psychosocial | Social or role-emotional functioning construct; assigned to Psychosocial. |
| Vitality(SF-36-VT) | 2 | quality of life | Quality of life | Overall/general quality-of-life or broad health-status construct. |
| Prognosis | 1 | long-term prognosis | Physiological/pathophysiological | Disease course/prognostic outcome (e.g., recurrence or prognosis); mapped to Physiological/pathophysiological in the 12-category framework. |
| Recurrence rate | 32 | long-term prognosis | Physiological/pathophysiological | Disease course/prognostic outcome (e.g., recurrence or prognosis); mapped to Physiological/pathophysiological in the 12-category framework. |
| Cost-effectiveness ratio(CER) | 2 | Economic evaluation | Resource use | Economic/resource-use outcome, including cost or cost-effectiveness. |
| Drug cost(C) | 1 | Economic evaluation | Resource use | Economic/resource-use outcome, including cost or cost-effectiveness. |
| Effectiveness(E) | 1 | Economic evaluation | Physiological/pathophysiological | Represents the clinical-effect component used in an economic analysis; classified by its clinical construct rather than as resource use. |
| Incremental cost-effectiveness ratio(ICER) | 1 | Economic evaluation | Resource use | Economic/resource-use outcome, including cost or cost-effectiveness. |
| Incidence of adverse reactions | 69 | Security event | Adverse events | Treatment-related harm, adverse reaction, side effect, or safety outcome. |
| Incidence of complications | 2 | Security event | Adverse events | Retained as Adverse events because the source dataset classified this item as a safety event; verify against the original trial if a complication was disease-related rather than treatment-related. |
| Incidence of side effects | 3 | Security event | Adverse events | Treatment-related harm, adverse reaction, side effect, or safety outcome. |
| Safety assessment | 1 | Security event | Adverse events | Treatment-related harm, adverse reaction, side effect, or safety outcome. |
| Toxic side effects | 1 | Security event | Adverse events | Treatment-related harm, adverse reaction, side effect, or safety outcome. |
