# Supplementary material for: Outcomes reported in randomized controlled trials of traditional Chinese medicine for chronic pharyngitis: a scoping review
Source: Front Pharmacol. 2026 Oct 1;17:1958608. doi: 10.3389/fphar.2026.1958608 (PMC13669160; doi:10.3389/fphar.2026.1958608)
Supplement: Supplementary file 8 [file Table6.docx]

**Supplementary Table X3. Operational definitions and principal criteria used to determine clinical efficacy in the included trials**

| **Operational definition category** | **Reference standard/source** | **Principal criteria used to determine clinical efficacy** | **Typical grading/thresholds** | **Key characteristics** |
| --- | --- | --- | --- | --- |
| **Symptom response with recurrence-based criteria** | *Criteria of Diagnosis and Therapeutic Effect of Diseases and Syndromes in Traditional Chinese Medicine* | Treatment response was determined primarily according to changes in throat-related symptoms. Recurrence during follow-up was additionally incorporated into the efficacy classification. | Markedly effective: symptoms disappeared and no recurrence occurred within 3 months; effective: symptoms improved or recurrence occurred within 3 months; ineffective: symptoms did not improve. | Distinct from most other definitions because recurrence was incorporated directly into the definition of short-term clinical efficacy. |
| **Symptom/sign score-reduction–based efficacy** | *Guidelines for Clinical Research of New Chinese Medicines* | Symptoms and pharyngeal signs were scored before and after treatment. An efficacy index was calculated from the proportional reduction in the total symptom/sign score. | Cured: ≥95% reduction; markedly effective: ≥70%; effective: ≥30%; ineffective: <30%. | Quantitative definition based on percentage reduction in symptom/sign scores. |
| **Symptom/sign improvement–based categorical efficacy** | *Guidelines for Clinical Research of New Chinese Medicines for Chronic Pharyngitis* | Efficacy was classified according to the disappearance or degree of improvement of clinical symptoms and pharyngeal signs. | Typically classified as cured, markedly effective, effective, or ineffective. | Relied on categorical clinical improvement rather than a uniform numerical reduction threshold. |
| **Symptom/sign improvement–based categorical efficacy** | *Practical Otolaryngology* | Changes in subjective symptoms and objective pharyngeal findings, including mucosal congestion, lymphoid follicular hyperplasia, and secretions, were considered jointly. | Response categories were based on disappearance, improvement, or persistence of symptoms and signs. | Combined patient-reported symptoms with observable pharyngeal signs. |
| **Symptom/sign improvement–based categorical efficacy** | *Otolaryngology–Head and Neck Surgery* | Clinical response was determined according to changes in symptoms such as throat pain and itching together with changes in mucosal redness/swelling and inflammatory secretions. | Markedly effective, effective, and ineffective categories were used according to the degree of improvement. | A three-level response scheme was used in some studies rather than the four-level scheme used elsewhere. |
| **Composite clinical and objective efficacy** | *Diagnostics of Otolaryngology* | Clinical symptoms were assessed together with objective laboratory indicators. Improvement or normalization of both components contributed to the efficacy classification. | Higher response categories required disappearance or substantial improvement of clinical symptoms together with normalization or near-normalization of relevant serum indicators. | Clinical efficacy incorporated laboratory measurements in addition to symptoms and signs. |
| **Symptom/sign improvement–based categorical efficacy** | *Diagnostic and Therapeutic Effect Criteria for Diseases and Syndromes in Traditional Chinese Medicine Otolaryngology* (1994 industry standard) | Clinical efficacy was determined according to improvement in throat discomfort and objective pharyngeal findings such as congestion and secretions. | Typically classified as cured, markedly effective, effective, or ineffective according to the degree of symptom/sign resolution. | Primarily symptom- and examination-based. |
| **Symptom/sign improvement–based categorical efficacy** | *Criteria for Diagnosis, Cure, and Improvement of Clinical Diseases* | Efficacy was determined from changes in pharyngeal discomfort and physical examination findings, including mucosal congestion and secretions. | Cured, markedly effective, effective, and ineffective categories were defined according to the extent of clinical improvement. | Qualitative categorical assessment without a uniform percentage threshold. |
| **Symptom/sign improvement–based categorical efficacy** | *Traditional Chinese Medicine Otolaryngology and Stomatology* | Clinical efficacy was assessed using changes in throat symptoms and objective pharyngeal findings, including congestion and lymphoid follicular changes. | Cure required resolution of symptoms and relevant pharyngeal abnormalities; improvement represented partial resolution; ineffective indicated no meaningful change. | Closely linked to conventional clinical manifestations and pharyngeal examination. |
| **Symptom/sign score-reduction–based efficacy** | *Diagnostic and Therapeutic Standards for Traditional Chinese Medicine Diseases and Syndromes and Formula Selection* | Clinical symptoms and laryngoscopic findings were evaluated before and after treatment, and efficacy was determined according to the proportional change in the total score. | Cured: improvement rate ≥90%; markedly effective: 66%–90%; effective: 33%–66%; ineffective: <33%. | Demonstrates that numerical thresholds were not uniform across different standards. |
| **Symptom/sign improvement–based categorical efficacy** | *Clinician's Handbook: Otolaryngology* | Treatment response was classified according to the disappearance or improvement of symptoms and objective pharyngeal signs. | Typically classified as cured, markedly effective, effective, or ineffective. | Qualitative clinical-response classification. |
| **Symptom/sign improvement–based categorical efficacy** | *Encyclopedia of Otolaryngology: Pharyngology* | Subjective symptoms and objective findings such as pharyngeal mucosal congestion and lymphoid follicular hyperplasia were jointly considered. | Higher efficacy categories required disappearance or marked improvement of both symptoms and physical signs. | Combined subjective and examiner-observed clinical manifestations. |
| **Symptom/sign score-reduction–based efficacy** | *Traditional Chinese Medicine Diagnosis and Treatment Protocols for 95 Diseases in 22 Specialties* | Total symptom/sign scores before and after treatment were compared, and the percentage reduction was used to determine clinical response. | Cured: ≥95% reduction; markedly effective: 70%–<95%; effective: 30%–<70%; ineffective: <30%. | Quantitative score-based classification with predefined thresholds. |
| **Symptom/sign improvement–based categorical efficacy** | *Routine Diagnosis and Treatment of Common Traditional Chinese Medicine Diseases and Syndromes* | Efficacy was determined according to clinical manifestations and objective examination findings after treatment. | Typically classified into markedly effective, effective, and ineffective according to the degree of symptom/sign improvement. | Represents a categorical clinical assessment based on both subjective and objective findings. |
| **Symptom/sign improvement–based categorical efficacy** | Criteria proposed at the 1992 National Pharyngology Conference for chronic pharyngitis | Treatment response was based on resolution or improvement of major symptoms together with objective changes in pharyngeal redness/congestion and lymphoid follicular hyperplasia. | Complete response required disappearance of major symptoms and marked resolution of objective pharyngeal abnormalities. | Conference-based criteria emphasizing both clinical symptoms and pharyngeal examination. |
| **Reference source not clearly reported** | Not reported in the primary study | The primary report did not identify the reference standard used for determining clinical efficacy. | Not applicable. | **52 of 299 clinical-efficacy occurrences (17.4%)** did not clearly identify the source of the efficacy criteria. This category refers to missing information on the source of the criteria and should not automatically be interpreted as absence of an operational definition if descriptive criteria were otherwise provided in the article. |
