# Supplementary material for: Outcomes reported in randomized controlled trials of traditional Chinese medicine for chronic pharyngitis: a scoping review
Source: Front Pharmacol. 2026 Oct 1;17:1958608. doi: 10.3389/fphar.2026.1958608 (PMC13669160; doi:10.3389/fphar.2026.1958608)
Supplement: Supplementary file 9 [file Table7.docx]

**Supplementary Table X4. Cross-analysis of treatment duration and outcome assessment timing in the included trials**

| **Treatment duration (days)** | **No. of trials** | **Trials with ≥1 clearly specified assessment time point, n (%)ᵃ** | **Assessment at treatment completion, n (%)ᵇ** | **Assessment before treatment completion, n (%)ᵇᶜ** | **Post-treatment assessment, n (%)ᵇᶜ** | **Only treatment-completion time point clearly specified, n (%)ᵇᵈ** |
| --- | --- | --- | --- | --- | --- | --- |
| 5 | 10 | 10 (100.0%) | 10 (100.0%) | 0 (0.0%) | 0 (0.0%) | 10 (100.0%) |
| 6 | 4 | 4 (100.0%) | 4 (100.0%) | 1 (25.0%) | 0 (0.0%) | 3 (75.0%) |
| 7 | 25 | 25 (100.0%) | 24 (96.0%) | 0 (0.0%) | 2 (8.0%) | 23 (92.0%) |
| 10 | 24 | 24 (100.0%) | 23 (95.8%) | 1 (4.2%) | 2 (8.3%) | 22 (91.7%) |
| 12 | 3 | 3 (100.0%) | 3 (100.0%) | 0 (0.0%) | 0 (0.0%) | 3 (100.0%) |
| 14 | 93 | 93 (100.0%) | 93 (100.0%) | 4 (4.3%) | 16 (17.2%) | 74 (79.6%) |
| 15 | 13 | 12 (92.3%) | 11 (91.7%) | 1 (8.3%) | 2 (16.7%) | 10 (83.3%) |
| 16 | 2 | 2 (100.0%) | 1 (50.0%) | 0 (0.0%) | 1 (50.0%) | 1 (50.0%) |
| 17 | 1 | 1 (100.0%) | 1 (100.0%) | 0 (0.0%) | 0 (0.0%) | 1 (100.0%) |
| 18 | 1 | 1 (100.0%) | 1 (100.0%) | 0 (0.0%) | 0 (0.0%) | 1 (100.0%) |
| 20 | 17 | 17 (100.0%) | 17 (100.0%) | 0 (0.0%) | 1 (5.9%) | 16 (94.1%) |
| 21 | 28 | 28 (100.0%) | 28 (100.0%) | 0 (0.0%) | 2 (7.1%) | 26 (92.9%) |
| 23 | 1 | 1 (100.0%) | 1 (100.0%) | 0 (0.0%) | 0 (0.0%) | 1 (100.0%) |
| 24 | 1 | 1 (100.0%) | 1 (100.0%) | 0 (0.0%) | 0 (0.0%) | 1 (100.0%) |
| 25 | 1 | 1 (100.0%) | 1 (100.0%) | 0 (0.0%) | 0 (0.0%) | 1 (100.0%) |
| 27 | 1 | 1 (100.0%) | 1 (100.0%) | 0 (0.0%) | 0 (0.0%) | 1 (100.0%) |
| 28 | 33 | 31 (93.9%) | 30 (96.8%) | 3 (9.7%) | 6 (19.4%) | 22 (71.0%) |
| 30 | 36 | 34 (94.4%) | 34 (100.0%) | 0 (0.0%) | 0 (0.0%) | 34 (100.0%) |
| 35 | 1 | 1 (100.0%) | 1 (100.0%) | 0 (0.0%) | 0 (0.0%) | 1 (100.0%) |
| 40 | 2 | 2 (100.0%) | 2 (100.0%) | 0 (0.0%) | 1 (50.0%) | 1 (50.0%) |
| 42 | 1 | 1 (100.0%) | 1 (100.0%) | 0 (0.0%) | 0 (0.0%) | 1 (100.0%) |
| 50 | 1 | 1 (100.0%) | 1 (100.0%) | 0 (0.0%) | 0 (0.0%) | 1 (100.0%) |
| 56 | 1 | 1 (100.0%) | 1 (100.0%) | 0 (0.0%) | 0 (0.0%) | 1 (100.0%) |
| 60 | 12 | 12 (100.0%) | 12 (100.0%) | 0 (0.0%) | 1 (8.3%) | 11 (91.7%) |
| 84 | 1 | 1 (100.0%) | 1 (100.0%) | 0 (0.0%) | 0 (0.0%) | 1 (100.0%) |
| 90 | 7 | 5 (71.4%) | 5 (100.0%) | 2 (40.0%) | 0 (0.0%) | 3 (60.0%) |
| Total | 320 | 313 (97.8%) | 308 (98.4%) | 12 (3.8%) | 34 (10.9%) | 270 (86.3%) |

**Notes:**
ᵃ Percentages in this column were calculated using all trials within the corresponding treatment-duration category as the denominator.

ᵇ Percentages in these columns were calculated using trials with at least one clearly specified assessment time point within the corresponding treatment-duration category as the denominator.

ᶜ A trial could contribute to more than one timing category when outcomes were assessed at multiple time points; therefore, the categories “before treatment completion,” “at treatment completion,” and “post-treatment” were not mutually exclusive and their percentages may sum to more than 100%.

ᵈ “Only treatment-completion time point clearly specified” indicates that all clearly reported assessment time points in a trial coincided with treatment completion. Entries recorded as **NK** were treated as unspecified and were not counted as additional assessment time points.

Assessment timing was classified by comparing each clearly reported measurement day with the treatment duration of the same trial. A measurement occurring earlier than the treatment duration was classified as **before treatment completion**, one occurring on the final treatment day as **at treatment completion**, and one occurring later than the treatment duration as a **post-treatment assessment**.
